# Multiple origins and modularity in the spatiotemporal emergence of cerebellar astrocyte heterogeneity

**DOI:** 10.1371/journal.pbio.2005513

**Published:** 2018-09-27

**Authors:** Valentina Cerrato, Elena Parmigiani, María Figueres-Oñate, Marion Betizeau, Jessica Aprato, Ishira Nanavaty, Paola Berchialla, Federico Luzzati, Claudio de’Sperati, Laura López-Mascaraque, Annalisa Buffo

**Affiliations:** 1 Department of Neuroscience Rita Levi-Montalcini, University of Turin, Turin, Italy; 2 Neuroscience Institute Cavalieri Ottolenghi, Orbassano, Turin, Italy; 3 Department of Molecular, Cellular, and Developmental Neurobiology, Cajal Institute -CSIC-, Spanish National Research Council, Madrid, Spain; 4 Brain Research Institute, University of Zurich Irchel, Zurich, Switzerland; 5 Department of Clinical and Biological Sciences, University of Turin, Turin, Italy; 6 Department of Life Sciences and System Biology, University of Turin, Turin, Italy; 7 Laboratory of Action, Perception and Cognition, Vita-Salute San Raffaele University, Milan, Italy; 8 Experimental Psychology Unit, Division of Neuroscience, IRCCS San Raffaele Scientific Institute, Milan, Italy; Duke University, United States of America

## Abstract

The morphological, molecular, and functional heterogeneity of astrocytes is under intense scrutiny, but how this diversity is ontogenetically achieved remains largely unknown. Here, by quantitative in vivo clonal analyses and proliferation studies, we demonstrate that the major cerebellar astrocyte types emerge according to an unprecedented and remarkably orderly developmental program comprising (i) a time-dependent decline in both clone size and progenitor multipotency, associated with clone allocation first to the hemispheres and then to the vermis(ii) distinctive clonal relationships among astrocyte types, revealing diverse lineage potentials of embryonic and postnatal progenitors; and (iii) stereotyped clone architectures and recurrent modularities that correlate to layer-specific dynamics of postnatal proliferation/differentiation. In silico simulations indicate that the sole presence of a unique multipotent progenitor at the source of the whole astrogliogenic program is unlikely and rather suggest the involvement of additional committed components.

## Author summary

Astrocytes are abundant cells of the brain essential to support and shape neuronal activity. They can be grouped in different subclasses based on their remarkable variety of morphologies, molecular profiles, and specialized functions. Although different astrocyte types likely display specialized interactions with distinct neuron categories, the different classes of astrocytes have only partially been unmasked. How astrocyte heterogeneity is ontogenetically achieved remains largely unknown. Here we approached this question by studying the development of the main astrocyte types of the cerebellum. The reconstruction of developmental lineages in the mouse embryo combined with proliferation studies and computational modeling demonstrate that cerebellar astrocyte types emerge according to an unprecedented and remarkably orderly developmental program. Embryonic progenitor cells produce either only a single astrocyte type or more types. These distinct astrocyte lineages display stereotyped architectures and recurrent modularities. Moreover, the generation of astrocytes follows a well-defined spatiotemporal pattern, defined by a time-dependent allocation of astrocytes to distinct cerebellar territories and an inside-out sequence of differentiation, coupled with a decline over time in both progenitor amplification and capability to produce distinct astrocyte types. These results provide the first evidence that an ontogenetic program, tightly regulated in space and time, determines astrocyte heterogeneity.

## Introduction

Increasing evidence supports the morphological, molecular, and functional heterogeneity of astrocytes across and within distinct regions of the developing and adult central nervous system (CNS) [1–3]. Although much progress has recently been made, we are still far from understanding how such heterogeneity emerges. Pioneer astrocyte lineage–tracing analyses at the single-cell level revealed that distinct progenitors generate different astrocyte types in the cerebral cortex, suggesting that clonal identity is the basis of astrocyte diversity [4–6]. Nevertheless, it remains unclear to what extent this applies to other brain regions. Most importantly, if and how the gliogenic potential of astroglial progenitors changes during development in terms of lineage composition and size of individual clones has not been explored.

A suitable model to address these questions is the cerebellum, given its simple cytoarchitecture with unique and well-characterized major astroglial types defined by distinct morphology, layering, marker expression, and functions [7,8] (S1 Table). These distinct types comprise fibrous astrocytes of the white matter (WMAs) and, in the overlying cortex, star-shaped bushy velate astrocytes of the granular layer (GLAs) and polarized Bergmann glia (BG), lined up in the Purkinje cell layer (PCL) [8,9]. Cerebellar astroglia are posited to originate from fourth ventricle radial glia (RG) cells [8,10–13] through their direct transformation into BG starting at embryonic day (E)14 and through the amplification of intermediate progenitors populating the nascent cerebellar parenchyma [14,15].

Here, by in vivo clonal analyses, we resolve the lineages of astrocytes in the cerebellum and show that astrogliogenesis occurs according to a well-defined spatiotemporal pattern from precursors whose fate potential declines over time. Our data disclose a stereotyped modularity in clone composition, revealing that unprecedented developmental rules govern astrogliogenesis at the clone level. We further demonstrate that postnatal proliferation ultimately defines clone size and that progenitors in the PCL can generate both BG and GLAs. Finally, in silico modeling suggests that cerebellar astrocyte heterogeneity does not emerge from a unique multipotent progenitor (MP) pool but may also require committed components.

## Results

### Embryonic waves of astrogliogenesis populate distinct cerebellar regions

**Fig 1:**
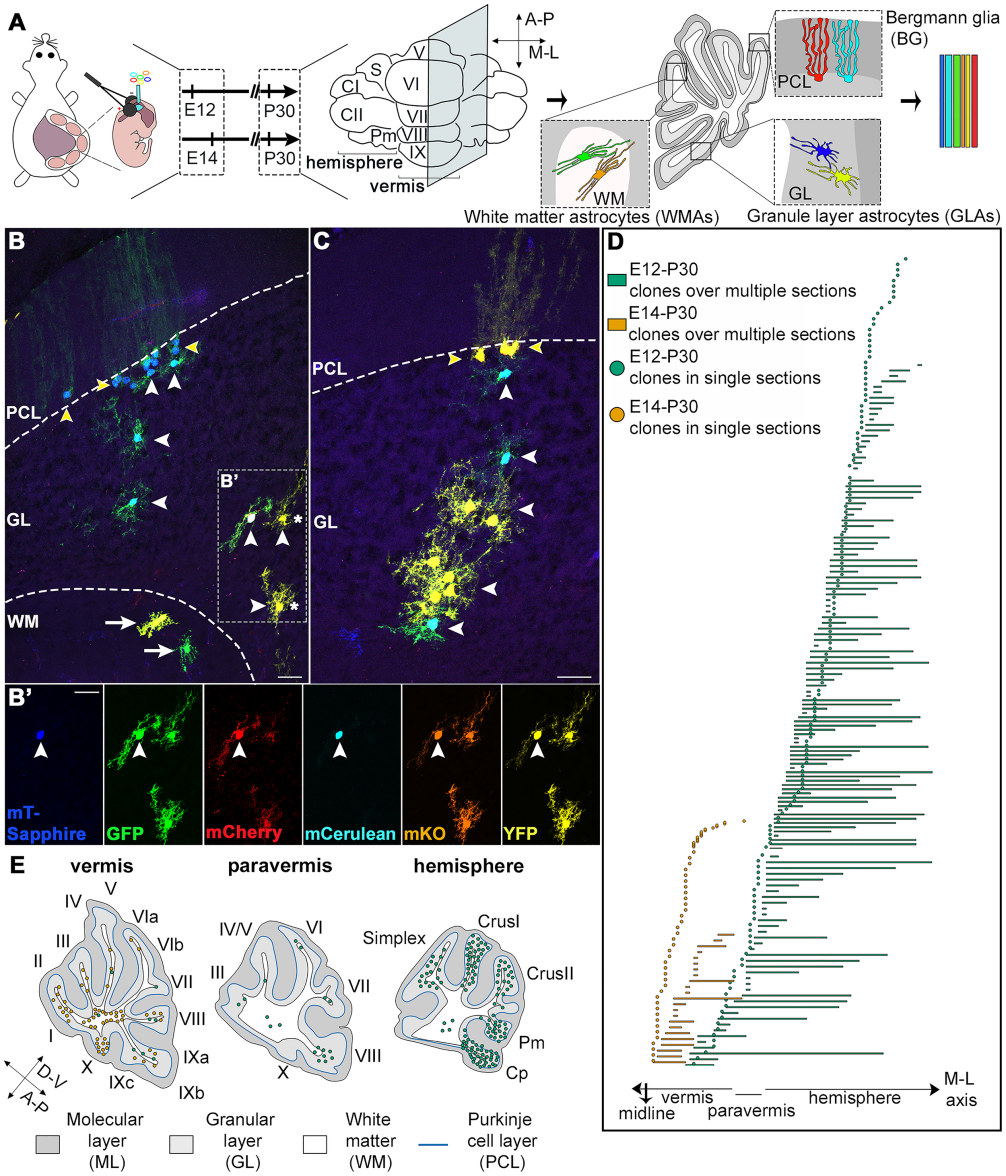
In utero StarTrack electroporations and clone allocation in the cerebellum. (A) Schematic representation of the experimental design. The hGFAP-StarTrack mixture was electroporated at E12 or E14, and clonal analysis was performed at P30. (B,C) StarTrack-labeled astrocytes are found in all cerebellar layers in P30 mice and comprise WMAs (white arrows), GLAs (white arrowheads), and BG (yellow arrowheads). In B’, 2 sister GLAs share the same combination of fluorescent proteins (asterisks), whereas the third GLA displays a different color combination, thus deriving from a different progenitor, even though it is very close to the other 2 GLAs. (D) Schematic representation of the relative M-L extension of each clone. E12-P30 clones (green) preferentially settle in the cerebellar hemispheres, whereas E14-P30 families (orange) are exclusively located in the vermis. Based on the cerebellar symmetry around the midline, all clones are projected on one-half cerebellum. The paravermis is defined as that region where lobule IX fades and lobule X is still present. (E) Diagrams are representative of clone distribution along the A-P axis. E12-P30 clones (green) are homogeneously distributed in all lobules of the hemispheres, whereas E14-P30 ones (orange) preferentially occupy the ventral vermis, including both anterior and posterior folia. Each dot corresponds to 1–2 clones. When clones are found in >1 lobule, they are repeatedly represented in each corresponding folium. Scale bars: 30 μm. A-P, antero-posterior; BG, Bergmann glia; CI and CII, CrusI and CrusII; Cp, copula pyramidis; D-V, dorso-ventral; E, embryonic day; GFP, green fluorescent protein; GLA, granular layer astrocyte; hGFAP, human glial fibrillary acidic protein; mCerulean, monomeric Cerulean; mCherry, monomeric Cherry; mKO, mKusabira Orange; M-L, medio-lateral; mT-Sapphire, monomeric T-Sapphire; P, postnatal day; PCL, Purkinje cell layer; Pm, paramedian; S, Simplex; WM, white matter; WMA, white matter astrocyte; YFP, yellow fluorescent protein.

To investigate the emergence of astroglial heterogeneity, we employed StarTrack [4], a system based on multiple plasmids that express up to 12 different fluorophores in the tagged cells and their progeny after piggyBac-driven stochastic integration into the genome. This allowed the classification of cells labeled by the same color combination as clonally related in the vast majority of cases (91%, as estimated by lumping error evaluation; see Methods). We labeled RG in the cerebellar ventricular zone (VZ; S1 Fig) and their astroglial descendants by StarTrack plasmids carrying the human glial fibrillary acidic protein (hGFAP) promoter after in utero electroporation (IUE) in the mouse fourth ventricle (Fig 1A–1C) either at E12, a fully neurogenic phase, or E14, reported as the beginning of gliogenesis [11,13,16,17].

At the end of cerebellar maturation (postnatal day [P]30), tagged cells were identified as astrocytes belonging to all typical cerebellar types comprising WMAs, GLAs, and BG (see Methods; Fig 1A–1C and S2 Fig and S1 Table). Rarely, astrocytes were also observed in cerebellar nuclei (cerebellar nuclei astrocytes [CNAs]). Thus, gliogenic RG populate the cerebellar VZ starting from early embryonic stages. Moreover, the apparent delamination of some E12-tagged hGFAP-expressing (^+^) precursors from the VZ already at E13 (S1B Fig) suggests that cerebellar gliogenesis starts earlier than E14.

Notably, E12- and E14-tagged clones displayed strikingly complementary medio-lateral (M-L) localizations. About 80% of E12-P30 clones settled in the hemispheres, whereas E14-P30 clones were exclusively (100%) in the vermis (Fig 1D). This lateral-to-medial shift in clone settlement is reminiscent of the sequence of birth of Purkinje neurons, which starts in the hemispheres and paravermis at earlier time points [14,16].

Clones also had a specific distribution along the antero-posterior (A-P) axis. E12-P30 clones were found in all folia of the hemispheres with no defined pattern in the paravermis/vermis (S3A Fig and Fig 1E). However, E14-P30 clones were more segregated in the anterior (I–V) and posterior-nodular (VIII–X) vermian lobules and rarely found in the central zone (lobules VI and VII, S3B Fig and Fig 1E). This pattern suggests that another VZ-derived astrogliogenic wave might populate the central structures. In summary, gliogenic RG are already present at E12 and produce astrocytes according to the spatiotemporal pattern of Purkinje cell (PC) genesis, although with a delay.

### Gliogenic ventricular progenitors undergo a developmental restriction in their differentiation potential

**Fig 2:**
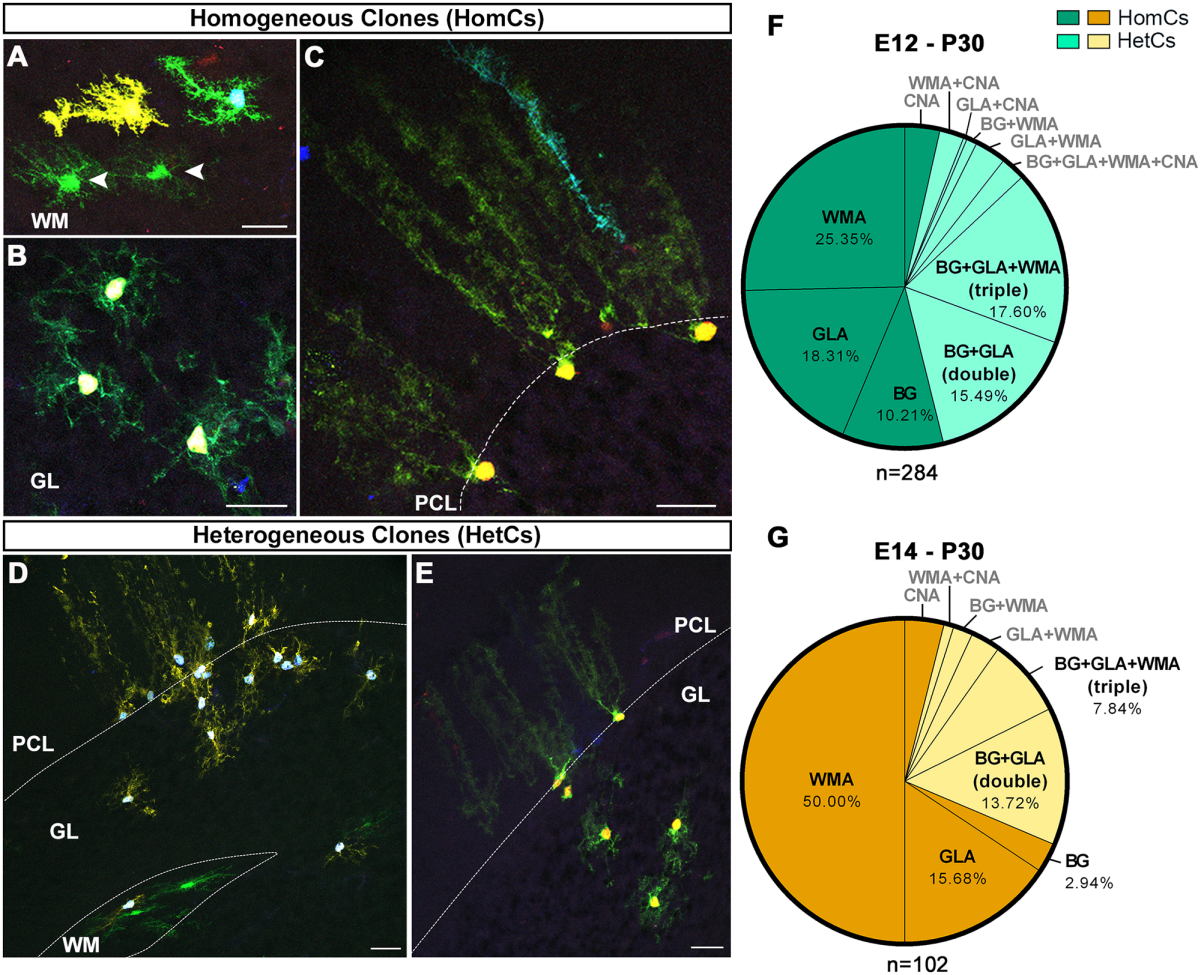
Composition of clones derived from E12 or E14 progenitors. (A-C) HomCs composed of WMAs (A; arrowheads indicate sister cells), GLAs (B), or BG (C) are generated by both early- and late-tagged progenitors. (D,E) The 2 major types of HetCs include triple clones made of BG+GLA+WMA (D) and double clones made of BG+GLA (E). (F,G) HomCs (dark shade) and HetCs (light shade) are produced in different frequencies by E12 (F) and E14 progenitors (G, *P* = 0.009). Among HetCs, BG+GLA+WMA clones are more numerous in E12-P30 compared to E14-P30 clones (*P* = 0.023), whereas BG+GLA clones do not vary (*P* = 0.748). Whereas WMA HomCs double in frequency in E14-P30 clones compared to E12-P30 clones (*P* < 0.001), BG HomCs decrease significantly (*P* = 0.021). Pies illustrate pooled data from 3–4 animals. (F) Minor fractions: CNA = 3.52%, WMA+CNA = 2.47%, GLA+CNA = 0.35%, BG+WMA = 1.06%, WMA+GLA = 3.17%, BG+GLA+ WMA+CNA = 2.47%. (G) Minor fractions: CNA = 3.92%, WMA+CNA = 0.98%, BG+WMA = 1.96%, WMA+GLA = 2.94%. *P* values are calculated with Fisher’s exact test. *n* = number of clones. Green = E12-P30 clones; orange = E14-P30 clones. Scale bars: 30 μm. BG, Bergmann glia; CNA, cerebellar nuclei astrocyte; E, embryonic day; GL, granular layer; GLA, granular layer astrocyte; HetC, heterogeneous clone; HomC, homogeneous clone; P, postnatal day; PCL, Purkinje cell layer; WM, white matter; WMA, white matter astrocyte.

We identified 2 main clone types: homogeneous clones (HomCs), formed by astrocytes of the same type (Fig 2A–2C), and heterogeneous clones (HetCs), including distinct astrocyte types (Fig 2D and 2E). Both clone types were generated at the 2 examined stages, although with different frequencies. E12 progenitors produced similar proportions of HomCs and HetCs, whereas E14 progenitors predominantly generated HomCs, suggesting restriction in the progenitor differentiation potential (Fig 2F and 2G). HomCs for each astroglial type were found at both time points. However, compared to E12-P30 clones, the E14-P30 WMA HomCs doubled in frequency (Fig 2F and 2G), whereas BG HomCs decreased to one-third. Among E12-P30 HetCs, 17.6% comprised 3 astroglial types (BG+GLA+WMA, triple clones; Fig 2D and 2F), whereas this proportion halved in E14-P30 clones (Fig 2G, 7.8%). On the other hand, HetCs including both BG and GLAs (BG+GLA, double clones, Fig 2E) were similarly represented in both data sets (Fig 2F and 2G). CNA HomCs and other kinds of HetCs including almost any combination of astrocyte types were found at both time points, but in very limited numbers and without overt changes (Fig 2F and 2G). Therefore, they were not considered for detailed quantitative analyses. Interestingly, although HomCs were overall more represented, 90% of all cortical astrocytes belonged to HetCs at both time points (S4 Fig), indicating that HetCs contain more cortical cells per clone than HomCs. Similarly, WMAs predominantly belonged to HetCs after E12 IUE. Yet, most WMAs were part of HomCs in E14 experiments.

Thus, the variety of cerebellar astrocytes derives from RG that, at both E12 and E14, are capable of producing distinct clone types, comprising either HomCs or HetCs composed of only cortical astroglia or including also WMAs. Triple clones decrease during development, consistent with a restriction in progenitor differentiation potential. In parallel, WMA HomCs increase, suggesting a lineage segregation of WM fates from those of cortical astrocyte types.

### Astrocyte clone size is affected by spatiotemporal factors and increases with the degree of clone heterogeneity

**Fig 3:**
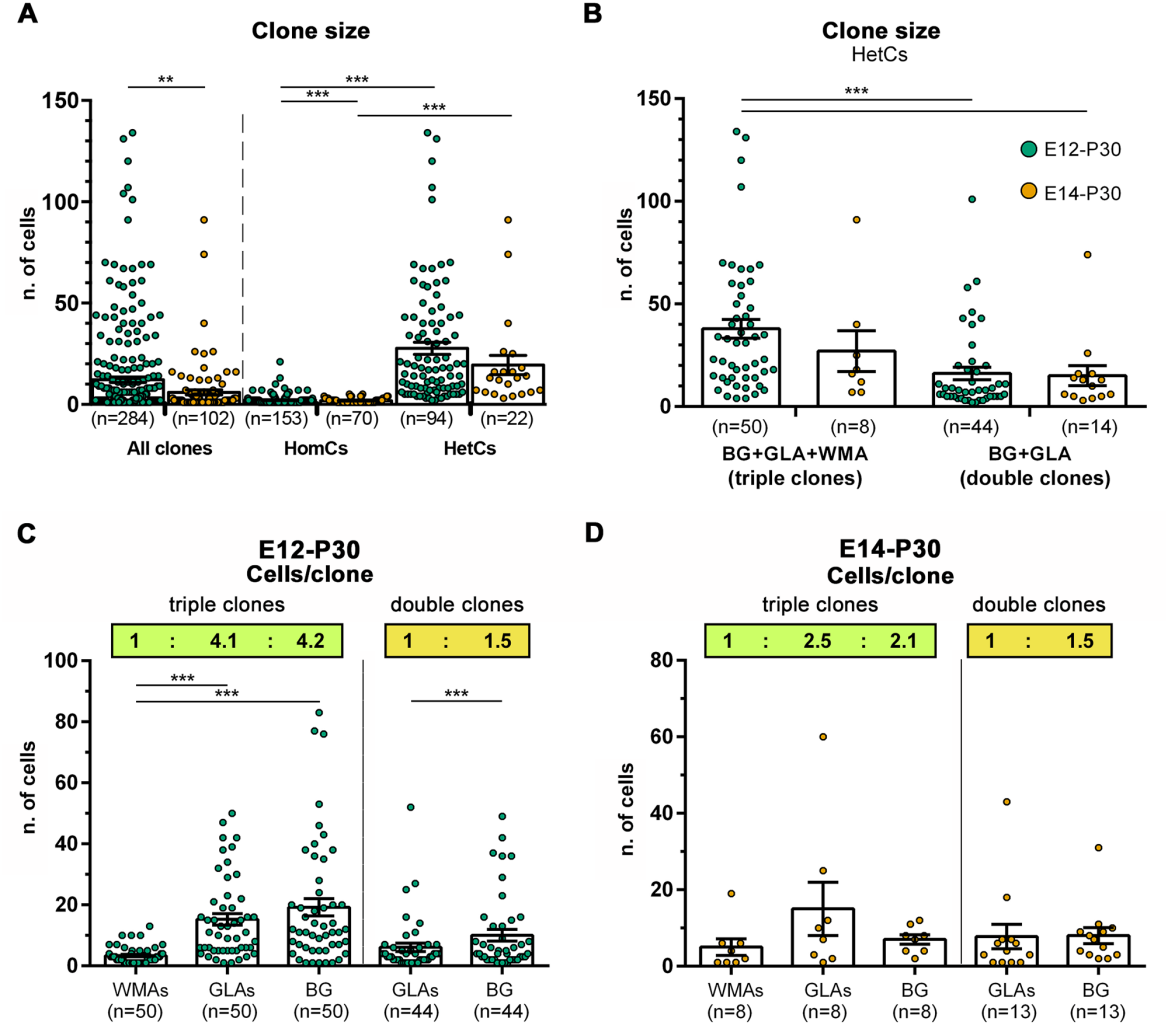
Quantitative analyses of clone size and stoichiometry of clone composition. (A,B) Scatterplots of clone size. (A) E12-P30 clones (green) are overall bigger than E14-P30 clones (orange). This same trend is maintained when comparing HomCs and HetCs within each time point (*P* < 0.001). E12-P30 HomCs contain more cells compared to E14-P30 clones, whereas HetCs’ size changes do not reach statistical significance (*P* = 0.081). (B) Triple BG+GLA+WMA clones are the biggest clone type and tend to be smaller when generated later (*P* = 0.317). Double BG+GLA clones are smaller than triple clones and display the same average size in the 2 data sets (*P* = 0.750). (C,D) Scatterplots show the number of distinct astroglial types in triple and double clones after E12 (C) or E14 (D) IUE. The insets report the clone-wise stoichiometry, rounded to the first decimal, which shows a joint expansion of GLAs and BG compared to WMAs in triple clones, whereas in double clones, BG prevail over GLAs. **, *P* < 0.01; ***, *P* < 0.001; *P* values are calculated with GEE analysis. *n* = number of clones. The numerical data used in the figure are included in S1 Data. BG, Bergmann glia; E, embryonic day; GEE, generalized estimating equations; GLA, granular layer astrocyte; HetC, heterogeneous clone; HomC, homogeneous clone; IUE, in utero electroporation; WMA, white matter astrocyte.

The number of cells per clone (clone size) was markedly lower in E14-P30 clones compared to E12-P30 families (Fig 3A; E12-P30, 14.58 ± 1.46 cells/clone; E14-P30, 6.05 ± 1.35 cells/clone). Moreover, HomCs typically consisted of only 1–2 cells (Fig 3A and S5A and S5B Fig), with E14-P30 clones being significantly smaller than E12-P30 clones (Fig 3A). HomCs were remarkably distinct from HetCs, which overall ranged from 3 to 134 cells. Yet, E14-P30 HetCs also displayed a clear trend to be smaller (Fig 3A; E12-P30, 30.2 ± 3.03; E14-P30, 19.41 ± 4.69), although without statistical significance. Among HomCs, WMA clones contained fewer cells than the other types (S5A and S5B Fig). Furthermore, single-cell families constituted about half of E12-P30 and E14-P30 HomCs and dominated among WMAs and GLAs (S5C–S5F Fig). Conversely, BG HomCs mostly included more than 1 cell (S5E and S5F Fig), suggesting a higher amplification of this lineage.

Triple clones were the largest, with an average size of 37.90 ± 4.57 cells/clone for E12-P30 cells that tended to decline in E14-P30 clones (27.00 ± 9.91; Fig 3B). E12-P30 double clones were smaller than triple clones and showed a comparable average size (approximately 15–16 cells) regardless of their time of origin (Fig 3B). In summary, HomCs amplify very little compared to their heterogeneous counterpart, thus showing that clone size positively correlates with the degree of clone heterogeneity. Moreover, progressing from early hemispheric to late vermian astrogliogenesis, clones decrease in size. This decrease may be due both to the larger contribution of very small HomCs (mainly WMAs) at later developmental stages and to a progressive decline in the cell amplification potential.

### HetC composition reveals a consistent stoichiometry of astroglial types

To address the existence of developmental programs controlling clone structure, we investigated the possible presence of a recurrent architecture in the composition of HetCs by examining the contribution of each astroglial type to HetCs. At the population level, in E12-P30 triple clones, WMAs were significantly fewer than GLAs and BG (Fig 3C). However, these latter types appeared in very similar numbers, indicating broad and parallel amplification of cortical astrocytes and limited expansion of WMAs. We next addressed whether the WMA:GLA:BG ratio at the population level was also represented within the clones. The ratio computed clonewise was 1: 4.069 ± 0.148: 4.220 ± 1.150, thus confirming a similar contribution of the cortical types (BG:GLA ≈ 1:1; *P* = 0.769) and their consistent predominance over WMAs at the clone level. This pattern was confirmed also in triple E14-P30 clones (1 WMA: 2.469 ± 1.342 GLAs: 2.077 ± 1.501 BG; *P* = 0.073, Fig 3D).

Although not always evident at the population level (Fig 3C and 3D), double clones also displayed a specific stoichiometry in which BG outnumbered GLAs in the same proportion for both clone groups (GLA:BG, E12–P30, 1: 1.519 ± 1.140 versus E14-P30, 1: 1.554 ± 1.232, *P* > 0.999). Further, the prevalence of BG over GLAs was significantly higher in double clones than in triple clones (BG:GLA ratio, *P* < 0.001 for both E12-P30 and E14-P30 clones). Altogether, these data reveal a remarkably consistent architecture in HetCs, supporting distinct amplification dynamics for cortical and WM types within triple clones and for sister BG and GLAs across double and triple clones.

### Clone dispersion follows clone size and is constrained by lobule morphogenesis

**Fig 4:**
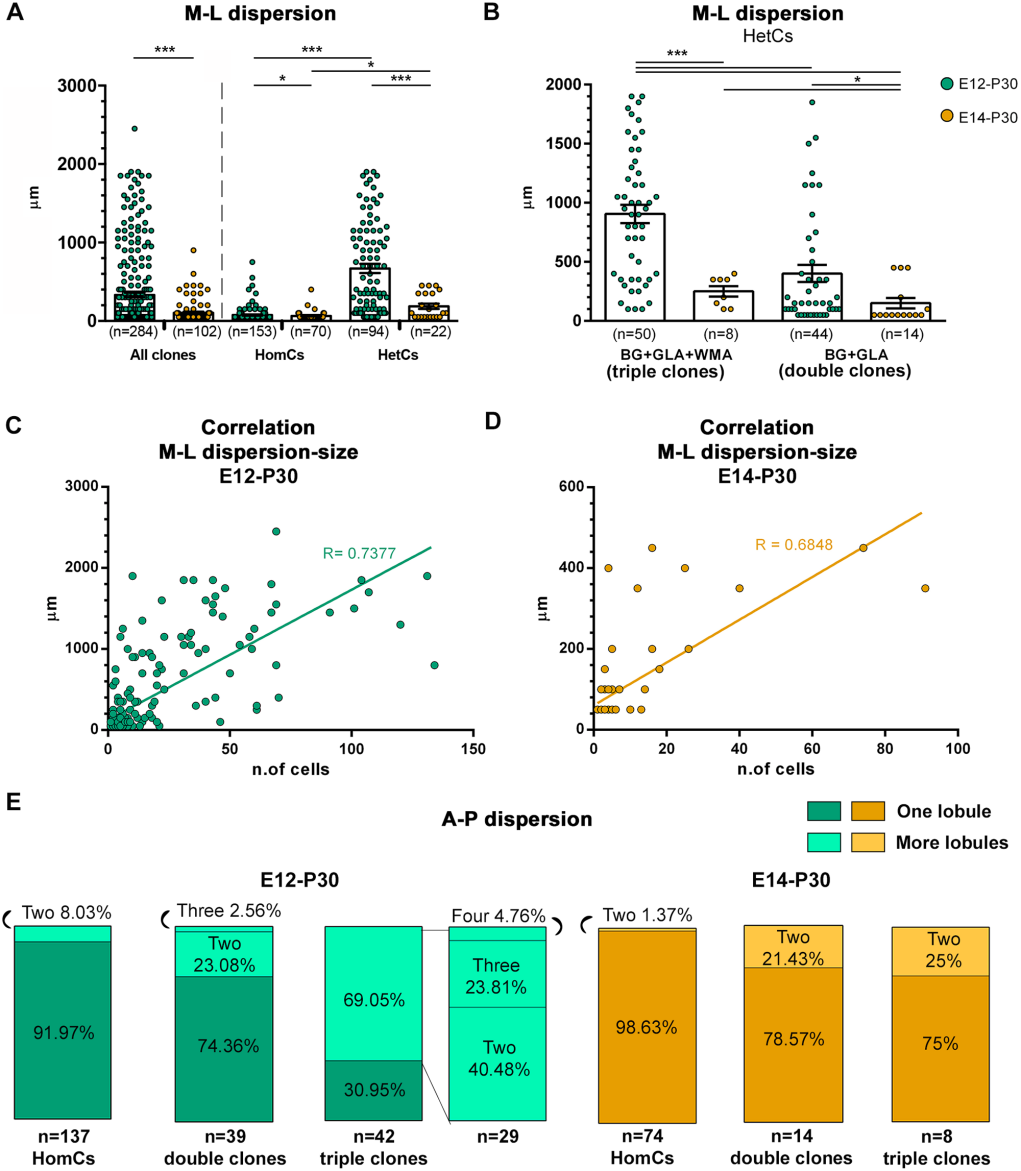
Quantitative analysis of clone dispersion along the M-L and A-P axes. (A,B) The M-L dispersion of clones tagged at E12 (green) or E14 (orange) is estimated as the longest intraclone cell distance along the M-L axis. (A) Scatterplots show that dispersion greatly decreases in E14-P30 clones compared to E12-P30 clones. HomCs are mostly found in a single cerebellar section, whereas HetCs are much more dispersed. (B) Among HetCs, triple clones are more expanded than double clones in both populations. Namely, E12-P30 triple clones are the most dispersed. (C,D) Correlation analysis shows a positive correlation between clonal size and M-L dispersion in both E12-P30 (C, *n* = 254 clones) and E14-P30 (D, *n* = 92 clones) populations (*P* < 0.001). (E) Distribution along the A-P axis of E12-P30 (green) and E14-P30 (orange) clones found in lobules. HomCs and double clones tagged at E12 mostly settle in a single lobule, whereas triple clones are often found in more than one lobule. E14-P30 clones are overall found in one lobule. Within lobules, cortical cells of HetCs were mostly found on the same side (i.e., lobular wall) divided by the WM (67% and 86% of E12-P30 and E14-P30 clones, respectively). *, *P* < 0.05; ***, *P* < 0.001; *P* values are calculated with GEE analysis. *n* = number of clones. The numerical data used in the figure are included in S1 Data. A-P, antero-posterior; BG, Bergmann glia; E, embryonic day; GEE, generalized estimating equations; GLA, granular layer astrocyte; HetC, heterogeneous clone; HomC, homogeneous clone; M-L, medio-lateral; WM, white matter; WMA, white matter astrocyte.

To shed further light on how astroglia contribute to cerebellar morphogenesis, we analyzed the dispersion of individual clones. M-L dispersion profoundly decreased in E14-P30 compared to E12-P30 families at the population level and also within the clone types (Fig 4A). HetCs were much more dispersed than HomCs (Fig 4A), and triple clones were more spread than double ones (Fig 4B). Thus, M-L clone dispersion increases according to the degree of clone heterogeneity and decreases as development progresses, resembling the reduction in clone size. Indeed, correlation analysis between size and dispersion confirmed a significant association (Fig 4C and 4D), pointing to cell number as a primary factor determining clone dispersion.

Analysis of A-P distribution (i.e., clone allocation in distinct lobules) showed that clones mostly localized in single lobules (Fig 4E), with the exception of E12-P30 triple clones, which predominantly dispersed in 2 to 4 contiguous folia. Thus, clones displayed an overall limited A-P dispersion. Moreover, HetCs’ cortical components were virtually always restricted to one of the 2 walls of a lobule. Taken together, clones broadly expand along the M-L axis according to their size but are largely confined within single lobules. Hence, astroglial clones rarely traverse the border of fissures between lobules, suggesting that most astroglial amplification occurs locally after fissure establishment.

### HetCs are formed by a modular architecture

**Fig 5:**
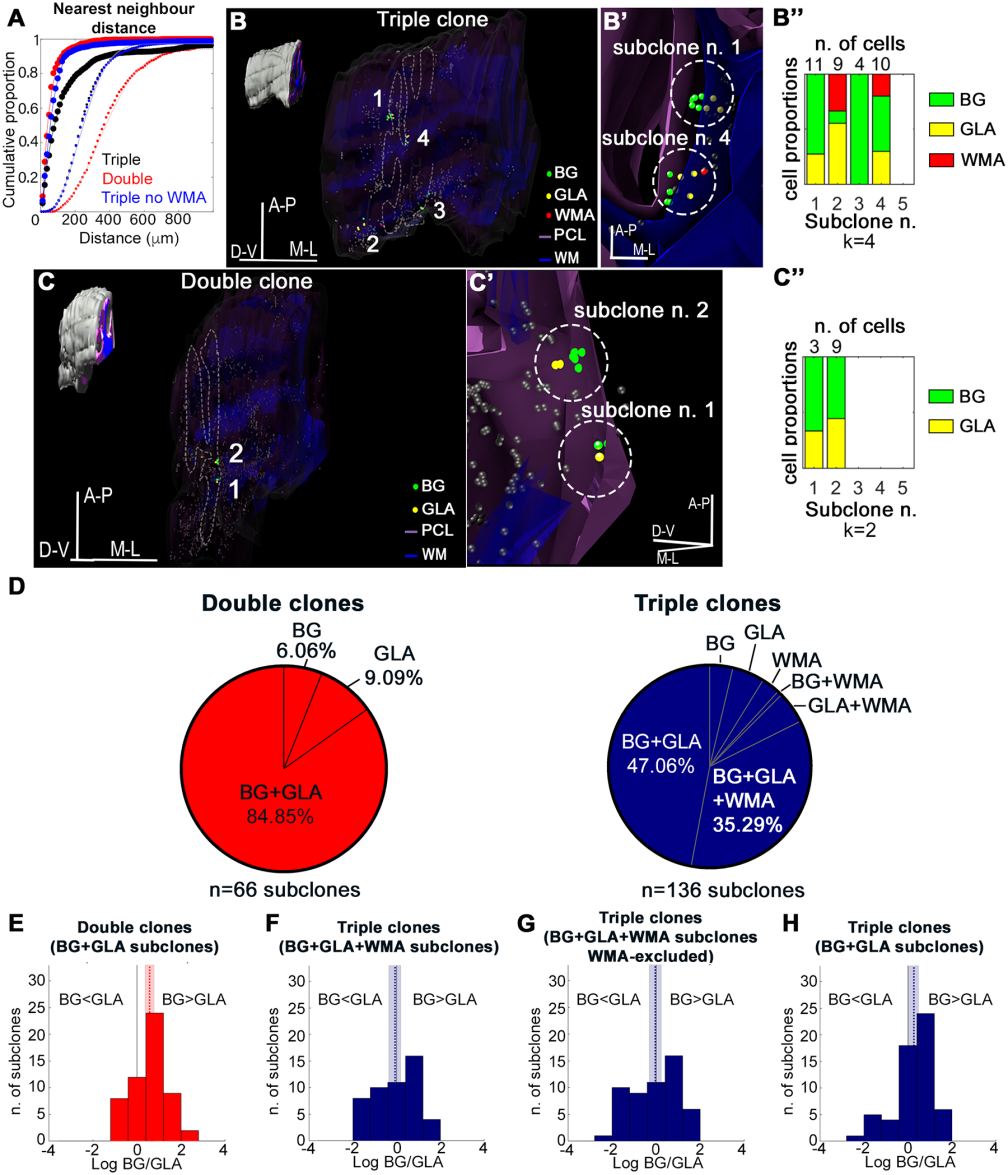
Modularity of HetCs. (A) Cumulative proportions of intracluster, cell-to-cell NND. Both double (red) and triple (blue and black) clones show a high degree of clustering when their distributions (large symbols) are compared to random distributions (small symbols; *P* < 0.001). Symbols represent empirical proportions. Continuous lines are the fitted curves. (B,C) Cluster analysis confirms the presence of subclones in both double and triple clones. The 3D reconstructions of a representative triple (B) and double (C) E12-P30 clone highlight the presence of spaced-out subclones composed of BG and GLAs; (B) also contains WMAs. (B’, C’) Higher magnifications of representative subclones (dashed circles) of the clones in (B) and (C). Gray spheres indicate cells of other clones. Plots in (B”) and (C”) show the number (k) and composition (cell type and number of cells/subclone) of subclones identified in the representative triple (B) and double (C) clones, respectively. Each subclone is arbitrarily associated to a number (subclone n.). (D), Analysis of subclone composition reveals that most (>80%) subclones in both double (red) and triple (blue) clones comprise both BG and GLAs. (E-H), BG:GL ratio distributions in subclones comprising both BG and GLAs. In double clones (E, number of subclones = 55), BG prevails over GLAs (mean ratio = 1.772, equivalent to 0.572 log units, *P* < 0.001). Subclones in triple clones containing WMAs (F, number of subclones = 49) show no predominance of either BG or GLAs (mean ratio = 0.930, −0.073 log units, *P* = 0.580), even when WMAs are excluded (G, number of subclones = 53; mean ratio = 0.963, −0.038 log units, *P* = 0.843), whereas subclones containing only BG and GLAs (H, number of subclones = 64) again display a prevalence of BG over GLAs (mean ratio = 1.310, 0.270 log units, *P* = 0.017). Number of bins = 10. In each plot, the vertical solid line indicates 1:1 ratio, the vertical dotted line indicates the mean ratio, and the light-colored area indicates the 95% bootstrap confidence interval of the mean. *P* values are computed with Wilcoxon signed rank test against zero. The numerical data used in panels (A,E,F,G,H) are included in S1 Data. A-P, antero-posterior; BG, Bergmann glia; D-V, dorso-ventral; GLA, granular layer astrocyte; HetC, heterogeneous clone; M-L, medio-lateral; NND, Nearest Neighbor Distance; PCL, Purkinje cell layer; WM, white matter; WMA, white matter astrocyte.

A 3D inspection of HetCs revealed that clones contained spaced-out cell clusters (subclones), suggesting a modular organization. Consistently, Nearest Neighbor Distance (NND) analysis (S2 Table) of E12-P30 HetCs showed a significant degree of clustering in both double and triple clones, as disclosed by their different distributions as compared to random distributions (Fig 5A). Interestingly, the cortical component (BG and GLAs) was comparable in triple and double clones, suggesting similar modalities of cortical colonization and reflecting a separate allocation of WMAs.

Cell clustering was further investigated through a cluster analysis (Fig 5B–5D, see Methods), revealing that (i) subclones formed both double and triple clones (2 and 3 subclones/clone on average, respectively); (ii) the mean number of cells/subclone was similar between double and triple clones (10 and 13, respectively); and (iii) subclone spatial extension, as indexed by the mean intra-subclone intercell distance, was smaller in double than in triple clones (about 200 μm and 360 μm, respectively).

To get insight on how subclones contributed to the corresponding clones, we analyzed their composition. In double clones, most subclones (85%, Fig 5D) comprised both BG and GLAs, with a clear predominance of BG over GLAs (mean BG:GLA ratio = 1.8, kurtosis = 0.1; Fig 5E). This pattern indicates that double clones are composed by a subclone typology with BG outnumbering GLAs—most commonly by approximately 2-fold—and suggests that double clones are customarily built upon unitary stereotyped modules. In triple clones, subclones were also formed mostly by both BG and GLAs (82%, Fig 5D), but the overall BG:GLA ratio was 1.1, in line with the corresponding clonewise stoichiometry. However, a dual pattern emerged according to whether or not subclones contained WMAs. In the former case (Fig 5F), the ratio distribution was uniform with no prevalence of BG or GLAs (mean = 0.9, kurtosis = −1.1), suggesting no modularity. This was confirmed also after excluding WMAs (mean = 1.0, kurtosis = −1.0; Fig 5G). By contrast, in the latter case (Fig 5H), the distribution was similar to double clones, with BG prevailing over GLAs (mean = 1.3, kurtosis = 0.2). Thus, loss of cortical modularity in subclones containing WMAs indicates that they follow different morphogenetic rules as compared to BG+GLA-only subclones. On the whole, these data suggest that the composition of parental clones reflects the structure of subclones, whether or not they are characterized by a modular architecture.

### Specific features and different fates of clones emerge postnatally

**Fig 6:**
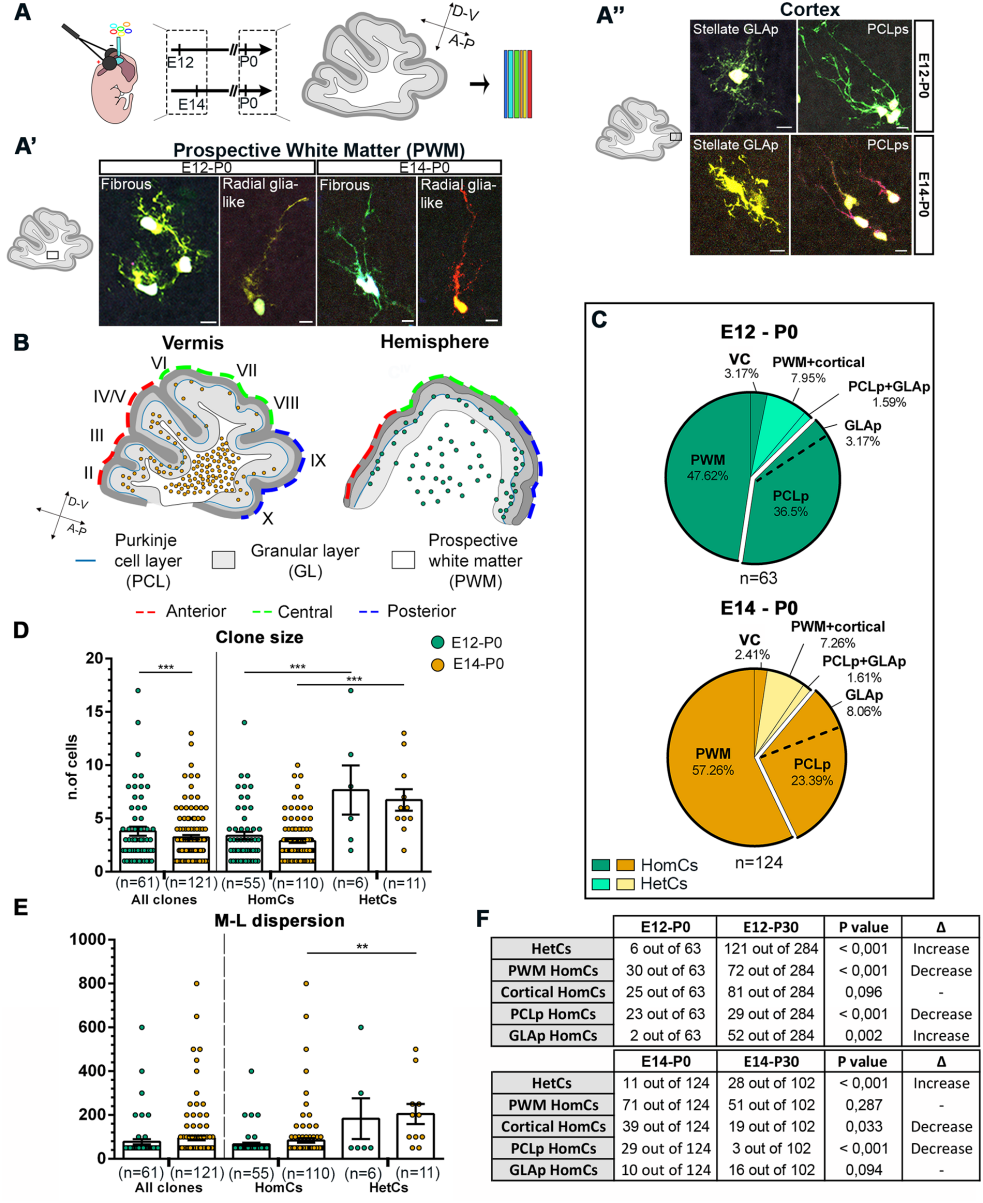
Analyses of clones at birth. (A) Schematic representation of the experimental design. IUE of the hGFAP-StarTrack mixture was performed at E12 or E14 and clonal analysis at P0. Representative examples of tagged cells at P0 comprising fibrous cells and RG-like cells in the PWM (A’), stellate GLA (GLAp), and PCLps of BG in the developing cortex (A”). (B) Schematic representation of clone distribution along the A-P and M-L axes. Early clones tagged at E12 (green) are settled in the hemispheres and are homogeneously distributed in all the developing lobules. Clones tagged at E14 (orange) are found in the vermis and allocate preferentially to anterior and, less frequently, to posterior lobules. (C) After both E12 and E14 IUE, HomCs are mostly found in the PWM at P0. A relevant proportion is also found in cortical layers (“exploded” sections), mostly as clones composed of PCLps. HetCs are still rare and include clones with cells in the two developing cortical layers (PCLp+GLAp) or in both PWM and cortex (PWM+cortical, comprising BGp+PWM, GLAp+PWM, and BGp+GLAp+PWM clones). Pies illustrate pooled data from 3 animals per time point. (D) Scatterplots show the size of E12 (green) and E14 (orange) clones at P0. Early- and late-tagged clones show statistically different sizes, despite the difference being negligible. At both time points, HomCs are smaller than HetCs. (E) Scatterplots show the M-L dispersion of E12- and E14-derived clones at P0. E12 and E14 clones do not differ in their M-L dispersion (*P* = 0.502). HetCs at both time points tend to be more dispersed than HomCs. (**, *P* < 0.01; ***, *P* < 0.001; *P* values are calculated with GEE analysis). Table in (F) summarizes the numbers of clones in each layer at P0 and P30 and the *P* values resulting from their comparisons by Fisher’s exact test. *n* = number of clones. Scale bars: 10 μm. The numerical data used in panels (D,E) are included in S1 Data. A-P, antero-posterior; D-V, dorso-ventral; E, embryonic day; GEE, generalized estimating equations; GL, granular layer; GLAp, granular layer astrocyte precursor; HetC, heterogeneous clone; hGFAP, human glial fibrillary acidic protein; HomC, homogeneous clone; IUE, in utero electroporation; M-L, medio-lateral; P, postnatal day; PCL, Purkinje cell layer; PCLp, Purkinje cell layer precursor; PWM, prospective white matter; RG, radial glia; VC, ventricular cell.

To elucidate the dynamics of clone allocation and amplification, we analyzed clones at birth (P0, Fig 6A). Similar to P30, E12-targeted cells were, for the most part, settled laterally, whereas E14-P0 clones were preferentially located medially (Fig 6B). Likewise, the A-P distribution of P0 clones essentially resembled that observed at P30 (Fig 6B).

All tested StarTrack-labeled cells displayed positivity for brain lipid–binding protein (BLBP), which marks astroglial precursors or negativity for the interneuron progenitors transcription factor paired box gene 2 (PAX2), or for the oligodendroglial lineage marker SRY-box 10 (SOX10) [15,18] (S6 Fig and Methods). Moreover, cells in the prospective white matter (PWM) expressed nuclear factor 1 A (NFIA), which labels astrocyte progenitors [19] (S6 Fig). Taken together, these data confirm that the tagged cells are astrocyte progenitors.

Most cells formed HomCs in the PWM (Fig 6C), including elements with the typical fibrous shape of WMAs and cells with a basal process extending from the soma towards the pial surface (Fig 6A’). These latter resembled RG detached from the VZ, formerly proposed as a source of BG [10]. A minor proportion of cells formed clones still at the VZ. About 30%–40% of clones (Fig 6C) were also found in the nascent cortex, where cells already displayed morphologies distinctive of bona fide radial BG precursors settled in the PCL (PCL precursors [PCLps]) or stellate GLA precursors (GLAps, Fig 6A”). HetCs were still rare and included a very small fraction of PCLp+GLAp clones, or cortical+PWM clones (Fig 6C).

At difference with P30 data, the average size of both E12-P0 and E14-P0 clones was still rather small (3.7 versus 3.2 cells/clone, respectively; Fig 6D). However, consistent with P30 results, HetCs already contained more cells than HomCs (Fig 6D). Similarly, HetCs tended to be more dispersed mediolaterally compared to HomCs (Fig 6E). However, E12-P0 and E14-P0 M-L dispersions did not differ yet (Fig 6E). Altogether, these results show that, at birth, layer allocation, size, and degree of dispersion are not yet achieved, whereas the positional choice of clones along the cerebellar axes is already defined.

In support of this view, comparison of P0 and P30 data showed that HetCs proportionally increased with time in the whole clone population for both E12- and E14-derived cells (Fig 6F). Interestingly, this correlated with a reduction of E12 PWM HomCs by half (Fig 6F), suggesting that the P0 hemisphere PWM still hosts a number of MPs capable of producing HetCs. Moreover, the fraction of P0 PCLp HomCs significantly decreased compared to P30 data for both E12- and E14-derived lineages (Fig 6F). Therefore, PCLp in both hemispheres and vermis may generate different astrocyte types.

### PCLps are the source of distinct astroglial types after birth

**Fig 7:**
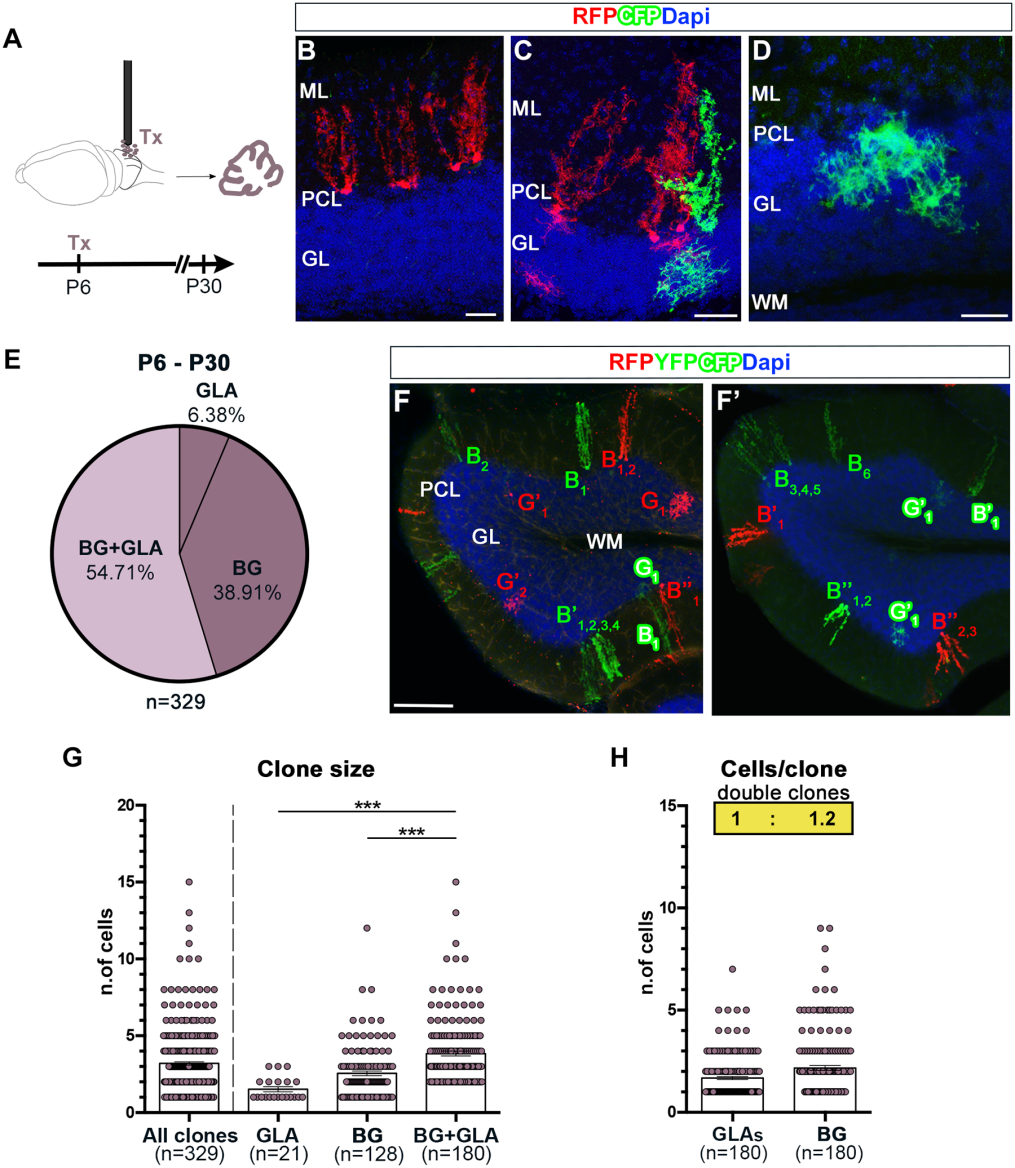
Analysis of PCLp progenies at P30. (A) Experimental design of superficial administration of Tx to induce R26R^Confetti^ recombination in radial GLAST^+^ precursors in the PCL (PCLps). (B-D) Analysis after serial sections’ reconstruction at P30 reveals the existence of sister astrocytes (i.e., expressing the same color) arranged in different clone types: BG clones (B), double clones composed of BG+GLA (C), and rare GLA clones (D). (E) Quantification of the relative proportion of each clone type derived from P6-tagged PCLps in lobule IV–V. Pies illustrate pooled data from 3 animals. (F,F’) Examples of clones distributed in 1 or 2 adjacent sections. (B = BG, G = GLA; superscripts indicate distinct clones; subscripts indicate sister cells). (G) Scatterplots of clone size. BG+GLA clones are bigger compared to clones composed of only BG or GLAs. (H) Scatterplots show the number of distinct astrocyte types in double clones. The insets report the clonewise stoichiometry, rounded to the first decimal, which highlights the prevalence of BG over GLA. ***, *P* < 0.001; *P* values are calculated with GEE analysis *n* = number of clones. Scale bars: 30 μm (B-D), 100 μm (F-F’). The numerical data used in panels (G,H) are included in S1 Data. BG, Bergmann glia; CFP, cyan fluorescent protein; DAPI, 4′,6-diamidino-2-phenylindole; GEE, generalized estimating equations; GL, granular layer; GLA, granular layer astrocyte; GLAST, glutamate aspartate transporter; ML, molecular layer; PCL, Purkinje cell layer; PCLp, Purkinje cell layer precursor; RFP, red fluorescent protein; Tx, tamoxifen; WM, white matter; YFP, yellow fluorescent protein.

To clarify whether PCLps generate different astrocyte types, we employed R26R^Confetti^ reporter mice [20] that enable the distinction of progenies derived from individual progenitors through stochastic and exclusive expression of 1 out of 4 fluorochromes. To induce recombination in PCLps, we applied tamoxifen (Tx) on the cerebellar surface [15] (Fig 7A) of GLAST^CreERT2/+^xR26R^Confetti/+^ (Confetti) mice at P6, when only progenitors in the PCL own a basal process. We adopted a protocol that allowed targeting of a limited number of PCLps to follow the progenies of individual progenitors (S7 Fig; see Methods). Analysis performed at P30 revealed that the tagged cells were GFAP^+^ astrocytes and included both BG and GLAs, which expressed cell type–specific markers (S8 Fig and S1 Table). Cells were then grouped in clones (see Methods) that resulted to be formed by only BG or both BG and GLAs (Fig 7B, 7C, 7F and 7F’). Clones formed by GLAs only (Fig 7D) were rarely observed and may derive from direct transformation of PCLps into GLAs. The fractions of double BG+GLA clones and BG-only clones were similar, indicating an overall comparable probability for a PCLp to proceed along the 2 lineages (Fig 7E). Double clones were significantly bigger than clones solely formed by BG or GLA (Fig 7G), consistent with the bigger size of StarTrack HetCs. Moreover, double clone average size (about 4.5 cells/clone) and spatial dispersion (about 90% within approximately 180 μm, intraclone intercell distance) were about half of those computed for cortical StarTrack subclones (see above section ‘HetCs are formed by a modular architecture’), consistent with tagging of individual progenitors. Notably, the BG:GLA ratio (Fig 7H) revealed a predominance of BG and displayed a value (1.2) intermediate to that of StarTrack triple and double clones.

Similar to VZ RG in the developing telencephalon, the choice of PCLps to self-renew and generate BG or produce a distinct progeny (i.e., GLAs) may be associated with different cleavage plane orientations during division, determining the inheritance of the radial process [21–23]. However, like basal RG [24], phosphorylated Vimentin (pVimentin)-labeled proliferative PCLps always displayed predominant horizontal divisions (S9 Fig) with a frequency (approximately 80%) not matching the proportions of double/BG clones. Thus, factors other than the cleavage plane orientation are likely to influence the PCLp cell fate. In summary, postnatal PCLps can generate GLAs and contribute BG+GLA clones.

### Astroglial precursors amplify and differentiate according to layer-specific dynamics after birth

**Fig 8:**
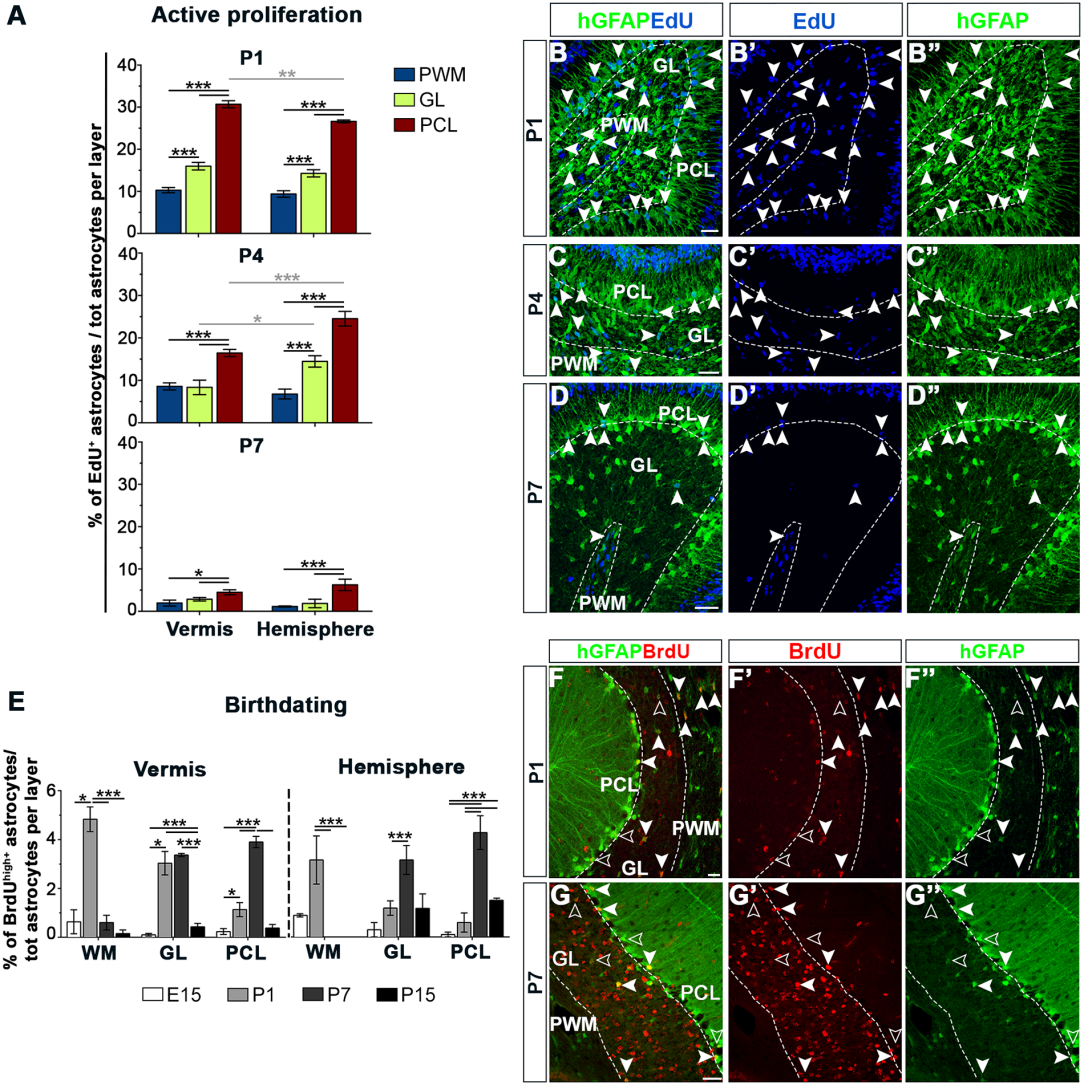
Proliferation and cell cycle exit of cerebellar astrocytes. (A-D) Analysis of active proliferation of astrocytes in different layers during early postnatal development. (A) Percentage of EdU-incorporating astrocyte precursors among total hGFAP^+^ cells subdivided per layers. EdU was administered 6 h before killing at P1, P4, or P7 to detect actively proliferating cells. At all analyzed time points, proliferation varies among layers and declines over time in both vermis and hemispheres. In B-D”, arrowheads point to double-labeled proliferating precursors, highlighting the overall reduction over time in the proliferation activity and the higher number of proliferating cells in the PCL compared to the other layers. (E-G”) Birthdating of astrocytes retaining a strong BrdU signal (BrdU^high^) after the completion of the maturation process (P30). The histogram in (E) shows that the highest percentages of BrdU^high+^ cells are observed in the first postnatal week, with minimal labeling before and afterwards. In detail, numerous WMAs exit the cell cycle already at P1, while GLAs and BG differentiate later. On the whole, this layer-specific pattern applies to both the vermis and the hemispheres (*P* = 0.139), where, however, differentiation delayed (PCL, *P* < 0.001). (F-F”,G-G”) Full and empty arrowheads point to astrocytes retaining a strong (BrdU^high^) or a diluted (BrdU^low^) BrdU signal, respectively. *, *P* < 0.05; **, *P* < 0.01; ***, *P* < 0.001; *P* values are calculated with GEE analysis. Plots represent data averaged from distinct animals. Scale bars: 30 μm. The numerical data used in panels (A,E) are included in S1 Data. BG, Bergmann glia; BrdU, bromodeoxyuridine; EdU, 5-ethynyl-2′-deoxyuridine; GEE, generalized estimated equations; GL, granular layer; GLA, granular layer astrocyte; hGFAP, human glial fibrillary acidic protein; P, postnatal day; PCL, Purkinje cell layer; PWM, prospective white matter; WM, white matter; WMA, white matter astrocyte.

The different sizes of cortical and WMA HomCs and the distinct contribution of astrocyte types to HetCs suggested different layer-specific proliferation rates. This hypothesis was addressed at early postnatal stages, during clone expansion and maximal cerebellar growth [25,26]. Proliferating astroglial progenitors were tagged with 5-ethynyl-2′-deoxyuridine (EdU) in hGFAP-GFP mice [27] (Fig 8A–8D”). EdU^+^hGFAP^+^ precursors exhibited layer-dependent proliferative behaviors in both vermis and hemispheres. PCLps divided extensively, showing a peak of proliferation at P1, which gradually decreased afterward. EdU^+^hGFAP^+^ cells in the PWM and granular layer (GL) followed the same trend, although with an overall lower proliferation rate (Fig 8A–8D”). A layer-dependent proliferation rate, declining over time, was confirmed when ongoing proliferation was assessed in StarTrack^+^ cells (S10 Fig). This analysis also indicated a slightly higher proliferative activity in E12-derived PCLps compared to E14 counterparts immediately after birth (S10 Fig). Cell cycle reentry analysis on the whole astrocyte pool (S11A–S11D”‘ Fig) showed that all astroglial precursors undergo an early proliferation burst until P4, with PCLps being the most proliferative cells even at later times, consistent with the high number of BG in P30 StarTrack clones. Thus, astroglial progenitors in the postnatal cerebellum undergo intense proliferation immediately after birth and expand according to layer-specific dynamics.

To complement these findings, we performed a birthdating analysis. Retention of high bromodeoxyuridine (BrdU) levels (BrdU^high^) in astrocytes at P30 after a single BrdU pulse identified cells that left the cell cycle approximately at the time of BrdU injection [28]. A remarkably similar layer-dependent differentiation pattern was observed in both vermis and hemispheres. When dividing astrocyte precursors were tagged at E15, rare BrdU^high+^hGFAP^+^ cells were found at P30, mainly in the WM (Fig 8E and S11E Fig), indicating that E15 progenitors hardly differentiate without further amplification. WMAs predominantly differentiated at P1 (Fig 8E-F”), whereas the bulk of BG and hemispheric GLAs differentiated at the end of the first postnatal week (Fig 8E). Vermian GLAs instead exited proliferation with a constant rate throughout the first 7 d of life (Fig 8E–8G”). BrdU^high^ cells were also found in hemispheric cortical layers when progenitors were tagged at P15, indicating a delay of astrocyte differentiation in this territory. Thus, shortly after birth, WMAs exit the cell cycle, showing a limited expansion phase. Conversely, GLAps continue to divide for a longer time. In addition, PCLps differentiate after an extended expansion during which they appear overall more proliferative compared to progenitors in other layers.

### Multipotent and lineage-restricted progenitors are likely to coexist in the E12 and E14 cerebellar VZ

**Fig 9:**
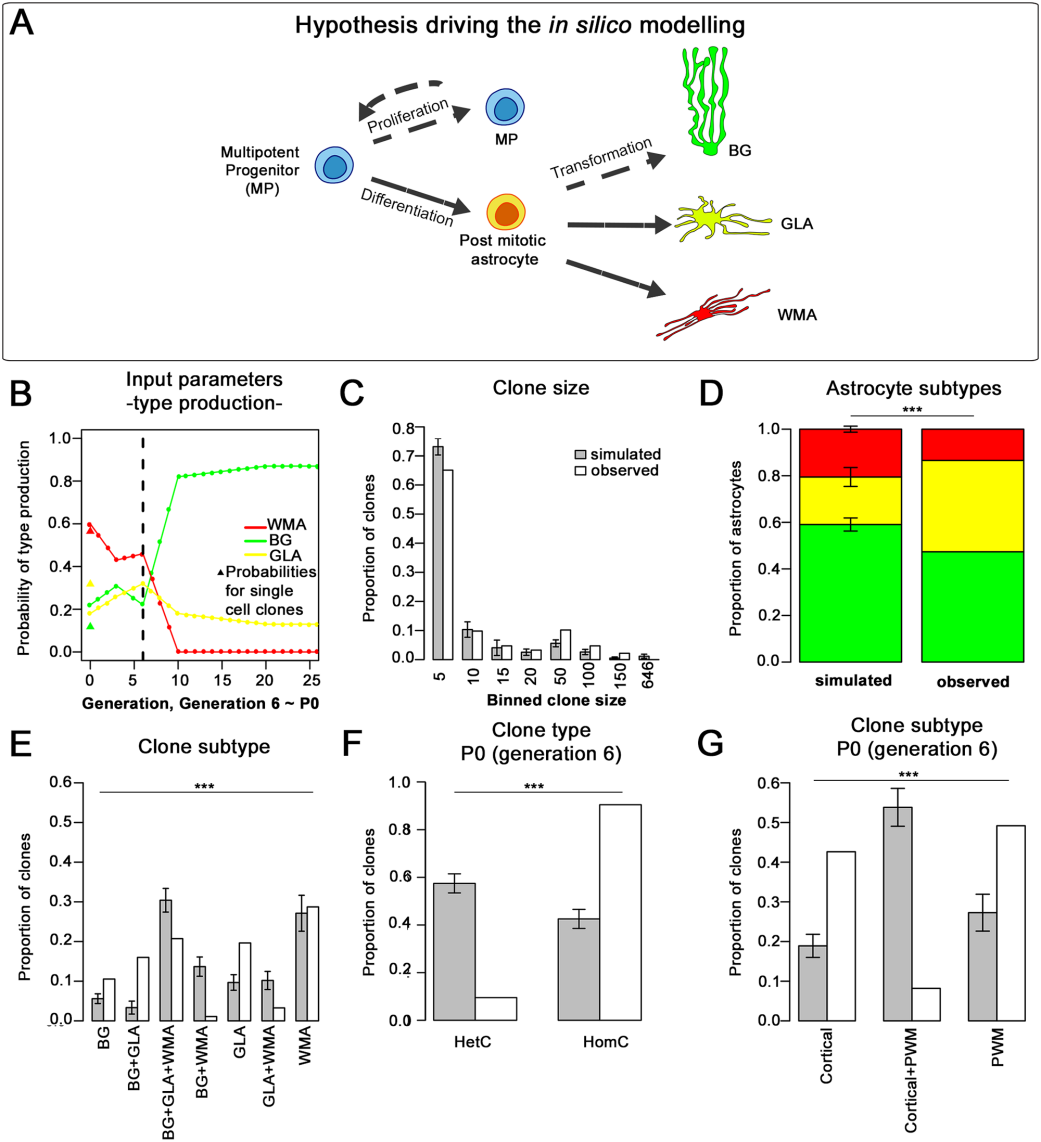
Rules and outcome of the simulation model applied to E12 lineages. (A,B) The schematic representation in (A) shows the distinct fate transitions allowed in the model (indicated by the arrows). The probability of MP proliferation is kept constant at 0.465 in the simulations of E12-P30 lineages. Each daughter cell of each division either remains an MP or differentiates into a postmitotic astrocyte. In this latter case, the probabilities of generating the distinct astrocyte subtypes (BG versus GLA versus WMA) are generation-dependently set according to the birthdating experiments performed in the hemispheres, as shown in (B). Histograms in (C-E) show the outcomes of the simulated lineages compared to the experimental data. Simulated clone sizes (C) appear quite similar to those of the observed clones. On the other hand, the proportions of astrocyte subtypes are not well represented, with too many WMAs and BG produced (D). Same color code as in (A). Similarly, the model fails to recapitulate the proportions of clone subtypes (E; E12-P30). (F,G) Simulated and observed lineages were compared at P0 (corresponding to generation 6). Too many HetCs (F) are found in simulated lineages compared to empirical clones, because of the generation of too many PWM+cortical clones at the expense of both cortical and PWM families (G). ***, *P* < 0.001; *P* values are calculated with chi-squared test. Cortical clones comprise PCLp HomCs, GLAp HomCs, and PCLp+GLAp HetCs; Cortical+PWM clones comprise PCLp+PWM, GLAp+PWM, and PCLp+GLAp+PWM HetCs. The numerical data used in panels (B-G) are included in S1 Data. BG, Bergmann glia; E, embryonic day; GLA, granular layer astrocyte; GLAp, granular layer astrocyte precursor; HetC, heterogeneous clone; HomC, homogeneous clone; MP, multiple progenitor; P, postnatal day; PCLp, Purkinje cell layer precursor; PWM, prospective white matter; WMA, white matter astrocyte.

Data show that the cerebellar VZ hosts gliogenic RG producing distinct clone types and whose differentiation potential changes with time and space. These findings could be explained by either distinct RG committed to different fates or by a homogeneous population of multipotent RG that stochastically make their fate choice. To tackle this issue, we tested the validity of a simple model assuming the existence of a single MP pool. To this aim, lineages were simulated in silico, cell fate choices were modeled in a probabilistic manner (Methods; Fig 9A), and the outcome was compared to empirical data. MPs, including both RG and all the precursors in the derived progenies, were assumed to maintain the same properties across layers and over time, whereas the probabilities of production of the distinct astrocyte subtypes were generation-dependently based on birthdating analyses (Fig 9B and S12A Fig).

This stochastic model produced clones whose size was in good agreement with the observed clones (Fig 9C and S12B Fig). However, it failed to reproduce the proportions of clone subtypes and astrocyte types. Indeed, in E12-P30 hemispheric lineages, none of the clone subtypes was correctly captured by the model, except for WMA HomCs, and too many WMAs and BG were produced (Fig 9D–9E). On the other hand, the simulations of E14-P30 vermian families properly reproduced the fraction of the sole double clones but failed with the others and produced too many BG (S12C–S12D Fig). The comparison between the simulated lineages at the generation corresponding to P0 and empirical E12-P0 or E14-P0 clones revealed that at this stage, the amount of simulated HetCs was much higher than expected, while HomCs were underrepresented. The model produced too many PWM+cortical HetCs at P0, at the expense of both cortical and PWM clones (Fig 9F and 9G and S12E–S12F Fig), suggesting a deviation from a model with a single MP already before birth. Overall, these results suggest that the single-MP hypothesis can be ruled out under the assumptions of simple proliferative kinetics and, rather, point towards some additional lineage-restricted progenitor types in the cerebellar VZ.

## Discussion

The spatiotemporal generation pattern of distinct astrocyte types and the rules governing the formation of astroglial lineages are poorly understood. By studying the cerebellum, we found that astrogliogenesis occurs through distinct lineages comprising multiple or single astrocyte types according to an orderly developmental program based on a prominent recurrent modularity in the heterogeneous lineages.

In the mature cerebellum, E12- and E14-derived clones were distributed along the M-L and A-P axes according to a well-defined pattern established at birth and linked to the organization of the cerebellar territory. Clones from E12-tagged RG were found predominantly in the cerebellar hemispheres through all folia, whereas E14-derived RG gave rise to vermian clones, mostly confined to the anterior and posterior lobules. The consistency of this pattern across samples and the rather diffuse M-L and A-P targeting of the VZ by IUE exclude a sample bias. These results are compatible with a developmental scheme where E12 astrogliogenic RG preferentially populate the hemispheres, while distinct RG, still rare at E12, become dominant at later stages, likely by amplification, and populate the vermis. Thus, consecutive waves of RG amplification and detachment seem to occur. This scheme is reminiscent of the organization of cerebellar PCs born on different days and is in line with previous histological findings [11]. Indeed, birthdating studies in rats [14] and genetic fate mapping in mice [16] showed that PC production starts with neurons that, similar to early astrocyte lineages, settle in the hemispheres and paravermis. Along the A-P axis, E14-derived clones were rarely found in the vermian central folia, which might become populated by a distinct wave of astrogliogenesis not sampled in this analysis.

Size and M-L dispersion of individual clones jointly decreased transiting from early hemispheric to later vermian astrogliogenesis and with clone homogeneity. At birth, all clones were small and had similar dispersions and cell type composition, showing that differences predominantly emerge postnatally during cerebellar growth, likely driven by proliferation of pioneer progenitors settled in the nascent parenchyma, as also shown in the forebrain [29]. We also noted that clones were commonly found in a single lobule, in agreement with proliferation occurring within the boundaries of early established fissures anchoring centers, as formerly described for granule cells [25]. E12 triple HetCs were the only exception, showing dispersion across adjacent lobules in the hemispheres. This may depend on delayed hemispheric fissure formation [30] and/or on the presence of numerous PWM multipotent pioneer progenitors. E12 clones were also overall bigger in size compared to E14 families. This, on the one hand, is consistent with the higher proportion of small HomCs in the E14 progeny. On the other hand, E12 progenitors might also be endowed with an increased proliferative capability, which declines in a time-dependent manner in later-generated families, as shown for neurogenic progenitors [31]. Indeed, on the whole, our data suggest that in the hemispheres, a slightly higher proliferative activity in PCLps at defined postnatal time points, together with protracted proliferation leading to a delayed exit from the cell cycle, might contribute to the distinct features of early and late clones. These different behaviors may depend on cell-intrinsic properties or region-specific environmental factors influencing clone proliferation. In this respect, tissue expansion does not appear as the major regulator of clone size, because the vermis, where clones are small, has a volume double of that of each hemisphere. Alternatively, small vermian clones may just reflect a higher number of RG available to support the full territorial occupation of the vermis. Similarly, the major dispersion of E12 clones may be supported by a specific migratory ability of these early-born progenitors and/or by specific environmental cues, as described to occur in the cerebral cortex [32]. Moreover, radial migration along RG trajectories allocates astrocytes to defined segmental domains according to a temporal ventral-to-dorsal sequence in both the spinal cord and forebrain [1,33]. In the cerebellum, instead, astrocyte allocation over time follows a latero-medial shift that intersects with the formation of the A-P lobular units. Notably, temporal patterning also appears to influence features (e.g., size, dispersion, see also below) of cerebellar astrocyte clones. However, it remains to be determined whether astrocyte spatial segmentation confers a specific functional specialization to the cells.

A characteristic feature of clones was their diverse cell type composition, including homogeneous and heterogeneous lineages constituted of all, 2, or just 1 astroglial type. This highlights a remarkable multipotency of gliogenic RG, which is expressed in terms of both lineages and astroglial types at the examined time points. However, multipotency declines during cerebellar morphogenesis, since triple HetCs became less frequent concomitantly with an expansion of WMA HomCs. The latter finding, as discussed above for size, highlights region-specific differences within the cerebellum. However, it also conforms to the notion of time-dependent reduction in the differentiation potential of neurogenic RG [34,35]. Further, it is suggestive of a progressive separation of cortical and WM lineages, in line with the segregation of astroglial clones found in the neocortex [4]. However, unlike the neocortex, where both cortical and WM clones essentially exhibit a homogeneous composition [4], HetCs consistently comprised the majority of cerebellar astrocytes. The poor morphological diversity of neocortical parenchymal astrocytes may have partly masked clone heterogeneity, which was instead observed in olfactory bulb astroglial clones [36]. These discrepancies may alternatively reflect area-specific differences in RG competence or the occurrence of multipotent gliogenic RG only at early stages of embryonic development. In the cerebellum, HomCs included numerous single-cell clones, consistent with direct derivation of astrocytes from RG without proliferation. This was so far postulated for BG generation through RG translocation [11,12,14], but our data suggest that direct transformation may occur for all astrocyte types and may be especially frequent for WMAs. Some astroglial cerebellar lineages may also contain neuronal derivatives hidden in our data by astrocyte-specific promoter regulation of reporter expression. Based on evidence that postnatal PWM progenitors generate both M-L interneurons and WMAs [15,37,38], sibling interneurons may belong to WMA-containing triple and/or HomCs, although broader clonal relationships between astroglia and other cerebellar neurons cannot be excluded [39].

What mechanisms drive RG along distinct homogeneous or heterogeneous astroglial lineages? The observed behaviors could be produced by a homogeneous population of multipotent RG that undergo stochastic changes in competence over time. Such a model has been proposed [34,35] and confirmed by computational analyses [40,41] for temporal switches in RG fate potency along neuronal lineages. Alternatively, as also suggested for neuronal lineages [42,43] and supported by in silico simulations [31], clone heterogeneity may originate from discrete subsets of RG predetermined toward specific fates, whose relative proportions change with time. The employment of a simple stochastic model suggests that the single-MP hypothesis is not compatible with the observed clonal outcomes under our assumptions. In particular, although a single MP with simple proliferation dynamics is a valid model to explain empirical clone size distribution, it fails to recapitulate the frequencies of nearly all clone subtypes in the simulations of both E12- and E14-P30 lineages, producing overall too many WMAs and/or BG. These results suggest that committed progenitors are also present, enabling a tighter regulation of BG and WMA production. For instance, WMA-related dissimilarities might reflect the specific properties of bipotent progenitors that produce both WMAs and interneurons [15]. Discrepancies between simulated and empirical lineages could also depend on layer-specific factors biasing cell fate, such as, for instance, the bipotency of PCLps. Moreover, proliferation dynamics may be more complex than the implemented simple proliferative kinetics. Further investigations will allow to clarify these points.

Despite the prominent diversity of clone types, lineages revealed remarkably consistent architectures. First, HomCs were always smaller in size, suggesting a selective divergence of these lineages from HetCs. Second, HetCs showed an analogous stoichiometry in both E12 and E14 families, with few WMAs and similar higher amounts of both BG and GLAs in triple clones and with BG outnumbering GLAs in double clones. Third, and most interestingly, this general architecture was also reflected within individual HetCs, as revealed in E12-P30 clones, where sister cells were mostly organized in subclones. While WMAs appeared to be less prone to cluster with other astrocyte types, the vast majority of these subclones comprised both BG and GLAs and, in turn, displayed a uniform composition, with BG outnumbering GLAs. Thus, subclones with a recurrent relative number of cortical astrocytes may represent a major unitary module upon which HetCs clones are built. The analogous stoichiometry of E12 and E14 clones and similar layer-specific dynamics of vermian and hemispheric astrogliogenesis, as well as PCLp bipotency in both hemisphere and vermis, strongly suggest that the subclone structures found in E12 clones also apply to E14 HetCs. The presence of subclones also prompts the speculation that they may act as functional units, specifically participating in the regulation of defined microcircuits through synchronous calcium fluctuations, similar to functioning of neighboring astrocytes in both the mouse hippocampus and cerebral cortex [44]. Overall, these results indicate that the behavior of gliogenic progenitors in the cerebellum conforms to a remarkably orderly and coordinated program.

**Fig 10:**
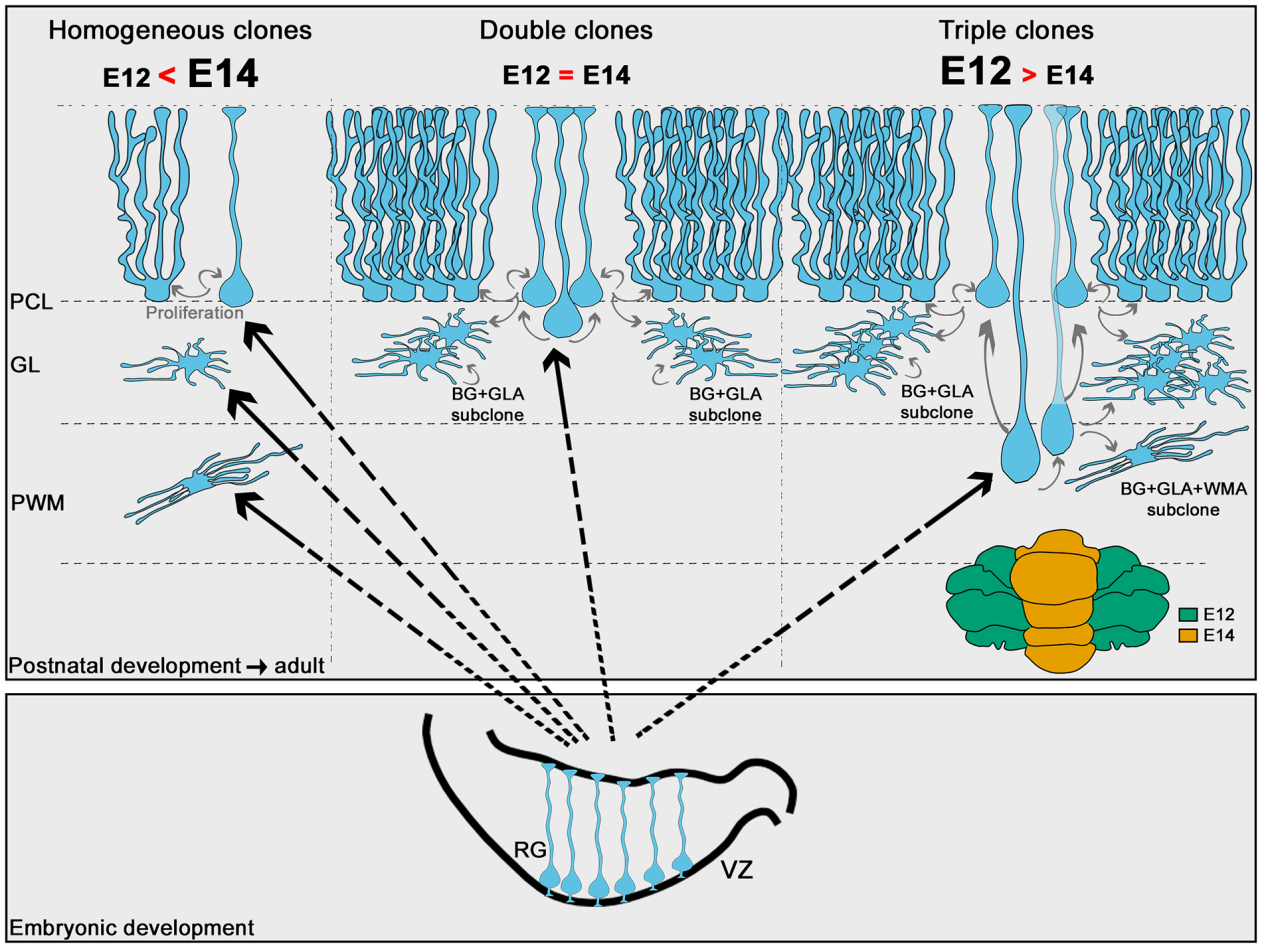
Schematic model for emergence of cerebellar astroglial lineages. Cerebellar astrogliogenesis occurs from RG that either generate HomCs for each major astrocyte type—more frequent among vermian E14-P30 clones—or HetCs. HetCs, including double and triple clones, are proposed to originate from intermediate basal progenitors derived from RG. Double clones are similarly produced by either E12 or E14 RG and appear composed on average of 2 subclones with the same modularity. In these subclones, BG dominates over GLAs, likely as the result of the amplification of ventricular progenitors translocated into the PCL. Triple clones, more frequent among E12-P30 lineages, include the 3 major astrocyte types and appear composed by 3 subclones belonging to distinct typologies, depending on the presence of WMAs. Subclones can be formed by BG+GLA types, in which case they show modularity, and by BG+GLA+WMA types, in which case there is no apparent modularity but a very diverse cell type composition. BG, Bergmann glia; E, embryonic day; GL, granular layer; GLA, granular layer astrocyte; HetC, heterogeneous clone; HomC, homogeneous clone; RG, radial glia; PCL, Purkinje cell layer; PWM, prospective white matter; VZ, ventricular zone; WMA, white matter astrocyte.

How is this stereotyped architecture achieved? Here we identify 2 contributing factors: (i) Distinct layer-dependent rhythms of astrocyte amplification and cell cycle exit, which well fit the clone/subclone architectures. Namely, WMAs leave the cell cycle early while proliferation in cortical layers is more intense and lasts longer, especially in the PCL, in parallel with the tangential expansion of the cerebellar surface. Distinct molecular machineries could sustain different rhythms, as suggested by evidence that PCL and PWM astroglial progenitors rely on different types of cyclins D [15]. Yet, cell-extrinsic factors are likely to modulate these behaviors. Neuron-derived signals are known to affect cerebellar astrocyte proliferation, differentiation, and acquisition of layer-specific phenotypic traits [7,45–47], thereby possibly taking part also in layer-specific dynamics. In particular, Sonic hedgehog (SHH) derived from PCs is able to induce the neurochemical conversion of GLAs into BG [7]. Shh is thus an obvious candidate for a cortical layer–specific cue that can bias the differentiation propensity of PCLps toward BG over GLAs. (ii) PCLps are not exclusively committed to the BG fate and can give rise to clones containing both BG and GLAs. Thus, PCLps appear as the most likely source of double HetCs and BG+GLA containing subclones, but this hypothesis remains to be directly demonstrated. This interpretation is in agreement with findings in mutants where bona fide BG progenitors (here named PCLps) fail to develop, with consequent prominent loss not only of BG but also of GLAs [13,48]. Moreover, the gliogenic plasticity of PCLps fits well the adaptive reprogramming shown for these progenitors after a major depletion of the perinatal external granular cell layer (EGL) [49]. Further, PCLp horizontal cleavage plane orientations establish a similarity to neocortical basal RG [24,50–52], which adds to former evidence of phenotypic [53] and functional (i.e., contribution to folding [13,48,54]) relationships between these 2 basally anchored progenitors. In sum, our results point to PCLp as the fundamental organizing element of cortical components in HetCs. In addition, we speculate that part of these basal progenitors after detachment from the VZ and before reaching the PCL could divide and also seed WMAs/GLAs, thereby identifying them as a key organizer of cerebellar astrogliogenesis (Fig 10).

In summary, these findings reveal that an unprecedented well-defined developmental program structures astrocyte clonal architecture in the cerebellum and suggest that RG subsets with distinct fate potentials contribute to astrogliogenesis. It will be interesting to determine whether similar rules also apply to other CNS areas.

## Methods

### Ethics statement

All procedures were in accordance with the European Communities Council Directive (and 86/609/EEC and 2010/63/EU), the National Institutes of Health guidelines, and the Italian Law for Care and Use of Experimental Animals (DL116/92). They were approved by the Italian Ministry of Health and the Bioethical Committee of the University of Turin (Project “Plasticità strutturale, rigenerazione assonale e sostituzione cellulare per la riparazione del danno nel sistema nervoso” ROSSI/13-16 and Project. 741/2016-P). The study was conducted according to the Arrive guidelines.

Pregnant mice for IUEs were deeply anesthetized by inhalation (1%–2.5% of isoflurane in O2 in a mixture of 30: 70 O2/N2O). Transcardial perfusion was performed under anesthesia (hypothermia up to P7; i.p. injection of a mixture of ketamine, 100 mg/kg, Ketavet, Bayern, Leverkusen, Germany; xylazine, 5 mg/kg; Rompun; Bayer, Milan, Italy for older mice).

### Experimental animals

Experimental procedures were performed on the following mouse lines: C57BL/6, hGFAP–green fluorescent protein [27] (GFP), and GLAST^CreERT2/+^ [55]xR26R^Confetti/+^ [20] mutants. Mice of either sex were included. The day of vaginal plug detection was defined as E0, and the day of birth was considered as P0. Mice were maintained on a 12-h day/night cycle with adequate food and water.

### IUEs

E12 or E14 pregnant mice were deeply anesthetized throughout the surgery duration and placed on a warming blanket. The skin and the muscular abdominal wall were cut, and the uterine horns were exposed. Embryos were constantly maintained moist with physiological saline and were transilluminated with a cold light to hit the proper injection site. Using a picopump (WPI) connected to a glass microcapillary tube, 2 μl of the hGFAP-StarTrack plasmid mixture [4] (2–5 μg/μL of DNA in 0.1% fast green) were injected into the fourth ventricle of each embryo in order to reach the cerebellar VZ and stably mark VZ RGs [17]. After injecting all embryos, the heads were placed between the forceps-shaped electrodes of a BTX electroporator (Holliston, MA, United States), and 1 train of 5 square pulses was applied (30V or 35V for E12 and E14 electroporations, respectively; 50 ms followed by 950-ms intervals). Afterwards, the uterine horns were positioned back into the abdominal cavity, and both muscle and skin were sutured. Pregnant mice recovered under a warm lamp and then were returned to their home cage. After surgery, the analgesic Rimadyl (5 μg/g) was subcutaneously administered to reduce postsurgical pain. Injected embryos were allowed to develop normally and were analyzed 24 h after electroporation or at P0 or P30. Of note, inspections 24 h post electroporation showed that clones were always in only one-half of the cerebellum, similar to other studies [17]. This is likely due to positional constraints of the electrodes that targeted only one of the 2 sides of the cerebellar primordium, which are discrete and independent developmental modules [56,57]; yet, electroporated cells had a rather diffuse distribution in the VZ of each lateral primordium along both the M-L and A-P axes (S1D and S1E Fig).

### Local administration of Tx

To allow Cre recombination exclusively in radial PCLps, we topically administered Tx citrate crystals (Sigma-Aldrich) on the pial surface of the vermis of Confetti mice to selectively target glial endfeet as previously described [15], with modifications. Briefly, mouse pups were anesthetized by hypothermia. After opening the skin and the skull, leaving the pial surface intact, we placed on the cerebellar surface a Tx amount corresponding to about 30–80 μg in order to tag a limited number of PCLps. Finally, we carefully wiped the wound and closed it. The animals were then killed after 48 h for short-term analyses or at P30.

### Thymidine analogues

BrdU (Sigma-Aldrich) and EdU (Invitrogen) were dissolved at 10 mg/ml in sterile saline and administered via i.p. injections in pregnant females and via s.c. injections in neonatal pups. The same dose of 100 mg/kg body weight was used for both analogues. EdU was injected 6 h before killing. For active proliferation analyses in StarTrack-labeled cells, a double-pulse EdU protocol with a 3-h interval was adopted in order to increase the chance of labeling the electroporated cells. Animals were then killed 3 h after the last EdU injection. For BrdU labeling, samples were incubated in 2N HCl for 20 min at 37 °C, washed with borate buffer (pH 8.5) for 10 min, and processed for anti-BrdU antibody staining. EdU was detected using a commercial kit (Click.iT, Life Technologies) after the immunohistochemical reactions.

### Histological and immunohistochemical procedures

Under anesthesia, animals were transcardially perfused with an appropriate volume of 4% paraformaldehyde (PFA) in 0.12 M phosphate buffer (PB), pH 7.2–7.4. Brains were removed, stored overnight (o/n) in the same fixative at 4 °C, washed in PBS, and finally cryoprotected in 30% sucrose in 0.12 M PB. The cerebella were then embedded and frozen over dry ice in OCT (TissueTEK), sectioned in the parasagittal plane at 30 μm using a cryostat and collected in PBS (P30 juvenile cerebella), or placed directly onto glass slides (E13-P7 cerebella). For immunolabeling, sections were incubated o/n at room temperature with the appropriate primary antibodies dissolved in PBS with 1.5% donkey or goat serum (Jackson ImmunoResearch) and 0.25% Triton X-100 (Sigma-Aldrich): anti-GFP (1:700, rabbit, polyclonal; Invitrogen A-11122), anti-GFP (1:700, chicken, polyclonal; Aves Labs GFP-1000), anti-RFP (1:1,500, rabbit, polyclonal, Rockland 600-401-379), anti-BrdU (1:500, rat, monoclonal; Abcam ab6326), anti-GFAP (1:1,500, rabbit, polyclonal; Dako Z0334), anti-pVimentin (1:500; mouse, monoclonal; MBL International D076-3S); anti-Parvalbumin (PV, 1:1,500, mouse, monoclonal; Swant 235); anti-GLAST (1:200, rabbit, polyclonal, Abcam, AB416); anti-Aquaporin 4 (AQP4, 1:2,500, rabbit, polyclonal, Sigma Prestige Antibodies, HPA014784); anti-GDF10 (1:25, rabbit, polyclonal, Sigma Prestige Antibodies, HPA015498); anti-KIR1.4(1:100, rabbit, polyclonal, APC-035, Alomone Labs); anti-SOX10 (1:100, goat, polyclonal, Santa Cruz, SC17342); anti-NeuN (1:500, mouse, monoclonal, Merck Millipore, MAB377); anti-PAX2 (1:200, rabbit, polyclonal, Invitrogen, 71–6000); anti-NFIA (1:1,000, rabbit, polyclonal, Abcam, 41581); anti-BLBP (1:200, rabbit, polyclonal, Merck Millipore, ABN14), anti-Ki67 (1:500, rabbit, polyclonal, Abcam, ab15580). Sections were then exposed for 2 h at room temperature to secondary species-specific antibodies conjugated with Alexa Fluor 488 (1:500; Invitrogen; anti-rabbit A-21206; anti-mouse A-21202; anti-chicken A-11039), Alexa Fluor 555 (1:500; Invitrogen; anti-rabbit A-31572) or Alexa Fluor 647 (1:500; Invitrogen; anti-rabbit A-21244; anti-mouse A-31571), and Cy3 (1:500; Jackson ImmunoResearch; anti-mouse 715-167-003; anti-rat 712-165-153). Cell nuclei were visualized using 4′,6-diamidino-2-phenylindole (DAPI; Fluka). After processing, sections were mounted on microscope slides with Tris-glycerol supplemented with 10% Mowiol (Calbiochem).

For StarTrack clonal analysis, P0 and P30 cerebella were parasagittally cut in 50-μm-thick serial sections. For R26R^Confetti^ stainings, 60-μm-thick serial sections were obtained with a vibrating microtome (Leica). When sections thickness was >30 μm, sections were incubated for 48 h at 4 °C in primary antibody solution containing 1.5% donkey serum and 2% Triton-X100. Secondary antibody incubation was performed o/n in PBS with 1.5% donkey serum and 0.5% Triton-X100. Stainings for Sox10 and Ki67 on thick sections were preceded by antigen retrieval with citrate buffer (pH 6. 20 min at 80 °C followed by 20 min at room temperature).

### Data analysis and image processing

Histological specimens were examined using an E-800 Nikon microscope connected to a color CCD camera or a Leica TCS SP5 confocal microscope. For StarTrack clonal analysis, confocal images were acquired under fixed excitation and absorption conditions for each fluorophore as previously described [4,58]. In some instances, we applied the colocalization analysis provided by the LAS AF software (Leica) or the colocalization plugin provided by NIH ImageJ software [59] to display in white/purple marker coexpression at the pixel level. Adobe Photoshop 6.0 (Adobe Systems) was used to adjust image contrast and assemble the final images. On the basis of confocal images, cells and clone distributions were mapped by either Neurolucida 7.0 (MBF Bioscience) or Reconstruct [60] software that defined spatial coordinates for each cell, subsequently employed to calculate cell distances. Quantitative evaluations (proliferation, birthdating, and cleavage plane orientation analyses) were performed on confocal images by NIH ImageJ software [59]. Three to 6 animals were analyzed for each time point or experimental condition. Measurements were derived from at least 3 sections per animal. For StarTrack and Confetti clonal analyses, all cerebellar sections were analyzed.

### Definition of StarTrack clones and clonal analyses

To evaluate the expression of hGFAP-StarTrack constructs [4], the whole cerebellar territory was analyzed. Every cell was examined for the presence of each fluorophore, and cells with the same combination of fluorescent proteins were defined as part of the same cell cluster. As analysis of lumping error probability (see below section ‘Estimation of lumping errors in StarTrack clones and impact of repeated color combinations’) indicated that these cluster were clones in the vast majority of cases (91% minimum probability), we refer to clusters as clones. Labeled astrocytes were categorized in distinct types according to their morphology and layering (Fig 1A). These identification criteria were validated in P30 clones by staining for the general astroglial marker GFAP (S2 Fig) and for markers enriched or specific of each astrocyte type (S2 Fig and S1 Table). The same pattern of marker expression was confirmed in astrocytes of both HomCs and HetCs. No coexpression of either neuronal or oligodendroglial markers was found in labeled cells, in full agreement with selective labeling of astrocytes (S2 Fig). Positivity for each antigen was tested on 150–200 StarTrack-tagged cells of different samples. The cellular identity of clones exclusively labeled with nuclear fluorophores was also confirmed by anti-GFAP immunostaining (S13 Fig).

For P0 analyses in the still-developing cerebellar territory, we distinguished as layers the PWM and the cortex (Fig 6A) based on differences in cellular density as detected by autofluorescence and validated by DRAQ5 staining. Among labeled cells, we confirmed tagging of astroglial progenitors by colabeling for BLBP or NFIA and absence of coexpression of lineage markers for either oligodendrocytes or interneurons (S6 Fig), which are actively generated at this stage [8,15,19,61]. Expression of each antigen was tested on 150–200 StarTrack-tagged cells across the distinct cerebellar layers.

M-L clone dispersion was estimated based on the number of sections where the examined clone was dispersed. Clones included in one single section were categorized according to the section thickness (50 μm). A-P clone dispersion was estimated qualitatively as clone distribution in adjacent lobules.

In total, we found 573 hGFAP-StarTrack-labeled clones (for the complete list, see S2 Table), comprising 410 fluorescent combinations. Among them, nearly all (99%) appeared with a frequency of less than 8 × 10^−3^, and only 2 marks were present at the highest frequency (2 × 10^−2^). On the whole, frequencies were within the range analyzed by García-Marqués and López-Mascaraque [4], and therefore, all combinations were included.

### Estimation of lumping errors in StarTrack clones and impact of repeated color combinations

To estimate the probability that cells labeled by the same marker actually derive from independent progenitors (lumping error [62]), we examined the frequency of StarTrack color combinations across all cerebella and found some repetitions (S3A Table). Since each color was equimolarly represented in the plasmid mix, we do not expect an overrepresentation of defined colors in the plasmid mix. Yet, different stabilities of individual fluorophores or distinct integration efficiencies of constructs might in principle influence the resulting combinations. We therefore calculated the lumping error probability for all cerebella, following Fuentealba and colleagues [63], based on the number of clones and the repetitions of StarTrack combinations found in the pooled data. A vector was designed with elements (color combinations) repeated according to S3A Table. Thus, repeated combinations had a higher probability to be chosen than the elements representing unique combinations. From the designed vector and using a uniform random experiment, samples were picked that corresponded to the number of elements in each analyzed cerebellum (S3B Table). The picked elements from each sample were compared one-to-one, and the probability of observing one of the repeated combinations was computed. Using a Monte Carlo simulation, this experiment was repeated 300,000 times to characterize the probability density function of these events. Means and standard deviations of probability distributions for each cerebellar sample are shown in S3B Table. Results showed an average global probability of lumping errors of 3%, which varied between 0.6% and 9%, depending on the animal. For the majority of samples, this error was low and comparable to that of bar code–based approaches in which about 10,000 combinations are available [63]. From the observed number of repeated combinations and the number of clones per sample, we conclude that in the vast majority of cases (91%, corresponding to the highest lumping error probability), the cells that share the same StarTrack combination derive from a single progenitor.

Yet, the presence of repeated combinations suggests that these are less reliable to define sibling cells in the samples [58]. With the aim to disclose potential systematic biases introduced by clones defined by the repeated combinations, we examined whether these clones belonged to specific types/subtypes and analyzed their features in the samples with the highest probability of lumping errors (S14 Fig). We found that repeated combinations were homogeneously represented among the distinct clone types/subtypes and had a size (which is a key clonal parameter) similar to clones with unique combinations. This shows that their inclusion in the analysis does not introduce a systematic bias in the results and that the presence of clones labeled by repeated combinations did not significantly affect results (S14 Fig).

### NND and cluster analyses

To assess whether cells were uniformly distributed within the cerebellar tissue or rather formed clusters, we focused on E12-P30 HetCs that were especially numerous and large in size and performed a nearest neighbor analysis followed by a cluster analysis, the former to verify the presence of cell aggregates (subclones) and the latter to estimate the number and size of subclones as well as their composition in terms of cell category types. A significant departure of the actual cumulative distribution of NND from a random distribution was considered a sign of clustering [31]. To this end, for each clone type, we generated a uniformly distributed random sample of spatial coordinates, using the same number of observations and the corresponding cerebellar volume, and the cumulative distribution of the resulting NNDs was computed. For both empirical and randomly generated data, we fitted an asymmetric sigmoidal function (Richard’s model) using the least squares method.

Cluster analysis was performed with the k-means method (100 replicates). A progressively larger number of clusters (from 1 to 5) was tested until 95% of accounted variance was reached. This procedure estimated the actual number of clusters (subclones) in a clone. Only clones with a minimum of 6 cells were considered, and a minimum of 3 cells per cluster was imposed. Although cluster analysis is considered to be more of an exploratory than a confirmatory approach [64], results were confirmed by visual inspection, which also suggested that clustering errors (e.g., cases in which cells are spatially but not anatomically close to each other or cases in which isolated cells are forced into a cluster) remained below 10%. We further estimated the impact of clustering errors on the distribution of subclone types and on the BG:GLA ratio. To this aim, we reran the cluster analysis, introducing a fictitious error in the number of identified subclones (20%; this was achieved empirically by using 90% and 99% accounted variance thresholds). The resulting subclone type distributions were virtually identical, and the resulting ratio change was negligible (0.015 log units on average, S15 Fig and cf. Fig 5E–5H), thus suggesting that the cluster analysis was quite robust to clustering errors.

### Estimation of splitting errors in StarTrack clones

A splitting error occurs when siblings are not recognized as originated from the same progenitor [62]. Our cluster analysis subdivided triple and double StarTrack clones in 2 or more spatially segregated subclones. However, theoretically, these subclones could represent a coincidental appearance of the same color combination in distinct clones (corresponding to the subclones). Following Walsh and Cepko [65] and Kirkwood [66], we computed the probability that any one of the distinct color combinations will appear twice or more by chance in the cerebella analyzed for cluster analysis by using Monte Carlo simulations with 300,000 repetitions. Splitting error probability was computed under the assumption that each of the StarTrack’s 4,096 theoretical combinations [67] has the same probability to be picked out, thus considering a uniform distribution. Since each color was equimolarly represented in the plasmid mix, we do not expect an overrepresentation of some colors over others. Thus, we considered a uniform distribution to avoid giving nearly zero probability to the combinations that were not observed, likely because of the limited number of clones found, so to avoid underestimating the overall number of the potential combinations, which is appropriate for the methodology (StarTrack) adopted. We assumed that any of the theoretical color combinations has the same probability to be picked out in random draws with replacement and that subclones were independent clones. According to the results of cluster analysis, we assumed that on average, each E12 triple clone corresponded in fact to 3 clones and each double clone to 2 clones, for an estimated total of 244 clones in sample 1 and 139 in sample 2. The splitting error probability was computed as the number of repetitions of the same color combination occurred by chance over the total number of estimated clones. In the analyzed samples, it was 3% and 2%, respectively. Thus, the splitting error probability was quite low.

### Definition of Confetti clones

R26R^Confetti^ mice enable the distinction of progenies derived from different single progenitors thanks to the stochastic and exclusive expression of 1 out of 4 fluorescent proteins. In order to visualize different fluorophore combinations, cerebellar sections were stained with anti-GFP antibody that recognizes GFP, YFP, and CFP and with anti-RFP antibody for RFP. Cells marked with GFP were then discriminated according to the localization of the green staining: nuclear (corresponding to GFP; very rare cells, therefore excluded from the analysis), cytoplasmic (corresponding to YFP), or membrane associated (corresponding to CFP), as previously described [68].

In P30 cerebella, tagged cells exclusively belonged to the astrocytic lineage as confirmed by positivity for GFAP and negativity for the oligodendrocyte-specific transcription factor SOX10 and for neuronal markers, as previously described [15] (S8 Fig). Distinct astrocyte types were recognized by morphological and layering criteria that were confirmed by analysis of markers expression (S8 Fig). Cells appeared distributed in small groups dispersed in the cerebellar cortex, suggestive of clones. Putative clones were identified in serial sections of the cerebellum (S4 Table) based on expression of the same color; location in the same lobule and lobular wall (considering the WM as dividing each lobule in 2 walls), as derived from StarTrack analyses; and cell spatial contiguity. Indeed, the very low number of color tags requires additional criteria for clone identification. Lobules IV–V and IX (vermis) and Crus II and I (hemisphere) were analyzed without finding any significant difference (Vermis lobule IX: BG+GLA clones 45.8% ± 8.4%; BG clones 44.8% ± 7.5%; GLA clones 9.4% ± 3.8%; chi test *P* > 0.05. Hem: BG+GLA clones 47% ± 2.5%; BG clones 43% ± 2.6%; GLA clones 6% ± 1.3%; chi test *P* > 0.05; cf. Fig 7). Therefore, for simplicity, only data of lobule IV–V are presented. The adopted criteria for clone identification and the method used to tag a limited number of PCLps were validated by analysis of recombined cells 48 h after Tx administration at P6. At this time point, analyses of lobule IV–V in serial cerebellar sections confirmed tagging of sparse individual cells in the PCL expressing markers of astrocyte progenitors (S7A–S7D’ Fig and S4 Table). The number of these PCLps was compatible with that of P30 putative clones (39.42 ± 6.32 progenitors versus 38.78 ± 3.72 clones per color, respectively; *t* test, *P* = 0.931). At 48 h, we found also pairs of cells (about 20% of all tagged cells) labeled with the same color that were reminiscent of mitotic PCLps (S7E and S7E’ Fig) or, based on mirror morphologies and/or close proximity (approximately 30 μm), of duplets of newly generated sister cells (S7F–S7G’ Fig). Further, in most cases, cells in duplets were positive for the proliferative marker Ki67 (S7E–S7G’ Fig). Overall, these features indicated that these pairs are the initiation of a clone.

We also reasoned that the distance among tagged PCLps shortly after Tx induction would provide a reference to evaluate the accuracy of cell clustering in clones at P30, assuming that cell dispersion is mainly due to developmental growth of the cerebellum. Thus, the distance between each PCLp and the nearest PCLp of the same color (NND, S4 Table) was calculated, averaged, and scaled according to the growth of lobule IV–V from P8 (i.e., P6 + 48 h) to P30 (2.3-fold, as determined by ratios of the geometric means of 3D measurements of the lobule at the 2 time points). We inferred that the ensuing value (137.62 μm × 2.3 = 317 μm) would define the minimal distance between Confetti clones at P30. We found that the minimal clone distance was much higher than the mean clone extension—estimated through either the mean (104 μm, *P* < 0.001) or the maximal (153 μm, *P* < 0.001) intraclone intercell distance (see S7H Fig and S4 Table). Indeed, only a small fraction of clones had an extension close to the minimal clone distance, indicating that clones do not overlap and thus confirming the reliability of the adopted criteria to identify Confetti clones. Moreover, in further support of consistent clustering of cells in clones, the average NND between sister cells composing P30 clones was consistent across different cerebella and different colors (67 ± 3 μm).

### Determination of cleavage plane orientation

Anti-pVimentin staining was used to identify PCLps undergoing mitosis [69]. The cleavage plane orientation was measured by calculating the angle between the equatorial plate of DAPI-stained nuclei and the PCL trajectory. Angle values of 0°–30° were defined as horizontal, 30°–60° as oblique, and 60°–90° as vertical. Only cells in anaphase or telophase were analyzed, in at least 3 different animals per time point (50–86 cells analyzed in total per time point).

### The stochastic simulation algorithm

#### Approach

To investigate the rules that govern the cell fate decision of cerebellar RG, we checked the validity of a model that assumed all astrocyte progenitors as belonging to a homogeneous multipotent population. This was done by generating clones in silico with discrete-time stochastic simulations, using a variant of the Stochastic Simulation algorithm, also known as the Gillespie algorithm [70,71]. The empirical outcomes of 275 clones and 98 clones at E12-P30 and E14-P30 respectively (i.e., clone size, proportions of astrocytes, ratio of astrocyte types per clone, proportions of clone types and subtypes; CNA astrocytes were omitted) were compared to the in silico simulation output (5 replicates of 275 or 98 clones for E12-P30 and E14-P30 simulations respectively).

#### Description of the simulation algorithm

- Step 0:
  - Initialization of the transition probabilities;
  - Initialization of the type of the root cell (E12: 70% MPs, 30% postmitotic astrocytes, E14: 60% MP, 40% postmitotic astrocytes. The proportions correspond to the observed frequency of unicellular clones at P30; within the initial postmitotic astrocytes, the discrete proportions of BG, GLA, and WMA unicellular clones were used to determine the fate of already postmitotic astrocytes at the beginning of the simulated lineages)
- Step 1:
  - Each MP undergoes a division event. Each daughter cell has a certain probability of remaining an MP (i.e., probability of cell cycle reentry, pMP) or becoming a postmitotic astrocyte (i.e., probability of cell cycle exit). Daughter cell fates were determined stochastically, according to the division proliferation/differentiation probabilities.
- Step 2:
  - Each postmitotic astrocyte has a certain probability to become a GLA, BG, or WMA based on the birthdating experiments (i.e., based on the fraction of BrdU^high^/tot BrdU^high^ astrocytes calculated in each layer) and undergoes differentiation stochastically according to the differentiation transition probabilities for the given generation the astrocyte is produced in the lineage (for E12-P30 simulations the hemisphere birthdating results were used, whereas the birthdating results measured in the vermis were taken for E14-P30 simulations);
- Iterate Step 1 and Step 2 until all cells differentiate.

#### Choice of a 1-compartment and discrete-time model

Based on the collected data and following a parsimony principle to limit open parameters, a space-independent model was built, in which the pMP was kept constant regardless of the localization of each MP in the distinct emerging cerebellar layers. A range of pMPs were tried in the simulations (0.4 to 0.5 with a step of 0.005; 5 replicates of 3,000 clones were generated in each case), and the best pMP was defined as the one minimizing the sum of squares of the residuals on the clone size distributions (pMP = 0.465 for E12-P30 clones and pMP = 0.44 for E14-P30 clones). A discrete stochastic simulation was chosen in which each step of the simulation corresponds to a generation in the lineage. Equivalence between discrete and continuous stochastic simulations has been demonstrated [71]. The probabilities that a daughter cell became a postmitotic astrocyte of a specific kind were generation-dependently set on the basis of our birthdating analyses (see Fig 9B and 9H). To determine the correspondence between the number of generation development time, we made use of the E12-P0 and E14-P0 clones. To this aim, the size of the simulated clones at distinct generations was compared with the experimental E12-P0 and E14-P0 clone sizes. This allowed us to determine at which generation the simulated clone size distribution matched the P0-observed clone size distribution (sixth generation with pMP = 0.465 for E12-P30 clones, fourth generation with pMP = 0.44 for E14-P30 clones). Using this reference, we were able to convert the developmental time into number of generations and make use of the birthdating results.

### Statistical analyses

Following Legué and colleagues [25], to account for within-mice correlation and sample size variability, we used generalized estimating equations (GEE) with exchangeable correlation structure for both count data (Poisson distribution with log link function) and continuous variables (normal distribution with identity link function). Pairwise comparisons were analyzed with sequential Bonferroni tests. When necessary, a logarithmic transformation was applied prior to statistical evaluation to reduce skewness and compensate extreme values, as well as to represent symmetrically cell type ratios, in which case means were converted back to original ratios. To compare distributions of NND, the Kolmogorov-Smirnov test for 2 independent samples was used. Chi-squared and Fisher exact tests were used for frequency analyses, depending on sample size. Pearson’s r was used for correlation analyses. Confidence intervals (95%) of the means were computed through bootstrap with the method of bias corrected and accelerated percentile method (*N* = 1,000). Alpha level was set to 0.05. When not otherwise specified, data are expressed as means of values of each clone ± SEM. Analyses were performed using Matlab (Mathworks), GraphPad Prism software (GraphPad Software), or SPSS (IBM). Simulations were performed with the R software [72].

For global evaluations, analyses were applied on all clone types formed from E12, E14, or postnatal progenitors. As specified in the Results, more detailed analyses on clone types and subtypes were only applied on major populations (E12-P30, E14-P30, HomC: WMA, GLA, BG; HetC: BG+GLA+WMA, BG+GLA; E12-P0, E14-P0 HomC: PWM, Cortex; HetC).

Only *P* values ≤ 0.05 are reported in the Figures. Details on number of animals, applied statistical tests, and results are summarized in S5 Table.

## Supporting information

S1 Fig Electroporation of E12 and E14 cerebellar VZ. (A) IUE at E12 (StarTrack cytoplasmic eGFP plasmid) and E14 (whole StarTrack mixture) are performed to target the cerebellar VZ. (B,C) Analyses of embryos performed 24 h after both E12 (B) and E14 (C) IUE confirm targeting of the VZ. Arrows point to progenitors tagged at E12 that appear to be delaminating at E13. Asterisks point to progenitors still located in the VZ. (D-E) Representative distributions of E12- (green) or E14-targeted (orange) ventricular RG along the M-L (D) or A-P (E) axes derived from 3 embryos. Only one-half of the whole cerebellar anlage is hit at both time points, and cells are spread mediolaterally and along the A-P axis in partly overlapping territories. Each dot represents a pool of cells found in proximate positions; based on the cerebellar symmetry around the midline, all cells were projected on the same half cerebellar primordium. Scale bars: 30 μm. A-P, antero-posterior; D-V, dorso-ventral; E, embryonic day; eGFP, enhanced green fluorescent protein; IUE, in utero electroporation; M-L, medio-lateral; RG, radial glia; VZ, ventricular zone.
(TIF)

S2 Fig Expression of lineage and astrocyte type–specific markers in P30 StarTrack-labeled cells. (A-C) GFAP staining confirms that the StarTrack-labeled cells observed at P30 in the WM (A,A’), in the PCL (B,B’), and in the GL (C,C’) are astrocytes. Reslices of single-step images in A’ show that StarTrack GFP and GFAP colocalize (white color) in sister cells found in the WM. Insets in B’ show colocalization (white color) of StarTrack cytoplasmic GFP and GFAP in BG processes. (D-H) Distinct expression levels of GLAST, GDF10, AQP4, and KIR4.1 are found in StarTrack-labeled astrocytes, in line with different patterns formerly reported for BG and astrocytes of the GL ([44] see also S1 Table). GLAST (D-D”) is enriched in BG and GDF10 (E-E’) is BG specific. AQP4 (F-F”) is expressed by GLA (F’) but not in BG (F”). D’’’ and F’’’ show that cells of HomCs display the same expression pattern found in HetCs. KIR4.1 (G-H”) is enriched in both BG (H’) and GLAs (H”) compared to WMAs (white arrowhead in G,G’), where KIR4.1 levels are negligible. (I-L) Neuronal markers are not expressed in StarTrack-labeled cells. (I,J) Absence of anti-PV staining shows that StarTrack-labeled cells (white arrowheads) are neither molecular layer interneurons (red arrowheads) nor Purkinje cells (white asterisks) [73]. (K,L) Electroporated cells found in the GL (white arrowheads) do not express either the granule cell marker NeuN (K,K’) [74] or the Golgi cell–specific marker PAX2 (M-N’) [75]. (L,L’) No coexpression of SOX10 was found, thereby excluding that tagged cells belong to the oligodendroglial lineage [18]. Scale bars: 30 μm. AQP4, aquaporin 4; BG, Bergmann glia; GDF10, growth differentiation factor 10; GFAP, glial fibrillary acidic protein; GFP, green fluorescent protein; GL, granular layer; GLA, granular layer astrocyte; GLAST, glutamate aspartate transporter; HetC, heterogeneous clone; HomC, homogeneous clone; KIR4.1, Inward Rectifier K+ Channel 4.1; NeuN, neuronal nuclei; P, postnatal day; PAX2, paired box gene 2; PCL, Purkinje cell layer; PV, parvalbumin; SOX10, SRY-box 10; WM, white matter; WMA, white matter astrocyte.
(TIF)

S3 Fig Distribution of E12- and E14-generated clones along the A-P axis. (A,B) The distribution along the A-P axis is plotted as frequency (%) of E12-P30 (A, green) or E14-P30 (B, orange) clones in the lobules of the hemisphere or vermis, respectively. When clones are found in >1 lobule, they are repeatedly counted in each corresponding folium. E12-generated clones are broadly distributed in all lobules of the hemispheres, whereas families deriving from E14 progenitors preferentially allocate in the most anterior and posterior lobules of the vermis. *n* = number of clones. The numerical data used in the figure are included in S1 Data. A-P, antero-posterior; Cp, copula pyramidis; E, embryonic day; Pm, paramedian.
(TIF)

S4 Fig Contribution of HomCs and HetCs to the total number of each astroglial type. About 90% of both E12- (A) and E14-derived (B) BG and GLAs are part of HetCs. On the other hand, WMAs are mostly included in HetCs in E12-P30 clones (A) or HomCs in E14-P30 clones (B). *n* = number of cells. The numerical data used in the figure are included in S1 Data. BG, Bergmann glia; E, embryonic day; GLA, granular layer astrocyte; HetC, heterogeneous clone; HomC, homogeneous clone; WMA, white matter astrocyte.
(TIF)

S5 Fig Analyses of HomC size. (A,B) The frequency distribution of the size of E12-P30 (A, green) and E14-P30 (B, orange) HomCs shows that for the vast majority, they are formed by ≤2 cells. Namely, in both data sets, WMA HomCs are the smallest. (C-F) A relevant amount of E12-P30 (C) and E14-P30 (D) HomCs in all cerebellar layers are composed of only 1 cell. (E) and (F) show the proportion of single cell clones in each layer after E12 and E14 IUE, respectively. More than half of WMA and GLA HomCs are found as single cells, whereas individual clones among BG HomCs are less frequent (Fisher’s exact test shows a statistically significant difference between WM and BG in E12-P30 clones; *, *P* = 0.0265). *n* = number of clones. The numerical data used in panels (A,B,E,F) are included in S1 Data. BG, Bergmann glia; HomC, homogeneous clone; E, embryonic day; GLA, granular layer astrocyte; IUE, in utero electroporation; WM, white matter; WMA, white matter astrocyte.
(TIF)

S6 Fig Expression of lineage markers in StarTrack-labeled cells at P0. (A,B) At P0, StarTrack-labeled astrocytes found in both the cerebellar cortex (A) and PWM (B) express the astrocyte progenitor marker BLBP [8] (the white color in A’ and B’ indicates the colocalization at the pixel level between StarTrack GFP and BLBP). (C,C’) In the PWM, electroporated cells also express NFIA [19], further confirming their identity as astrocyte progenitors. (D,E) In parallel, absence of PAX2 (D,D’) and SOX10 (E,E’) staining in StarTrack-labeled progenitors exclude that they belong to the interneuron or oligodendroglial lineage, respectively [15,18]. Scale bars: 30 μm. BLBP, brain lipid–binding protein; GFP, green fluorescent protein; GL, granular layer; NFIA, nuclear factor 1 A; P, postnatal day; PAX2, paired box gene 2; PCL, Purkinje cell layer; PWM, prospective white matter; SOX10, SRY-box 10.
(TIF)

S7 Fig Short-term analysis of Confetti-labeled cells and comparisons with P30 data. (A) Low magnification of lobule IV–V 48 h after local administration of Tx at P6. Arrows point to sparse PCLps labeled with different Confetti colors (RFP, red arrowheads; YFP, green-filled arrowheads; CFP, white arrowhead with green contour). The position of the cell body in the PCL, the radial morphology and the expression of the astroglial marker BLBP (B-C’) confirm that cells tagged by Tx are PCLps. As expected, PCLps are negative for the oligodendroglial marker SOX10 (D, D’). (E-G’) At short term, the vast majority of the cells (about 80%) are single PCLp, but some pairs of sister cells are also visible. They are composed of 2 juxtaposed cells of the same color. In some cases, they are about to complete a mitosis (nuclei in late telophase in E and relative inset), or splitting apart (F,F’). In other cases, both cells are still in the cell cycle; as assessed by Ki67 expression, they display similar configuration of the nuclei and a mirror morphology, elements indicative of cell division (G, G’ and relative insets). Yellow dotted lines in the insets in E, F, G highlight Ki67^+^ nuclei of duplets. One of the 2 cells in pairs always displays a PCLp feature, while the other one, in some cases, seems to extend stellate-like processes, suggestive of a GLA fate (arrowheads in F’ and G’). The histogram in H represents the distribution of the mean extensions of clones identified at P30 (single-cell clones were excluded; bin size = 15 μm). The estimated minimal distance between clones (317 μm, red dotted line) is significantly higher than the mean clone extension (104 μm, black dotted line; *P* < 0.001), and only a small fraction of clones have an extension close to the minimal interclone distance (see Methods). Scale bars 20 μm and 100 μm in A. The numerical data used in panel (H) are included in S1 Data. BLBP, brain lipid–binding protein; CFP, cyan fluorescent protein; GL, granular layer; P, postnatal day; PCL, Purkinje cell layer; PCLp, Purkinje cell layer precursor; RFP, red fluorescent protein; SOX10, SRY-box 10; Tx, tamoxifen; YFP, yellow fluorescent protein.
(TIF)

S8 Fig Expression of lineage and astrocyte type–specific markers in P30 Confetti-labeled cells. (A,B) Cells labeled after in situ Tx administration in Confetti mice are GFAP^+^ astrocytes in the PCL (A1 and B1,2) or in the GL (A2). (C-F) The morphological and spatial criteria used to identify BG or GLA are validated by the expression of astrocyte type–specific markers [7,76] (see S1 Table). BG (C-C’), but not GLAs (D-D’), express high levels of GLAST and are also positive for the BG-specific marker GDF10 (E-E’). Asterisks in C’ highlight the cell body of GLAST-negative Purkinje cells surrounded by GLAST-positive BG processes. (F-F”) On the contrary, AQP4 stains exclusively astrocytes in the GL (F2,3) but not BG (F1). Both BG (G-G’) and GLAs (H-H’) do not express the oligodendrocyte marker Sox10, confirming their astrocytic identity. A’-A”, B’-B”, C’-D’,E’,F’, F” are single-step confocal images that more clearly demonstrate the specificity of the different stainings in Confetti-positive or negative (G’-H’) cells. Arrowheads point to Confetti^+^ cells. Scale bars: 20 μm. AQP4, aquaporin 4; BG, Bergmann cell; GDF10, growth differentiation factor 10; GL, granular layer; GLA, granular layer astrocyte; GLAST, glutamate aspartate transporter; P, postnatal day; PCL, Purkinje cell layer; SOX10, SRY-box 10; Tx, tamoxifen; WM, white matter.
(TIF)

S9 Fig Cleavage plane orientation of PCLps. (A) Frequency distribution of the cleavage plane orientations in pVimentin^+^ PCLp at different time points. PCLps preferentially divide with a cleavage plane horizontal to the PCL throughout cerebellar development (***, *P <* 0.001). No statistically significant differences are found in the distribution of cleavage plane orientations over time (*P* = 0.107, main effect of time). (B) During late embryonic phases, radial progenitors delaminate from the VZ and start to colonize the developing PCL and keep dividing through horizontal divisions. (C-D’) After birth, proliferating PCLps still maintain a horizontal cleavage plane, independently of the position of the radial process (highlighted by yellow arrowheads). (D’) Magnification of a single confocal plane of the pVimentin^+^ cell in D to show the cleavage plane orientation of the nucleus during telophase. *P* values are computed with GEE analysis. Scale bars: 20 μm (B-D), 10 μm (D’). The numerical data used in panel (A) are included in S1 Data. EGL, external granular layer; GEE, generalized estimated equations; PCL, Purkinje cell layer; PCLp, Purkinje cell layer precursor; pVimentin, phosphorylated Vimentin; VZ, ventricular zone.
(TIF)

S10 Fig Active proliferation of StarTrack-labeled astrocyte progenitors. Analysis of active proliferation was performed on E12 hemispheric (green) or E14 vermian (orange) StarTrack-tagged cells in different layers during early postnatal development. Mice were administered twice with EdU with a 3-h interval, and the percentage of EdU-incorporating astrocyte precursors over the total amount of StarTrack-labeled cells in each layer was calculated. At both P1 (A) and P4 (B), the tagged progenitors show a layer-specific pattern of proliferation that declines over time. E12-tagged (green) astrocyte progenitors in the PCL show a slightly higher proliferation activity at P1 (A) compared to those electroporated at E14 (orange). On the other hand, at P4 (B), E12-tagged progenitors in the PWM and GL show a trend to be more proliferative compared to their E14-tagged counterparts, although the low number of cells does not allow to reveal a statistical significance. *, *P* < 0.05; **, *P* < 0.01; ***, *P* < 0.001; *P* values are calculated with Fisher’s exact test. *n* = number cells. The numerical data used in the figure are included in S1 Data. E, embryonic day; EdU, 5-ethynyl-2′-deoxyuridine; GL, granular layer; P, postnatal day; PCL, Purkinje cell layer; PWM, prospective white matter.
(TIF)

S11 Fig Proliferation dynamics of postnatal astroglial progenitors. (A) Experimental design: BrdU was injected at P1 or P4 in hGFAP-GFP mice and EdU 6 h before killing at P4 or P7. Triple-labeled cells analyzed in the vermis were plotted as the percentage of total BrdU^+^/hGFAP^+^ cells per layer. At each time point, PCLps reenter more frequently in the cell cycle compared to astrocyte precursors in other layers. In the PWM from P4 on, there is a significant drop in the proportion of astrocytes performing another division. The same trend is also present, though less evident, in the GL. (B-D’’’) Images represent sagittal sections of cerebella at the different time points analyzed after double thymidine analogue labeling: (B-B’’’) P1–P4, (C-C’’’) P4–P7, and (D-D’’’) P1–P7. Merged and single channels for BrdU (red), EdU (blue), and GFP (hGFAP, green) stainings are presented. Arrowheads point to some triple-labeled cells. (E-G) Representative images of P30 cerebella of mice injected with BrdU at the beginning (E15, E) and the end (P15, F,G) of astroglial development. Full and empty arrowheads indicate cells with BrdU^high^ or BrdU^low^ positivity, respectively. Plots represent data averaged from distinct animals. *, *P* < 0.05; ***, *P* < 0.001, calculated with GEE analysis. Scale bars: 30 μm. The numerical data used in panel (A) are included in S1 Data. BrdU, bromodeoxyuridine; EdU, 5-ethynyl-2′-deoxyuridine; GEE, generalized estimated equations; GFP, green fluorescent protein; GL, granular layer; hGFAP, human glial fibrillary acidic protein; P, postnatal day; PCL, Purkinje cell layer; PCLp, Purkinje cell layer precursor; PWM, prospective white matter; WM, white matter.
(TIF)

S12 Fig Simulations of E14 lineages. The probabilities for a differentiating progenitor of generating the distinct astrocyte subtypes (BG versus GLA versus WMA) are generation-dependently set according to the birthdating experiments performed in the vermis, as shown in (A). Histograms in (B-D) show the outcomes of the simulated lineages compared to the experimental data. Simulated clone sizes (B) appear quite similar to those of the observed clones. On the other hand, the model fails to recapitulate the proportions of astrocyte subtypes, with the production of too many BG (C; same color code as in A). Similarly, the model fails with the proportions of clone subtypes (D). (E,F) Simulated and observed lineages were compared at P0 (corresponding to generation 4). Too many HetCs (E) are simulated compared to empirical clones, because of the generation of too many PWM+cortical clones at the expenses of either cortical and PWM families (F). ***, *P* < 0.001, *P* values were calculated with chi-squared test. Cortical clones comprise PCLp HomCs, GLAp HomCs and PCLp+GLAp HetCs; Cortical+PWM clones comprise PCLp+PWM, GLAp+PWM, and PCLp+GLAp+PWM HetCs. The numerical data used in panels (A-F) are included in S1 Data. BG, Bergmann glia; E, embryonic day; GLA, granular layer astrocyte; GLAp, granular layer astrocyte precursor; HetC, heterogeneous clone; HomC, homogeneous clone; P, postnatal day; PCLp, Purkinje cell layer precursor; PWM, prospective white matter; WMA, white matter astrocyte.
(TIF)

S13 Fig GFAP immunostaining confirms the astrocytic identity of labeled cells. (A,B) Reslices of single step images of P30 clones after anti-GFAP staining (white/purple) unequivocally show the astrocytic identity of cells labeled solely with nuclear markers. Scale bars: 30 μm. GFAP, glial fibrillar acidic protein; P, postnatal day.
(TIF)

S14 Fig Impact of clones defined by repeated combinations on features of E12 HetCs with the highest lumping errors. The repeated frequency of some combinations suggests that these may be less reliable to define sibling cells in our samples. Therefore, we assessed whether the clones defined by these repeated combinations in the samples found more prone to lumping errors (2 samples of E12 clones that underwent cluster analysis) belonged to specific clone types/subtypes and displayed features introducing a systematic bias in the analyses. (A) Repeated combinations were homogeneously represented among the distinct clone types/subtypes, with the only exception of BG+GLA+WMA+CNA clones, in which they were enriched (chi test, *P* = 0.025). However, this clone type, being very rare, was not included in quantitative analyses. (B) Clones defined by repeated combinations (blue bars) were not different from the whole populations (black bars) and behaved as clones with unique combinations (red bars) in terms of clone size (*P* > 0.05), which is a key clone feature. These results suggest that the presence in the examined samples of clones labeled by repeated combinations does not significantly affect results. The numerical data used in panel (B) are included in S1 Data. E, embryonic day; HetC, heterogeneous clone.
(TIF)

S15 Fig Impact of potential clustering errors. Because of the possible presence of a nonnegligible clustering error, we estimated the impact of a subclone identification bias by rerunning the cluster analysis varying by 20% the number of identified subclones (this was empirically achieved by using 90% and 99% accounted variance thresholds). The ensuing changes in subclone type distributions and ratio were negligible (cf. Fig 5E–5H). The numerical data used in the figure are included in S1 Data.
(TIF)

S1 Table Heterogeneity in major astrocyte types. (DOCX)

S2 Table List of StarTrack clones and measured parameters. (XLSX)

S3 Table Repeated combinations and probabilities of lumping errors. (DOCX)

S4 Table List of Confetti clones, measured parameters, and short-term analysis. (XLSX)

S5 Table Statistical analyses. (XLSX)

S1 Data Excel spreadsheet containing, in separate sheets, the underlying numerical data of Figs 3A, 3B, 3C, 3D, 4A, 4B, 4C, 4D, 5A, 5E, 5F, 5G, 5H, 6D, 6E, 7G, 7H, 8A, 8E and 9B, 9C, 9D, 9E, 9F, 9G, S3A, S3B, S4A, S4B, S5A, S5B, S5E, S5F, S7H, S9A, S10A, S10B, S11A, S12A S12B, S12C, S12D, S12E, S12F, S14B and S15. (XLSX)

## Abbreviations

A-P: antero-posterior
BG: Bergmann glia
BLBP: brain lipid–binding protein
BrdU: bromodeoxydine
CNA: cerebellar nuclei astrocyte
D-V: dorso-ventral
E: embryonic day
EdU: 5-ethynyl-2′-deoxyuridine
EGL: external granular cell layer
GEE: generalized estimating equations
GL: granular layer
GLA: granular layer astrocyte
GLAp: granular layer astrocyte precursor
GLAST: glutamate aspartate transporter
HetC: heterogeneous clone
hGFAP: human glial fibrillary acidic protein
HomC: homogeneous clone
IUE: in utero electroporation
mCerulean: monomeric Cerulean
mCherry: monomeric Cherry
mKO: mKusabira Orange
M-L: medio-lateral
MP: multipotent progenitor
mT-Sapphire: monomeric T-Sapphire
NeuN: neuronal nuclei
NFIA: nuclear factor 1 A
NND: Nearest Neighbor Distance
P: postnatal day
PAX2: paired box gene 2
PC: Purkinje cell
PCL: Purkinje cell layer
PCLp: Purkinje cell layer precursor
Pm: paramedian
pVimentin: phosphorylated Vimentin
PWM: prospective white matter
RG: radial glia
SHH: Sonic hedgehog
SOX10: SRY-box 10
Tx: tamoxifen
VC: ventricular ce
VZ: ventricular zone
WMA: white matter astrocyte
