# Supplementary figures and images for: Multiple origins and modularity in the spatiotemporal emergence of cerebellar astrocyte heterogeneity
Source: PLoS Biol. 2018 Sep 27;16(9):e2005513. doi: 10.1371/journal.pbio.2005513 (PMC6178385; doi:10.1371/journal.pbio.2005513)

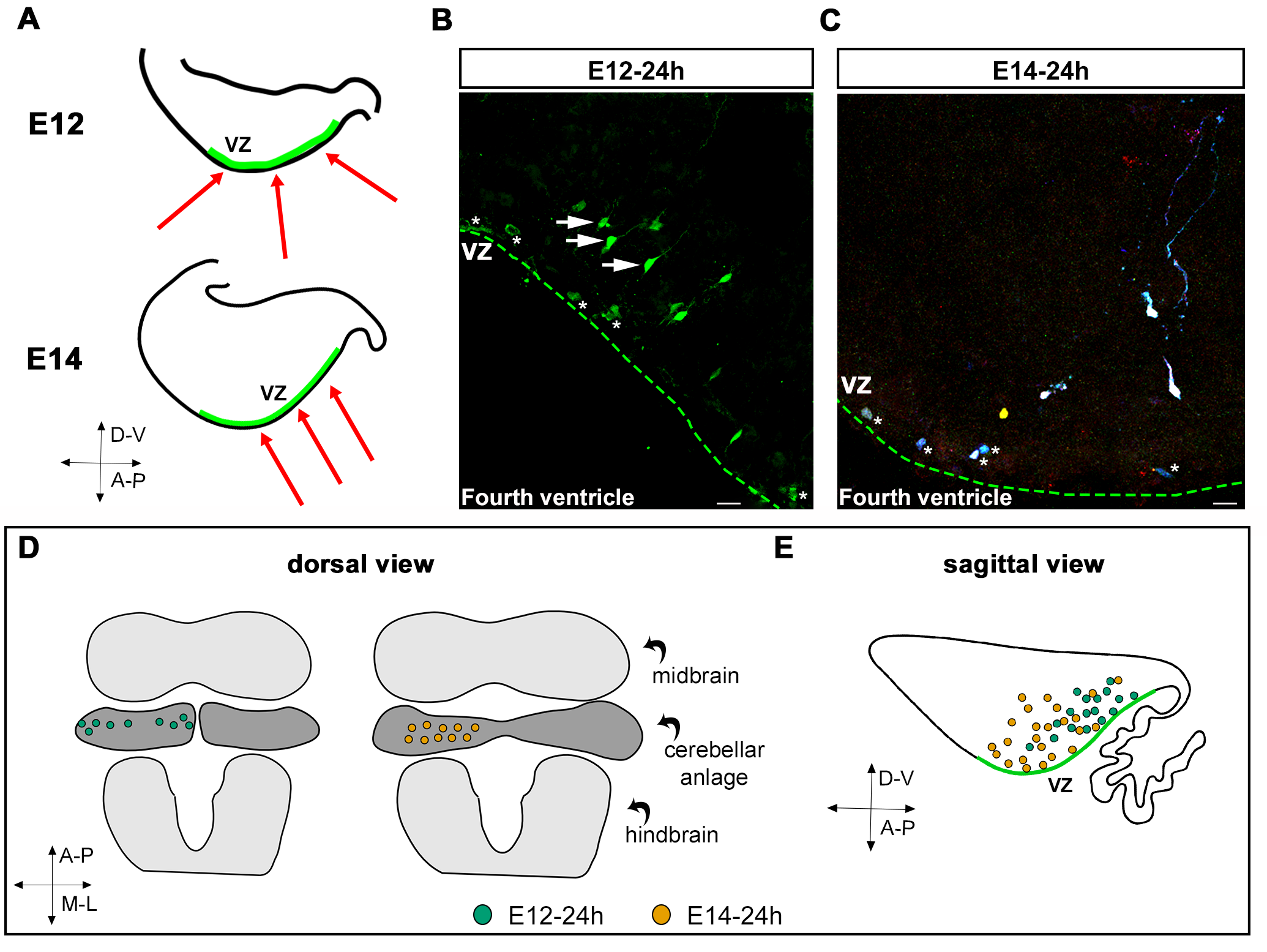

Supplement: S1 Fig — (A) IUE at E12 (StarTrack cytoplasmic eGFP plasmid) and E14 (whole StarTrack mixture) are performed to target the cerebellar VZ. (B,C) Analyses of embryos performed 24 h after both E12 (B) and E14 (C) IUE confirm targeting of the VZ. Arrows point to progenitors tagged at E12 that appear to be delaminating at E13. Asterisks point to progenitors still located in the VZ. (D-E) Representative distributions of E12- (green) or E14-targeted (orange) ventricular RG along the M-L (D) or A-P (E) axes derived from 3 embryos. Only one-half of the whole cerebellar anlage is hit at both time points, and cells are spread mediolaterally and along the A-P axis in partly overlapping territories. Each dot represents a pool of cells found in proximate positions; based on the cerebellar symmetry around the midline, all cells were projected on the same half cerebellar primordium. Scale bars: 30 μm. A-P, antero-posterior; D-V, dorso-ventral; E, embryonic day; eGFP, enhanced green fluorescent protein; IUE, in utero electroporation; M-L, medio-lateral; RG, radial glia; VZ, ventricular zone. (TIF) [file pbio.2005513.s001.tif]

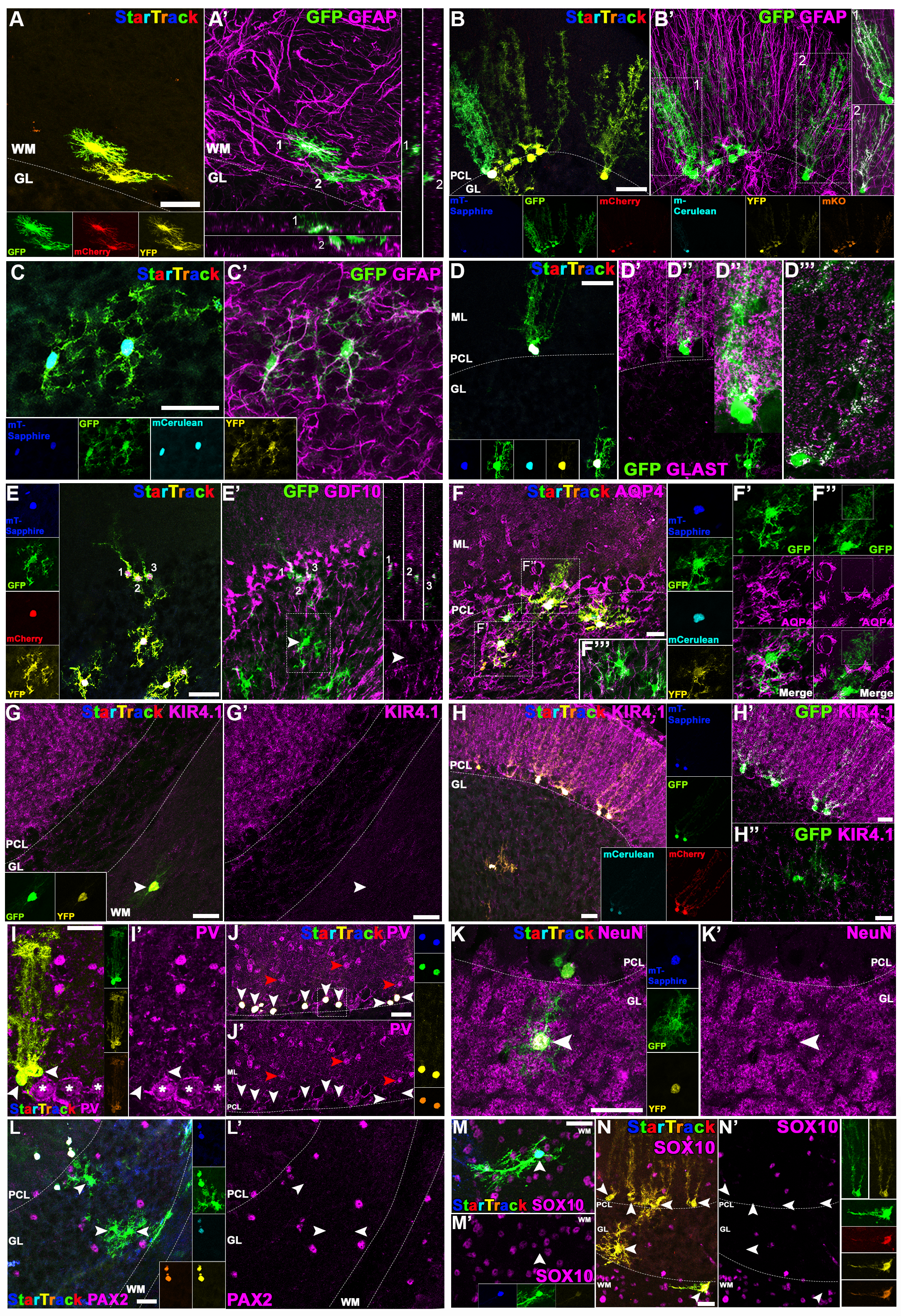

Supplement: S2 Fig — (A-C) GFAP staining confirms that the StarTrack-labeled cells observed at P30 in the WM (A,A’), in the PCL (B,B’), and in the GL (C,C’) are astrocytes. Reslices of single-step images in A’ show that StarTrack GFP and GFAP colocalize (white color) in sister cells found in the WM. Insets in B’ show colocalization (white color) of StarTrack cytoplasmic GFP and GFAP in BG processes. (D-H) Distinct expression levels of GLAST, GDF10, AQP4, and KIR4.1 are found in StarTrack-labeled astrocytes, in line with different patterns formerly reported for BG and astrocytes of the GL ([44] see also S1 Table). GLAST (D-D”) is enriched in BG and GDF10 (E-E’) is BG specific. AQP4 (F-F”) is expressed by GLA (F’) but not in BG (F”). D’’’ and F’’’ show that cells of HomCs display the same expression pattern found in HetCs. KIR4.1 (G-H”) is enriched in both BG (H’) and GLAs (H”) compared to WMAs (white arrowhead in G,G’), where KIR4.1 levels are negligible. (I-L) Neuronal markers are not expressed in StarTrack-labeled cells. (I,J) Absence of anti-PV staining shows that StarTrack-labeled cells (white arrowheads) are neither molecular layer interneurons (red arrowheads) nor Purkinje cells (white asterisks) [73]. (K,L) Electroporated cells found in the GL (white arrowheads) do not express either the granule cell marker NeuN (K,K’) [74] or the Golgi cell–specific marker PAX2 (M-N’) [75]. (L,L’) No coexpression of SOX10 was found, thereby excluding that tagged cells belong to the oligodendroglial lineage [18]. Scale bars: 30 μm. AQP4, aquaporin 4; BG, Bergmann glia; GDF10, growth differentiation factor 10; GFAP, glial fibrillary acidic protein; GFP, green fluorescent protein; GL, granular layer; GLA, granular layer astrocyte; GLAST, glutamate aspartate transporter; HetC, heterogeneous clone; HomC, homogeneous clone; KIR4.1, Inward Rectifier K+ Channel 4.1; NeuN, neuronal nuclei; P, postnatal day; PAX2, paired box gene 2; PCL, Purkinje cell layer; PV, parvalbumin; SOX10, SRY-box 10; WM, white ma [file pbio.2005513.s002.tif]

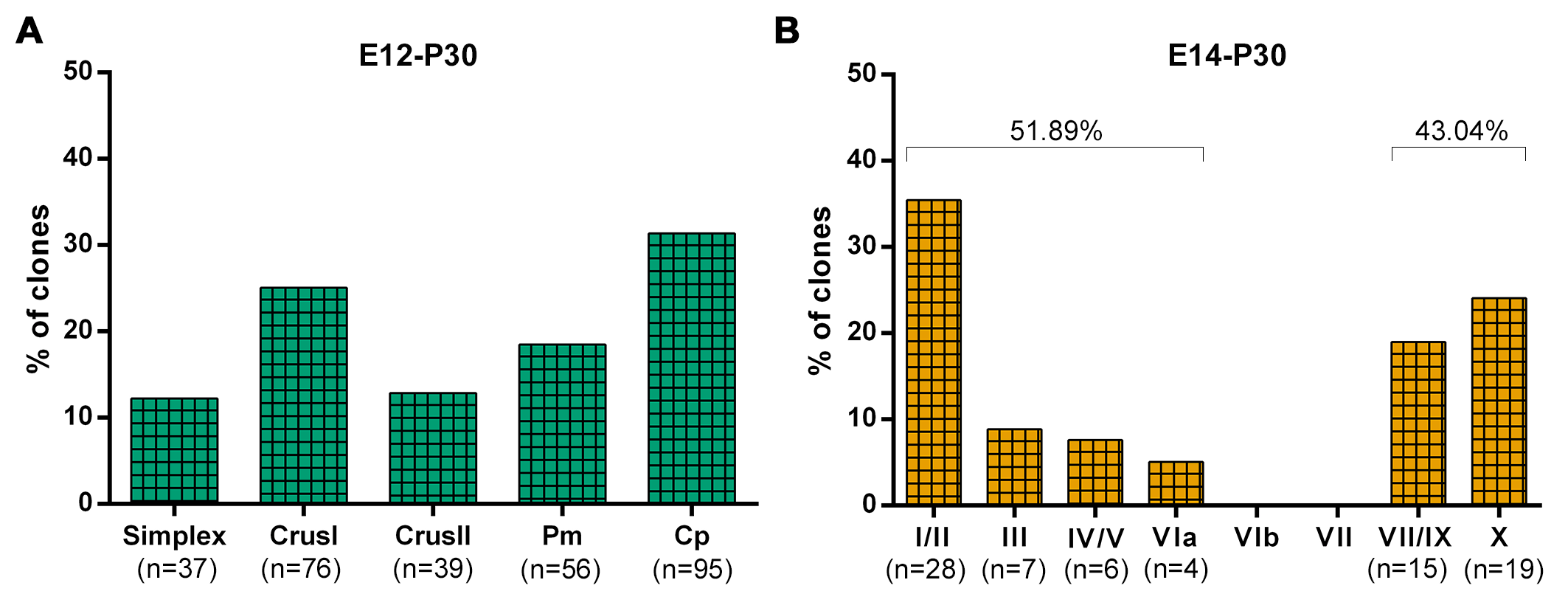

Supplement: S3 Fig — (A,B) The distribution along the A-P axis is plotted as frequency (%) of E12-P30 (A, green) or E14-P30 (B, orange) clones in the lobules of the hemisphere or vermis, respectively. When clones are found in >1 lobule, they are repeatedly counted in each corresponding folium. E12-generated clones are broadly distributed in all lobules of the hemispheres, whereas families deriving from E14 progenitors preferentially allocate in the most anterior and posterior lobules of the vermis. n = number of clones. The numerical data used in the figure are included in S1 Data. A-P, antero-posterior; Cp, copula pyramidis; E, embryonic day; Pm, paramedian. (TIF) [file pbio.2005513.s003.tif]

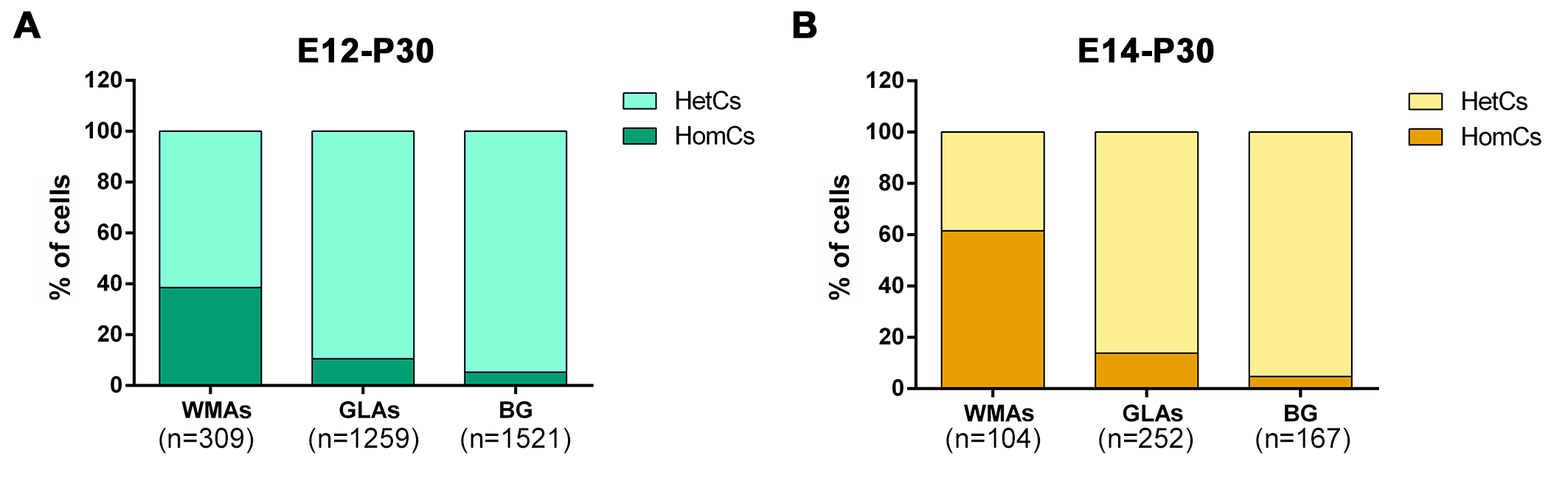

Supplement: S4 Fig — About 90% of both E12- (A) and E14-derived (B) BG and GLAs are part of HetCs. On the other hand, WMAs are mostly included in HetCs in E12-P30 clones (A) or HomCs in E14-P30 clones (B). n = number of cells. The numerical data used in the figure are included in S1 Data. BG, Bergmann glia; E, embryonic day; GLA, granular layer astrocyte; HetC, heterogeneous clone; HomC, homogeneous clone; WMA, white matter astrocyte. (TIF) [file pbio.2005513.s004.tif]

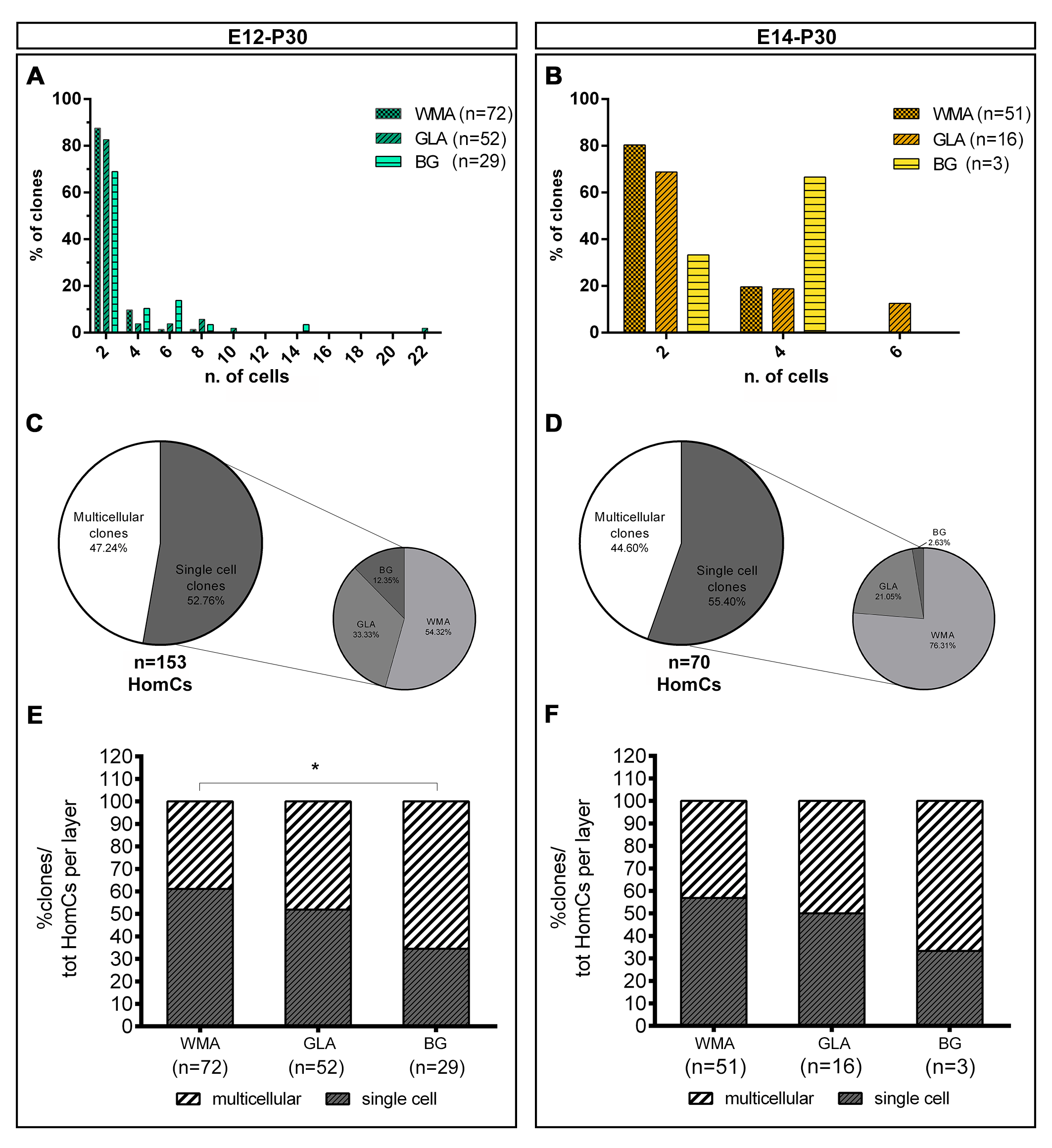

Supplement: S5 Fig — (A,B) The frequency distribution of the size of E12-P30 (A, green) and E14-P30 (B, orange) HomCs shows that for the vast majority, they are formed by ≤2 cells. Namely, in both data sets, WMA HomCs are the smallest. (C-F) A relevant amount of E12-P30 (C) and E14-P30 (D) HomCs in all cerebellar layers are composed of only 1 cell. (E) and (F) show the proportion of single cell clones in each layer after E12 and E14 IUE, respectively. More than half of WMA and GLA HomCs are found as single cells, whereas individual clones among BG HomCs are less frequent (Fisher’s exact test shows a statistically significant difference between WM and BG in E12-P30 clones; *, P = 0.0265). n = number of clones. The numerical data used in panels (A,B,E,F) are included in S1 Data. BG, Bergmann glia; HomC, homogeneous clone; E, embryonic day; GLA, granular layer astrocyte; IUE, in utero electroporation; WM, white matter; WMA, white matter astrocyte. (TIF) [file pbio.2005513.s005.tif]

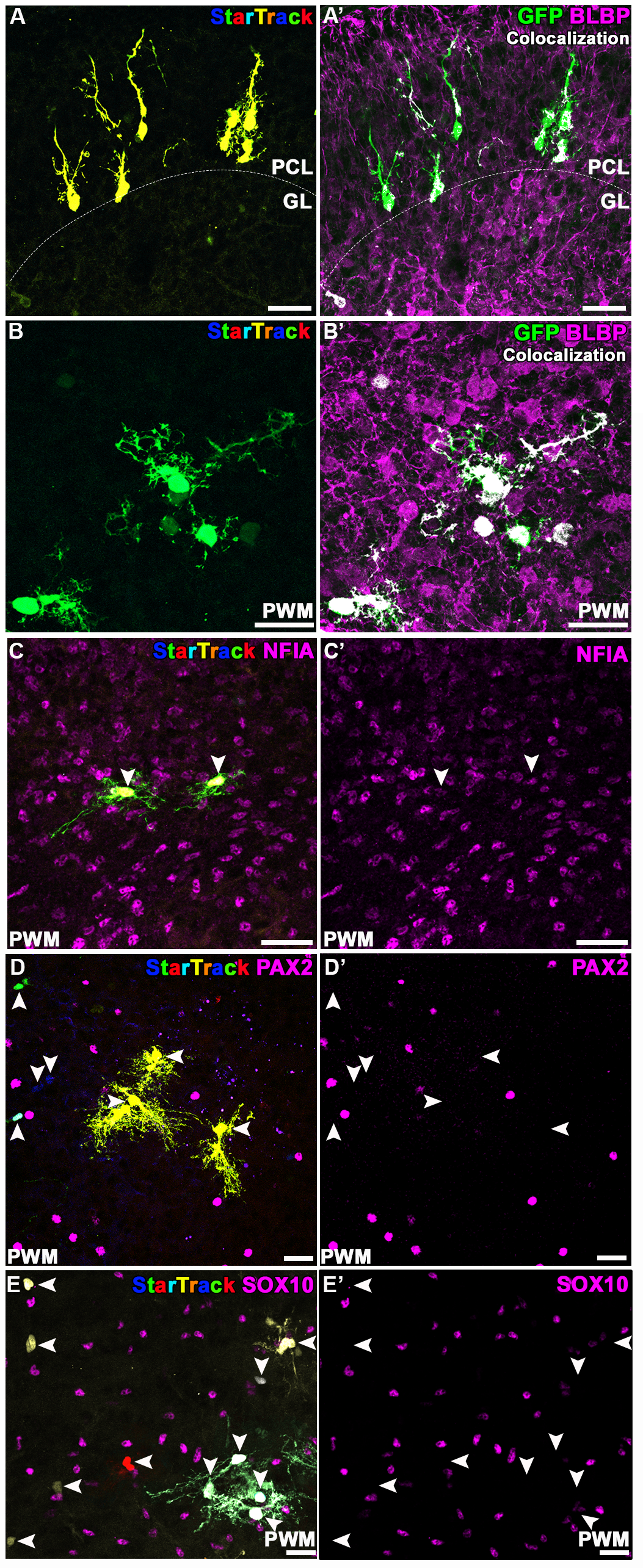

Supplement: S6 Fig — (A,B) At P0, StarTrack-labeled astrocytes found in both the cerebellar cortex (A) and PWM (B) express the astrocyte progenitor marker BLBP [8] (the white color in A’ and B’ indicates the colocalization at the pixel level between StarTrack GFP and BLBP). (C,C’) In the PWM, electroporated cells also express NFIA [19], further confirming their identity as astrocyte progenitors. (D,E) In parallel, absence of PAX2 (D,D’) and SOX10 (E,E’) staining in StarTrack-labeled progenitors exclude that they belong to the interneuron or oligodendroglial lineage, respectively [15,18]. Scale bars: 30 μm. BLBP, brain lipid–binding protein; GFP, green fluorescent protein; GL, granular layer; NFIA, nuclear factor 1 A; P, postnatal day; PAX2, paired box gene 2; PCL, Purkinje cell layer; PWM, prospective white matter; SOX10, SRY-box 10. (TIF) [file pbio.2005513.s006.tif]

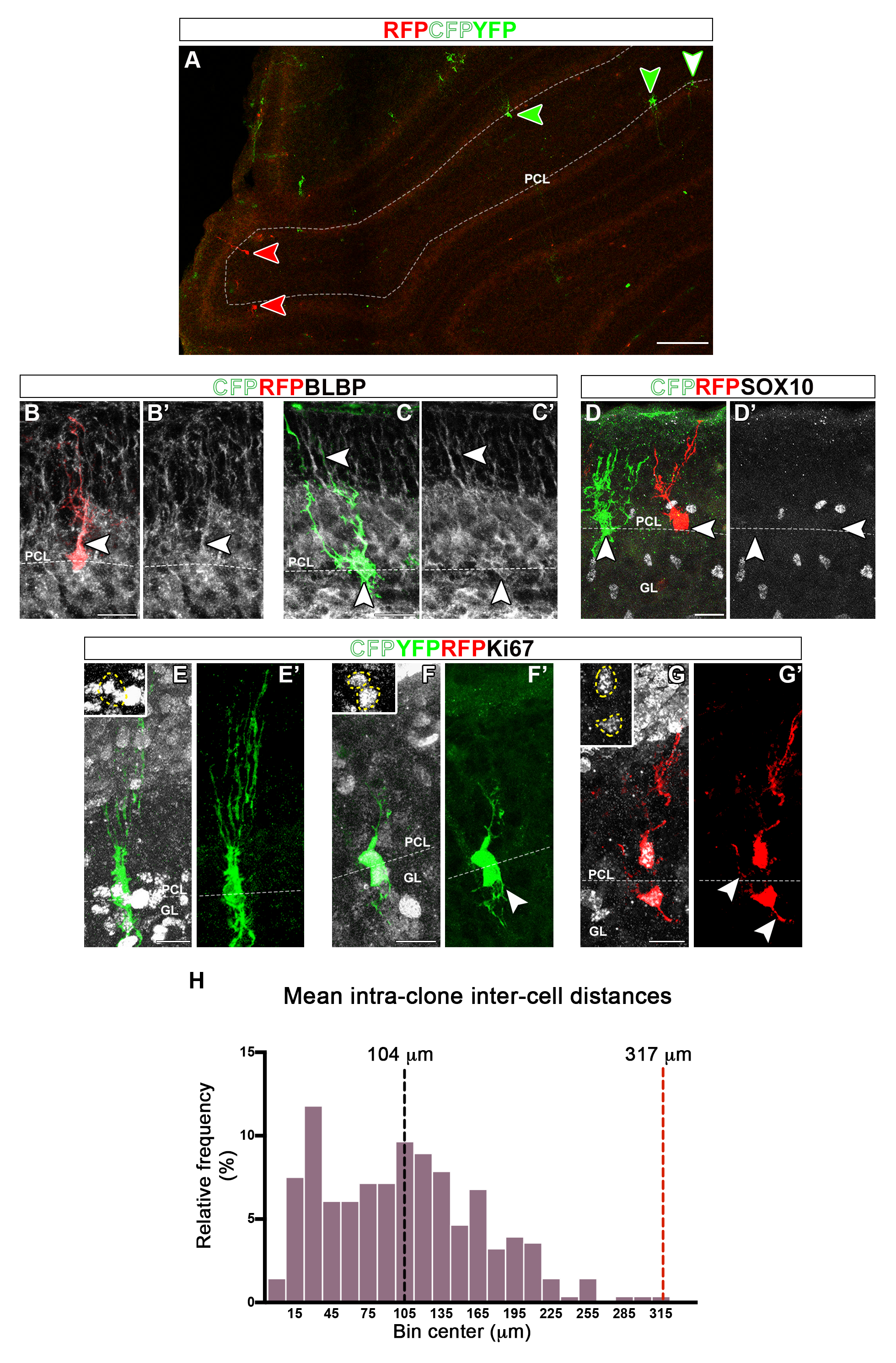

Supplement: S7 Fig — (A) Low magnification of lobule IV–V 48 h after local administration of Tx at P6. Arrows point to sparse PCLps labeled with different Confetti colors (RFP, red arrowheads; YFP, green-filled arrowheads; CFP, white arrowhead with green contour). The position of the cell body in the PCL, the radial morphology and the expression of the astroglial marker BLBP (B-C’) confirm that cells tagged by Tx are PCLps. As expected, PCLps are negative for the oligodendroglial marker SOX10 (D, D’). (E-G’) At short term, the vast majority of the cells (about 80%) are single PCLp, but some pairs of sister cells are also visible. They are composed of 2 juxtaposed cells of the same color. In some cases, they are about to complete a mitosis (nuclei in late telophase in E and relative inset), or splitting apart (F,F’). In other cases, both cells are still in the cell cycle; as assessed by Ki67 expression, they display similar configuration of the nuclei and a mirror morphology, elements indicative of cell division (G, G’ and relative insets). Yellow dotted lines in the insets in E, F, G highlight Ki67+ nuclei of duplets. One of the 2 cells in pairs always displays a PCLp feature, while the other one, in some cases, seems to extend stellate-like processes, suggestive of a GLA fate (arrowheads in F’ and G’). The histogram in H represents the distribution of the mean extensions of clones identified at P30 (single-cell clones were excluded; bin size = 15 μm). The estimated minimal distance between clones (317 μm, red dotted line) is significantly higher than the mean clone extension (104 μm, black dotted line; P < 0.001), and only a small fraction of clones have an extension close to the minimal interclone distance (see Methods). Scale bars 20 μm and 100 μm in A. The numerical data used in panel (H) are included in S1 Data. BLBP, brain lipid–binding protein; CFP, cyan fluorescent protein; GL, granular layer; P, postnatal day; PCL, Purkinje cell layer; PCLp, Purkinje cell layer precursor; RFP, [file pbio.2005513.s007.tif]

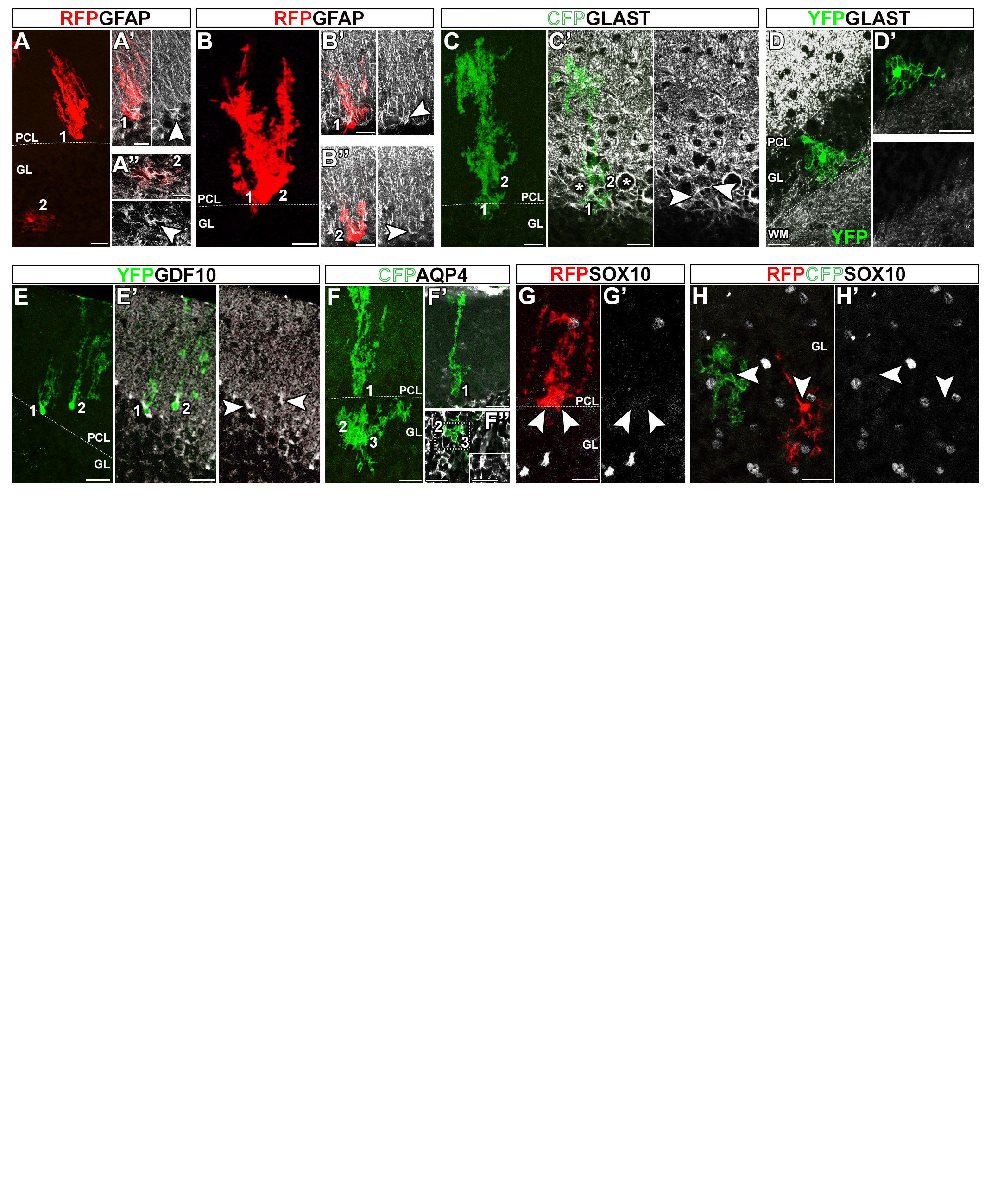

Supplement: S8 Fig — (A,B) Cells labeled after in situ Tx administration in Confetti mice are GFAP+ astrocytes in the PCL (A1 and B1,2) or in the GL (A2). (C-F) The morphological and spatial criteria used to identify BG or GLA are validated by the expression of astrocyte type–specific markers [7,76] (see S1 Table). BG (C-C’), but not GLAs (D-D’), express high levels of GLAST and are also positive for the BG-specific marker GDF10 (E-E’). Asterisks in C’ highlight the cell body of GLAST-negative Purkinje cells surrounded by GLAST-positive BG processes. (F-F”) On the contrary, AQP4 stains exclusively astrocytes in the GL (F2,3) but not BG (F1). Both BG (G-G’) and GLAs (H-H’) do not express the oligodendrocyte marker Sox10, confirming their astrocytic identity. A’-A”, B’-B”, C’-D’,E’,F’, F” are single-step confocal images that more clearly demonstrate the specificity of the different stainings in Confetti-positive or negative (G’-H’) cells. Arrowheads point to Confetti+ cells. Scale bars: 20 μm. AQP4, aquaporin 4; BG, Bergmann cell; GDF10, growth differentiation factor 10; GL, granular layer; GLA, granular layer astrocyte; GLAST, glutamate aspartate transporter; P, postnatal day; PCL, Purkinje cell layer; SOX10, SRY-box 10; Tx, tamoxifen; WM, white matter. (TIF) [file pbio.2005513.s008.tif]

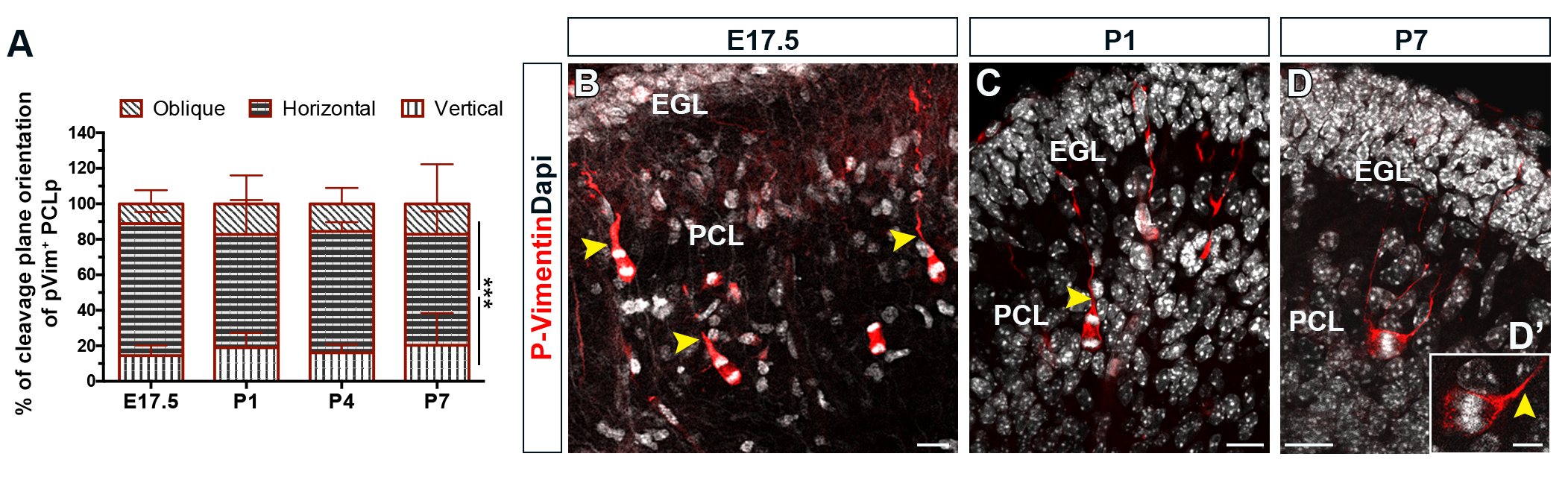

Supplement: S9 Fig — (A) Frequency distribution of the cleavage plane orientations in pVimentin+ PCLp at different time points. PCLps preferentially divide with a cleavage plane horizontal to the PCL throughout cerebellar development (***, P < 0.001). No statistically significant differences are found in the distribution of cleavage plane orientations over time (P = 0.107, main effect of time). (B) During late embryonic phases, radial progenitors delaminate from the VZ and start to colonize the developing PCL and keep dividing through horizontal divisions. (C-D’) After birth, proliferating PCLps still maintain a horizontal cleavage plane, independently of the position of the radial process (highlighted by yellow arrowheads). (D’) Magnification of a single confocal plane of the pVimentin+ cell in D to show the cleavage plane orientation of the nucleus during telophase. P values are computed with GEE analysis. Scale bars: 20 μm (B-D), 10 μm (D’). The numerical data used in panel (A) are included in S1 Data. EGL, external granular layer; GEE, generalized estimated equations; PCL, Purkinje cell layer; PCLp, Purkinje cell layer precursor; pVimentin, phosphorylated Vimentin; VZ, ventricular zone. (TIF) [file pbio.2005513.s009.tif]

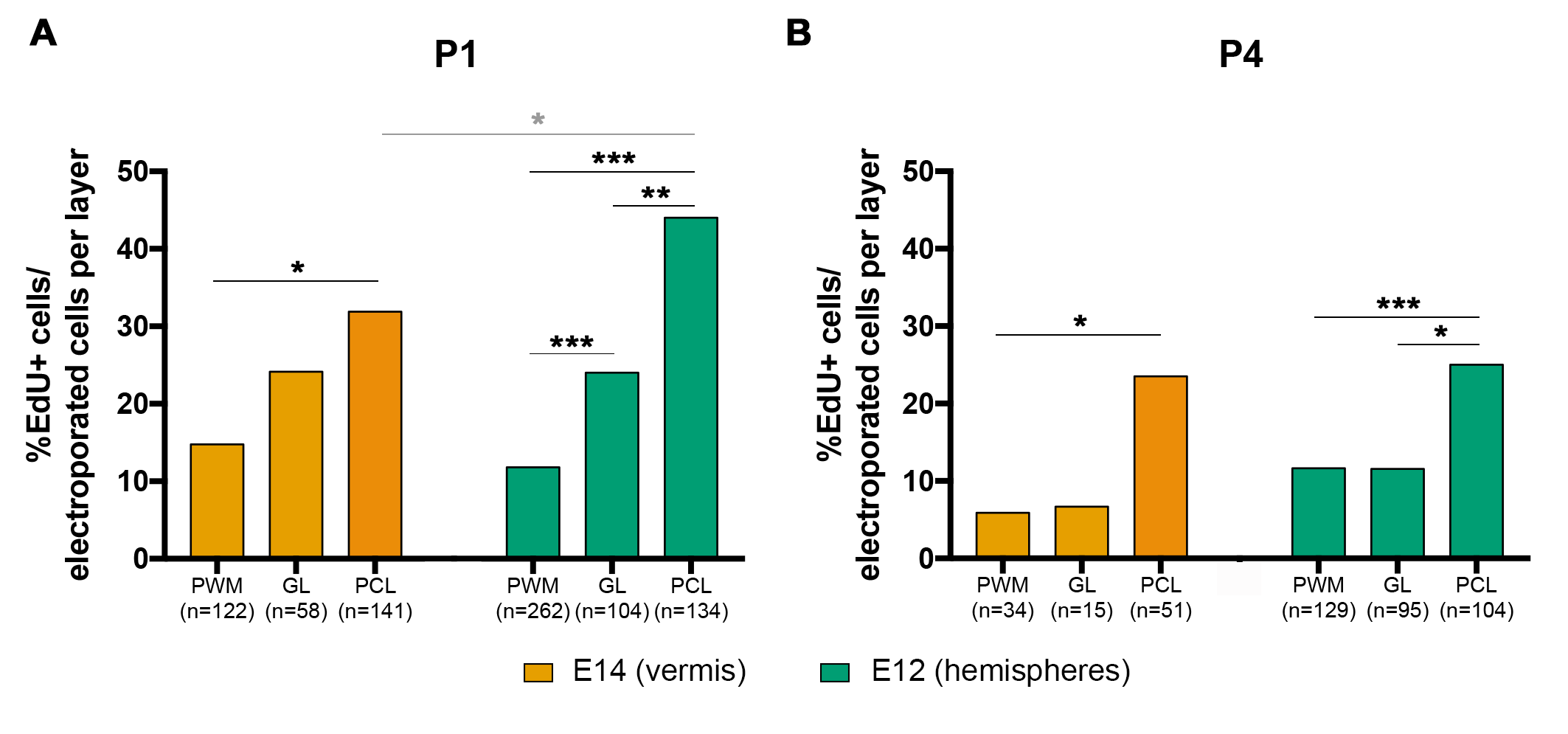

Supplement: S10 Fig — Analysis of active proliferation was performed on E12 hemispheric (green) or E14 vermian (orange) StarTrack-tagged cells in different layers during early postnatal development. Mice were administered twice with EdU with a 3-h interval, and the percentage of EdU-incorporating astrocyte precursors over the total amount of StarTrack-labeled cells in each layer was calculated. At both P1 (A) and P4 (B), the tagged progenitors show a layer-specific pattern of proliferation that declines over time. E12-tagged (green) astrocyte progenitors in the PCL show a slightly higher proliferation activity at P1 (A) compared to those electroporated at E14 (orange). On the other hand, at P4 (B), E12-tagged progenitors in the PWM and GL show a trend to be more proliferative compared to their E14-tagged counterparts, although the low number of cells does not allow to reveal a statistical significance. *, P < 0.05; **, P < 0.01; ***, P < 0.001; P values are calculated with Fisher’s exact test. n = number cells. The numerical data used in the figure are included in S1 Data. E, embryonic day; EdU, 5-ethynyl-2′-deoxyuridine; GL, granular layer; P, postnatal day; PCL, Purkinje cell layer; PWM, prospective white matter. (TIF) [file pbio.2005513.s010.tif]

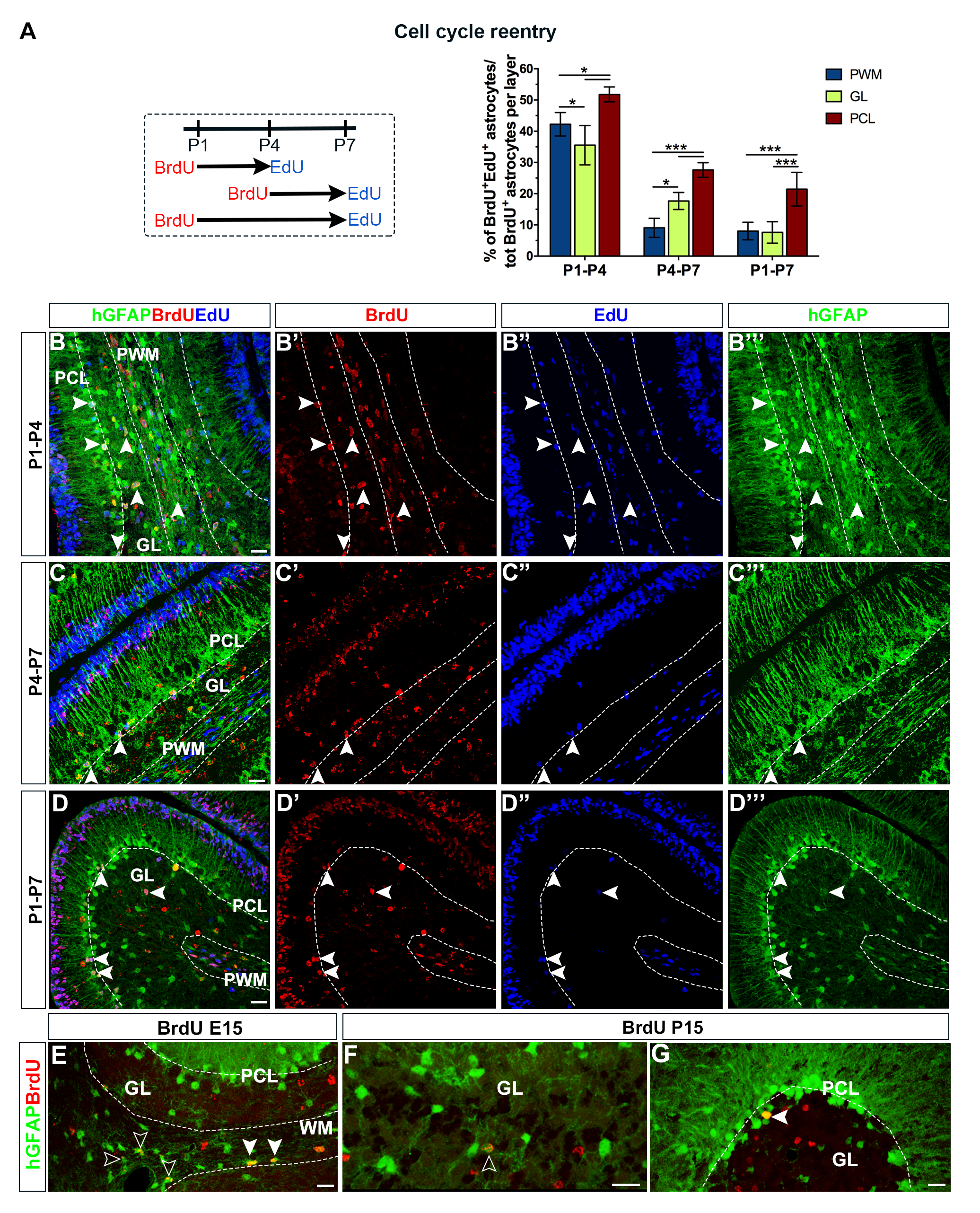

Supplement: S11 Fig — (A) Experimental design: BrdU was injected at P1 or P4 in hGFAP-GFP mice and EdU 6 h before killing at P4 or P7. Triple-labeled cells analyzed in the vermis were plotted as the percentage of total BrdU+/hGFAP+ cells per layer. At each time point, PCLps reenter more frequently in the cell cycle compared to astrocyte precursors in other layers. In the PWM from P4 on, there is a significant drop in the proportion of astrocytes performing another division. The same trend is also present, though less evident, in the GL. (B-D’’’) Images represent sagittal sections of cerebella at the different time points analyzed after double thymidine analogue labeling: (B-B’’’) P1–P4, (C-C’’’) P4–P7, and (D-D’’’) P1–P7. Merged and single channels for BrdU (red), EdU (blue), and GFP (hGFAP, green) stainings are presented. Arrowheads point to some triple-labeled cells. (E-G) Representative images of P30 cerebella of mice injected with BrdU at the beginning (E15, E) and the end (P15, F,G) of astroglial development. Full and empty arrowheads indicate cells with BrdUhigh or BrdUlow positivity, respectively. Plots represent data averaged from distinct animals. *, P < 0.05; ***, P < 0.001, calculated with GEE analysis. Scale bars: 30 μm. The numerical data used in panel (A) are included in S1 Data. BrdU, bromodeoxyuridine; EdU, 5-ethynyl-2′-deoxyuridine; GEE, generalized estimated equations; GFP, green fluorescent protein; GL, granular layer; hGFAP, human glial fibrillary acidic protein; P, postnatal day; PCL, Purkinje cell layer; PCLp, Purkinje cell layer precursor; PWM, prospective white matter; WM, white matter. (TIF) [file pbio.2005513.s011.tif]

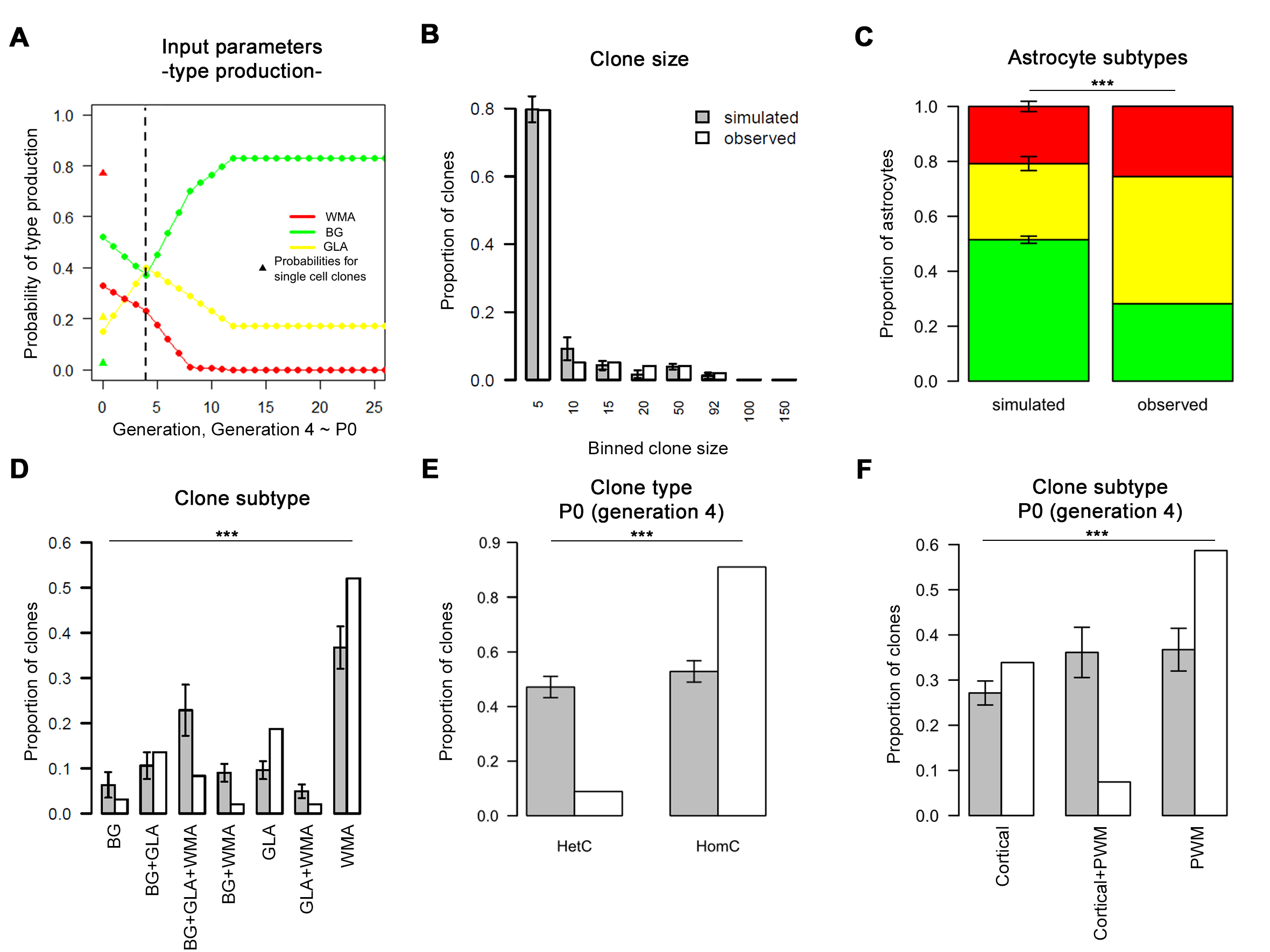

Supplement: S12 Fig — The probabilities for a differentiating progenitor of generating the distinct astrocyte subtypes (BG versus GLA versus WMA) are generation-dependently set according to the birthdating experiments performed in the vermis, as shown in (A). Histograms in (B-D) show the outcomes of the simulated lineages compared to the experimental data. Simulated clone sizes (B) appear quite similar to those of the observed clones. On the other hand, the model fails to recapitulate the proportions of astrocyte subtypes, with the production of too many BG (C; same color code as in A). Similarly, the model fails with the proportions of clone subtypes (D). (E,F) Simulated and observed lineages were compared at P0 (corresponding to generation 4). Too many HetCs (E) are simulated compared to empirical clones, because of the generation of too many PWM+cortical clones at the expenses of either cortical and PWM families (F). ***, P < 0.001, P values were calculated with chi-squared test. Cortical clones comprise PCLp HomCs, GLAp HomCs and PCLp+GLAp HetCs; Cortical+PWM clones comprise PCLp+PWM, GLAp+PWM, and PCLp+GLAp+PWM HetCs. The numerical data used in panels (A-F) are included in S1 Data. BG, Bergmann glia; E, embryonic day; GLA, granular layer astrocyte; GLAp, granular layer astrocyte precursor; HetC, heterogeneous clone; HomC, homogeneous clone; P, postnatal day; PCLp, Purkinje cell layer precursor; PWM, prospective white matter; WMA, white matter astrocyte. (TIF) [file pbio.2005513.s012.tif]

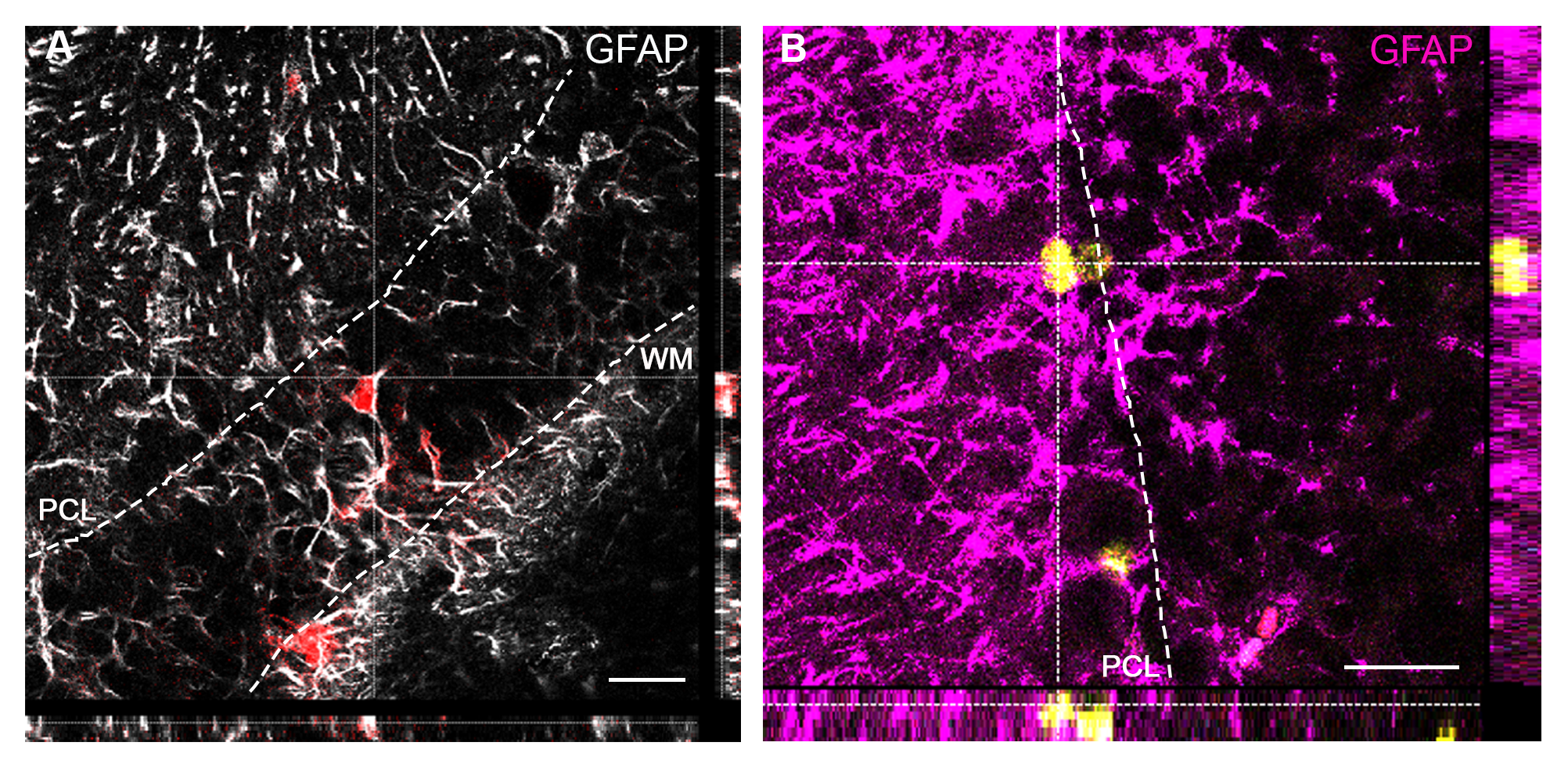

Supplement: S13 Fig — (A,B) Reslices of single step images of P30 clones after anti-GFAP staining (white/purple) unequivocally show the astrocytic identity of cells labeled solely with nuclear markers. Scale bars: 30 μm. GFAP, glial fibrillar acidic protein; P, postnatal day. (TIF) [file pbio.2005513.s013.tif]

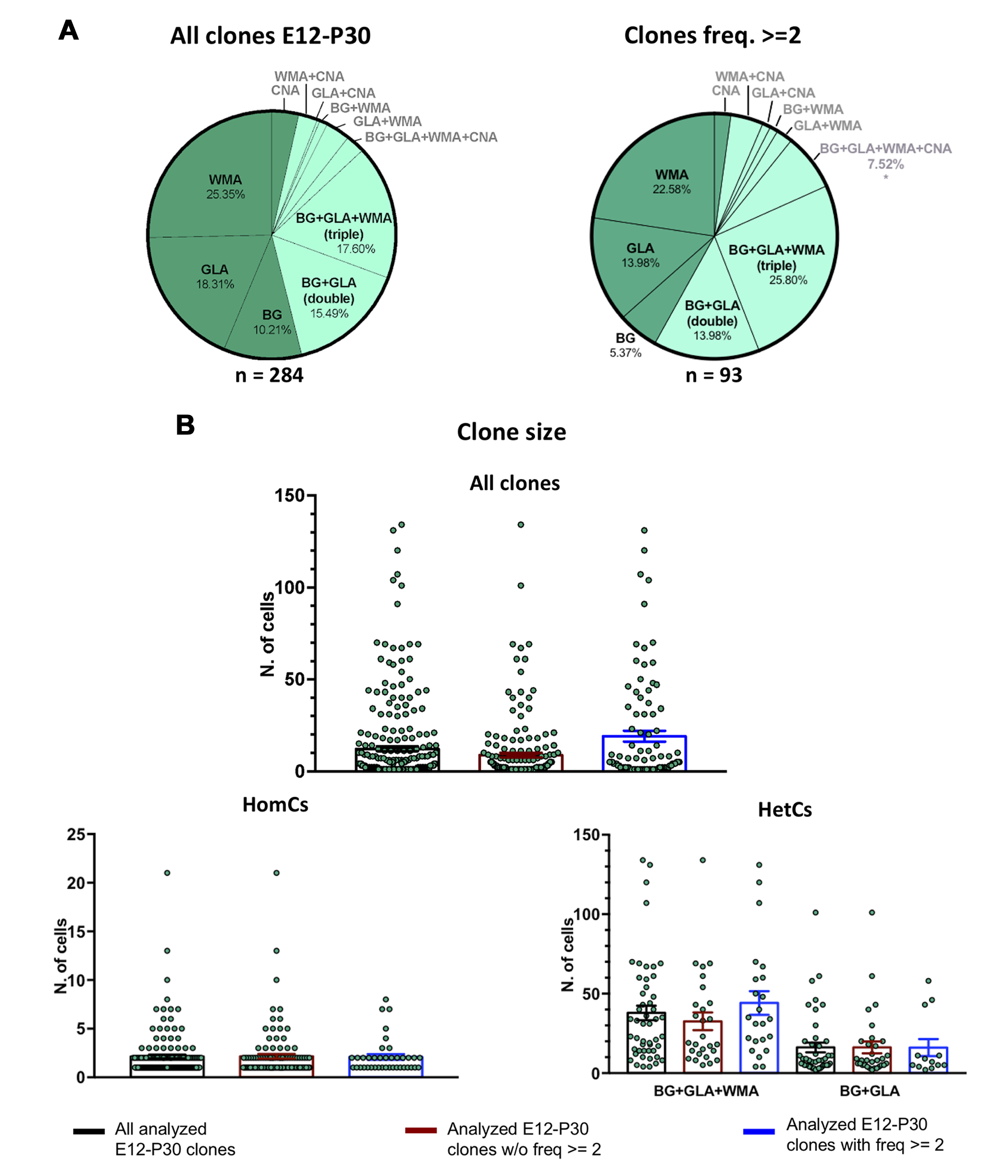

Supplement: S14 Fig — The repeated frequency of some combinations suggests that these may be less reliable to define sibling cells in our samples. Therefore, we assessed whether the clones defined by these repeated combinations in the samples found more prone to lumping errors (2 samples of E12 clones that underwent cluster analysis) belonged to specific clone types/subtypes and displayed features introducing a systematic bias in the analyses. (A) Repeated combinations were homogeneously represented among the distinct clone types/subtypes, with the only exception of BG+GLA+WMA+CNA clones, in which they were enriched (chi test, P = 0.025). However, this clone type, being very rare, was not included in quantitative analyses. (B) Clones defined by repeated combinations (blue bars) were not different from the whole populations (black bars) and behaved as clones with unique combinations (red bars) in terms of clone size (P > 0.05), which is a key clone feature. These results suggest that the presence in the examined samples of clones labeled by repeated combinations does not significantly affect results. The numerical data used in panel (B) are included in S1 Data. E, embryonic day; HetC, heterogeneous clone. (TIF) [file pbio.2005513.s014.tif]

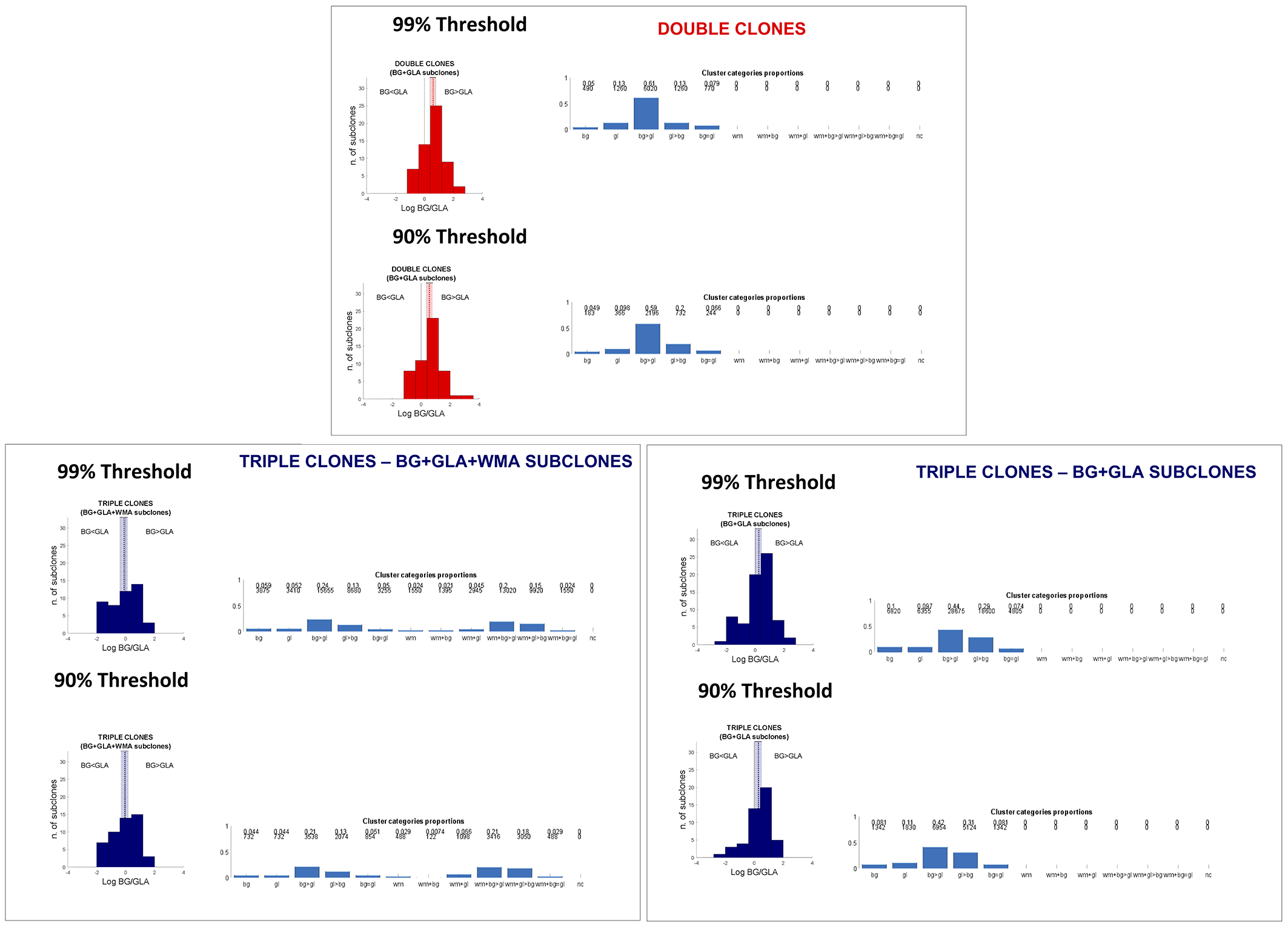

Supplement: S15 Fig — Because of the possible presence of a nonnegligible clustering error, we estimated the impact of a subclone identification bias by rerunning the cluster analysis varying by 20% the number of identified subclones (this was empirically achieved by using 90% and 99% accounted variance thresholds). The ensuing changes in subclone type distributions and ratio were negligible (cf. Fig 5E–5H). The numerical data used in the figure are included in S1 Data. (TIF) [file pbio.2005513.s015.tif]
