# Supplementary material for: Multiple origins and modularity in the spatiotemporal emergence of cerebellar astrocyte heterogeneity
Source: PLoS Biol. 2018 Sep 27;16(9):e2005513. doi: 10.1371/journal.pbio.2005513 (PMC6178385; doi:10.1371/journal.pbio.2005513)
Supplement: S1 Table — (DOCX) [file pbio.2005513.s016.docx]

| **Astrocyte type** | **Morphology** | **Layering** | **Marker expression** | **Type specific function** | **References** |
| --- | --- | --- | --- | --- | --- |
| **Bergmann glia** | Several ascending processes spanning radially the molecular layer – polarized morphology. | Somata aligned to the Purkinje cell layer | **GFAP**  Vimentin  High **GLAST**  High **KIR4.1**  Low **AQP4**  **Gdf10**  GluA1;GluA4  Patched 1,2  Gli1 | Migration of granule cell precursors.  Maturation of PC dendrites and synapses  Homeostasis of extracellular glutamate and neurotransmitters at Purkinje cell synapses. | [1-11] |
| **Granular layer astrocytes** *(Velate* cells) | Star shaped “bushy” processes with lamellar appendage. | Located in the granular layer. | **GFAP**  Low **GLAST**  Moderate **KIR4.1**  High **AQP4** | Proposed to isolate synaptic complexes and partition subsets of mossy fibers conveying different information (direct evidence is still missing). | [1,5,8,12,13] |
| **White matter astrocytes** | Fibrous astrocytes with an elongated morphology and processes oriented along the direction of axons. | Located in the white matter | High **GFAP**  moderate **GLAST**  Very low **KIR4.1**  High **AQP4** | Regulation of myelination  Glutamate homeostasis | [5,8] |

**S1 Table. Heterogeneity in major cerebellar astrocyte types** The table summarizes evidence on morphological, spatial, molecular and functional heterogeneity in major astrocyte types of the cerebellum. Markers highlighted in bold have been used to validate the astrocyte identity and the type-specific immunoprofiles of the examined cells (see S2 Fig and S8 Fig)

**References**

1. Palay S, Chan-Palay V. Cerebellar Cortex. New York: Springer-Verlag Berlin Heidelberg; 1974.

2. Ramon y Cajal S. Histologie du Système Nerveux de l’Homme et des Vertébrés. Maloine, Paris.; 1911.

3. Buffo A, Rossi F. Origin, lineage and function of cerebellar glia. Prog Neurobiol [Internet]. 2013 Oct [cited 2016 Jun 9];109:42–63. Available from: http://www.ncbi.nlm.nih.gov/pubmed/23981535

4. Saab AS, Neumeyer A, Jahn HM, Cupido A, Simek AAM, Boele H-J, et al. Bergmann glial AMPA receptors are required for fine motor coordination. Science. 2012 Aug;337(6095):749–53.

5. Tang X, Taniguchi K, Kofuji P. Heterogeneity of Kir4.1 channel expression in glia revealed by mouse transgenesis. Glia [Internet]. 2009 Dec 1 [cited 2017 Aug 7];57(16):1706–15. Available from: http://doi.wiley.com/10.1002/glia.20882

6. Mecklenburg N, Martinez-Lopez JE, Moreno-Bravo JA, Perez-Balaguer A, Puelles E, Martinez S. Growth and differentiation factor 10 (Gdf10) is involved in Bergmann glial cell development under Shh regulation. Glia [Internet]. 2014 Oct [cited 2016 May 3];62(10):1713–23. Available from: http://www.ncbi.nlm.nih.gov/pubmed/24963847

7. He L, Yu K, Lu F, Wang J, Wu LN, Zhao C, et al. Transcriptional Regulator Zeb2 is Essential for Bergmann Glia Development. J Neurosci [Internet]. 2018;2674–17. Available from: http://www.jneurosci.org/lookup/doi/10.1523/JNEUROSCI.2674-17.2018

8. Farmer WT, Abrahamsson T, Chierzi S, Lui C, Zaelzer C, Jones E V, et al. Neurons diversify astrocytes in the adult brain through sonic hedgehog signaling. Science [Internet]. 2016 Feb 19;351(6275):849–54. Available from: http://www.ncbi.nlm.nih.gov/pubmed/26912893

9. Li K, Leung AW, Guo Q, Yang W, Li JYH. Shp2-dependent ERK signaling is essential for induction of Bergmann glia and foliation of the cerebellum. J Neurosci [Internet]. 2014 Jan 15 [cited 2014 Dec 16];34(3):922–31. Available from: http://www.pubmedcentral.nih.gov/articlerender.fcgi?artid=3891967&tool=pmcentrez&rendertype=abstract

10. Leung AW, Li JYH. The Molecular Pathway Regulating Bergmann Glia and Folia Generation in the Cerebellum. Cerebellum. 2017 Dec;

11. Shaw G, Osborn M, Weber K. An immunofluorescence microscopical study of the neurofilament triplet proteins, vimentin and glial fibrillary acidic protein within the adult rat brain. Eur J Cell Biol. 1981 Dec;26(1):68–82.

12. Chan-Palay V, Palay SL. High voltage electron microscopy of rapid golgi preparations. Neurons and their processes in the cerebellar cortex of monkey and rat. Z Anat Entwicklungsgesch. 1972;137(2):125–53.

13. Hoogland TM, Kuhn B. Recent developments in the understanding of astrocyte function in the cerebellum in vivo. Cerebellum. 2010 Sep;9(3):264–71.
