# Supplementary material for: Multiple origins and modularity in the spatiotemporal emergence of cerebellar astrocyte heterogeneity
Source: PLoS Biol. 2018 Sep 27;16(9):e2005513. doi: 10.1371/journal.pbio.2005513 (PMC6178385; doi:10.1371/journal.pbio.2005513)
Supplement: S3 Table — (DOCX) [file pbio.2005513.s018.docx]

| **A** | **n. of repetitions** | **n. of combinations** | **n of clones** |
| --- | --- | --- | --- |
|  | 1 | 320 | 320 |
|  | 2 | 52 | 104 |
|  | 3 | 20 | 60 |
|  | 4 | 9 | 36 |
|  | 5 | 6 | 30 |
|  | 6 | 0 | 0 |
|  | 7 | 1 | 7 |
|  | 8 | 2 | 16 |
|  | 9 | 0 | 0 |
|  | 10 | 0 | 0 |
|  | **SUM** | **410** | **573** |

| **B** | **E12-P0** |  | **Lumping error probability** | | **E12 -P30** |  | **Lumping error probability** | |
| --- | --- | --- | --- | --- | --- | --- | --- | --- |
|  | Sample n. | n. of clones | Mean | SD | Sample n. | n. of clones | Mean | SD |
|  | 1 | 27 | 0.0224 | 0.0274 | 1 | 19 | 0.0159 | 0.028 |
|  | 2 | 20 | 0.0167 | 0.0278 | 2 | 155 | 0.0935 | 0.018 |
|  | 3 | 16 | 0.0133 | 0.028 | 3 | 110 | 0.0571 | 0.0232 |
|  | **E14-P0** |  |  |  | **E14-P30** |  |  |  |
|  | Sample n. | n. of clones | Mean | SD | Sample n. | n. of clones | Mean | SD |
|  | 1 | 18 | 0.0151 | 0,028 | 1 | 22 | 0.0183 | 0.0277 |
|  | 2 | 42 | 0.0336 | 0.0261 | 2 | 8 | 0.0064 | 0.0279 |
|  | 3 | 64 | 0.0386 | 0.0243 | 3 | 31 | 0.0255 | 0.0271 |
|  |  |  |  |  | 4 | 41 | 0.033 | 0.0262 |

**S4 Table (A) Star Track repeated color combinations in the examined samples.** List of repetitions found when examining all samples (n=13 animals, comprising both short - P0 - and long term- P30 – analyses). **(B) Probability of Lumping Errors in cerebellar samples.** A vector was designed to calculate the probability of picking more than one time within the same sample the same color combination that could give rise to lumping errors. Monte Carlo simulation was repeated 300,000 times to characterize the probability density function of these events. For each cerebellum, we performed a number of picks that represented the number of clones empirically identified in that cerebellum. The probabilities of obtaining lumping errors are described as mean and standard deviation for each cerebellum. We refer to these values in the text as percentages.
